# A universal deep-learning model for zinc finger design enables transcription factor reprogramming

**DOI:** 10.1038/s41587-022-01624-4

**Published:** 2023-01-26

**Authors:** David M. Ichikawa, Osama Abdin, Nader Alerasool, Manjunatha Kogenaru, April L. Mueller, Han Wen, David O. Giganti, Gregory W. Goldberg, Samantha Adams, Jeffrey M. Spencer, Rozita Razavi, Satra Nim, Hong Zheng, Courtney Gionco, Finnegan T. Clark, Alexey Strokach, Timothy R. Hughes, Timothee Lionnet, Mikko Taipale, Philip M. Kim, Marcus B. Noyes

**Affiliations:** 1 Institute for Systems Genetics, NYU Grossman School of Medicine, New York, NY USA; 2 Department of Biochemistry and Molecular Pharmacology, NYU Grossman School of Medicine, New York, NY USA; 3 Department of Molecular Genetics, University of Toronto, Toronto, Ontario Canada; 4 Donnelly Centre for Cellular and Biomolecular Research, University of Toronto, Toronto, Ontario Canada; 5 Department of Computer Science, University of Toronto, Toronto, Ontario Canada

**Keywords:** Synthetic biology, Genomic engineering

## Abstract

Cys_2_His_2_ zinc finger (ZF) domains engineered to bind specific target sequences in the genome provide an effective strategy for programmable regulation of gene expression, with many potential therapeutic applications. However, the structurally intricate engagement of ZF domains with DNA has made their design challenging. Here we describe the screening of 49 billion protein–DNA interactions and the development of a deep-learning model, ZFDesign, that solves ZF design for any genomic target. ZFDesign is a modern machine learning method that models global and target-specific differences induced by a range of library environments and specifically takes into account compatibility of neighboring fingers using a novel hierarchical transformer architecture. We demonstrate the versatility of designed ZFs as nucleases as well as activators and repressors by seamless reprogramming of human transcription factors. These factors could be used to upregulate an allele of haploinsufficiency, downregulate a gain-of-function mutation or test the consequence of regulation of a single gene as opposed to the many genes that a transcription factor would normally influence.

## Abstract (web summary)

Zinc finger design is facilitated with a deep-learning model.

## Main

Programmable regulation of gene expression would offer both powerful research tools as well as enormous therapeutic potential. Diseases caused by haploinsufficiency, gain-of-function mutations or misexpression of a gene can be directly treated by modification of gene expression^1–3^. While CRISPR–Cas and transcription activator-like effector (TALE)-based tools have been developed for such applications^4–9^, their intrinsic characteristics could limit their therapeutic efficacy. For instance, the size of these proteins^10^ complicates delivery—in particular the use of adeno-associated viruses (AAVs), clinically the best validated delivery method. Moreover, pre-existing immunity in humans for spCas9 (refs. ^11,12^) makes their long-term expression an immunogenic risk.

Research applications designed to probe regulatory mechanisms could also be hampered by the difference in size and spatial arrangement of SpCas9 fusions compared with natural transcription factors (TFs): SpCas9 is five to ten times the size of the most common DNA-binding domains (DBDs) found in human TFs, with the common C-terminal effector domain more than six times further from the DNA^13,14^ (Supplementary Fig. 1). In addition, the presentation of effector domains out of their natural context could impact their function. For example, it is unclear how the repressive potential of KRAB domains differs in isolation compared with their expression in their parent proteins. Further, most synthetic activators use the viral VP16 domain^15,16^ or one of its derivatives, which may not accurately mimic natural activating TFs that can encourage expression through different interactions and spatial arrangements. Finally, the effect of artificial regulators was shown to be highly dependent on binding position; differences as little as a single base can have a large impact^17^, potentially restricting CRISPR-based tools due to their protospacer adjacent motif (PAM) limitations.

By contrast, the Cys_2_His_2_ zinc finger (ZF) domain offers unique advantages for targeting of effector domains to the desired genomic loci^18,19^. ZFs require <170 amino acids to specify a unique sequence in the human genome, enabling routine—even multiplexed—delivery by AAVs. In addition, ZF domains are less likely than SpCas9 to be immunogenic because nearly 50% of human TFs use this DBD to specify their genomic targets^20^. In fact, 343 human ZF TFs utilize KRAB domains^21^ for repression while dozens of others are known to activate transcription^20,22,23^. While the potential utility of designer ZF arrays has long been recognized, their engineering has remained challenging with no proper design code having emerged thus far. This is not for lack of effort, because multiple approaches have been used to generate ZF libraries^24–26^ and ZF modules^27,28^ to provide designer ZF arrays. However, these approaches either require multiple rounds of laborious selection that produce ZFs with inconsistent activity or the application of preselected modules that often fail when expressed out of their selected context. Conversely, a proper code for ZF array design could enable the reprogramming of natural ZF TFs to provide tools that can activate or repress target genes and that are sufficiently small for multiplexed delivery in AAVs with minimal risk of immunity.

Here we found a comprehensive survey of adjacent finger influences that enabled a design model for ZF arrays, and show that these arrays can seamlessly reprogram transcription factors. By testing multiple TFs, we demonstrate that their reprogramming with designer ZFs is a robust approach to commandeer the function of TFs that activate or repress for the regulation of reporter genes or genes directly from their genomic loci.

## Results

### Selection of ZF specificity and compatibility

**Fig. 1:**
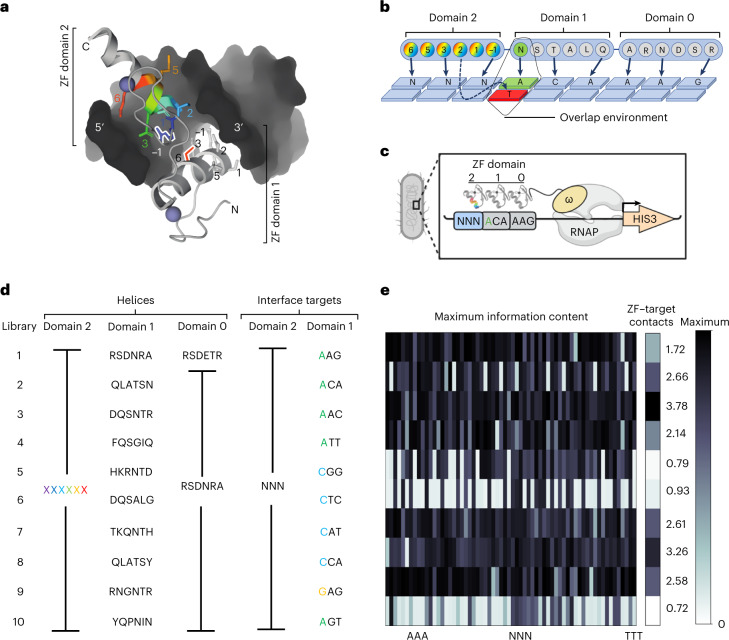
Overview of interface-focused ZF screens. **a**, Structure of adjacent ZF domains showing their close proximity. Helical position 6 of domain 1 (red) and position −1 (blue) of domain 2 are outlined. **b**, Cartoon of interactions between adjacent helices and DNA. The six helical positions of the three domains are shown as circles, with the common contacts made by positions −1, 2, 3 and 6 indicated by arrows. The overlap environment, which includes the base adjacent to the library interaction and the amino acid used to specify that base, is highlighted in green. This environment is unique for each library. **c**, Cartoon of B1H selections. The three-fingered protein is expressed as a C-terminal fusion to the omega subunit of RNA polymerase. For each library, ZF domain 2 is randomized at six helical positions and screened for amino acid combinations able to specify each of the 64 possible NNN targets. This is done in 64 independent screens. Domains 0 and 1 bind to their known, preferred targets and thereby anchor the protein adjacent to the NNN target sequence and present an overlap environment unique to that library. Only helices able to bind the target in the unique library overlap environment will recruit the polymerase, activate the reporter and survive on selective media. **d**, Left, helical residues for domains 0, 1 and 2 are shown for each library screened. Domain 2 contains all possible combinations of the six helical residues whereas domain 1 is fixed in the selections but varied by library. The sixth residue of domain 1 is the side chain that will be exposed at the interface between domains 1 and 2. Domain 0 is the same in all libraries except library 1. Right, there are 64 DNA targets for domain 2 to be screened against in 64 independent selections. The fixed targets for domain 1 of each library are shown, with the overlap base color coded by nucleotide. **e**, Left, to assay the success of each selection we determined clusters from the data for each selection. Here we show the maximum information content at one position of the strongest cluster to provide a relative measure of enrichment across all selections. Right, molecular dynamic simulations were performed on all domain 1 helices in their previously characterized contexts. The number of suggested contacts between domain 1 and the DNA is shown for each library.

Two general approaches have been used to engineer ZFs with novel specificity (Supplementary Fig. 2). The first focused on engineering one finger at a time by selection of functional variants from ZF libraries where the six base-specifying positions of the helix have been randomized (Supplementary Fig. 2b). The second approach focused on the interface between adjacent ZFs of an array, because the influence of adjacent fingers on one another has been apparent since the first structures of ZFs bound to DNA were solved (Supplementary Fig. 2c); this influence leads to combinatorially greater complexity, which is the main reason for the failure of previous attempts to build a code. While the first approach allows for a comprehensive screen of all amino acid combinations at the six critical positions of the ZF alpha-helix^24,26,27,29–32^, it samples these combinations only in a single-adjacent-finger context. As a result, only ZF strategies enabled by this initial selection environment are available in subsequent rounds of selection or as the foundation of a ZF model. By contrast, the second approach captures the complexity of compatibility at the interface between ZFs^25,28,33^ (Supplementary Fig. 2c). However, because combinatorial explosion quickly exceeds the maximum practical library size for any screening platform, incomplete randomization schemes and the sampling of a limited number of helical positions become necessary. We hence reasoned that the solution lies in a combined approach that uses multiple exhaustive libraries in a comprehensive set of interface environments. In other words, each library presents a fully randomized single ZF helix in a unique interface environment, producing a broad catalog of binding strategies that are enabled by that single-interface environment. When considered across all libraries screened, this laborious but inclusive approach produces a comprehensive portfolio of general and interface-specific ZF binding solutions (Fig. 1). We theorized that this interface-derived complexity would provide (1) the diversity necessary to generate compatible ZF pairs able to bind a wide range of DNA targets and (2) the depth of data required to support a model for ZF array design.

The profound influence exerted by adjacent ZFs on one another can be explained by the multiple side chains of adjacent ZFs that bind DNA in close proximity to one another. This is most obvious at the ‘overlap’, where position 6 of an N-terminal helix can be within hydrogen-bonding distance of positions −1 and 2 side chains of its C-terminal neighbor (Fig. 1b). In this way the N-terminal helix is presenting a specific interface interaction, or ‘environment’, to its C-terminal neighbor that is based on the side chain employed and the base specified at the overlap position (Fig. 1a,b). Therefore we screened ten ZF libraries, each presenting the randomized C-terminal helix in a unique adjacent finger environment defined by the adjacent ZF helix (Fig. 1c,d). We screened these libraries across each of the 64 possible three base pair (bp) targets in independent selections to recover functional ZF helices. Each library presents a unique interaction between the side chain at position 6 of the adjacent finger and the base it specifies at the overlap that defines the unique adjacent finger influence of each library (Fig. 1d and Supplementary Fig. 3). We designed the majority of our libraries to contact adenine or cytosine at the overlap, to provide a contrast to the arginine–guanine contacts that presented at the overlap in most previous ZF screens. In addition, two of our libraries can specify two different bases at the overlap (nos. 1-A or -C and 3-A or -G). Therefore, we completed two comprehensive screens of these libraries, one with each base presented at the overlap. In total, we screened >49 billion protein–DNA interactions from ten libraries, across 12 sets of 64 selections per library, for 768 independent selections.

From these screens we found global and target-specific differences induced by library environments, indicative of the strength of the constraint exerted by each adjacent finger context on the selected ZF. The total number of selected helices ranged from 128,000 to >1 million per library screened (Supplementary Fig. 3). To distinguish selections that appeared to have low enrichment because of overlapping but unique strategies to bind the selection target from selections that truly failed to enrich functional helices, we used reasoning based on information content. We first used MUSI^34^, a method designed to identify multiple, unique sequence clusters in complex datasets such as these. We then quantified the information content across motifs generated from the different sequence clusters recovered in each selection. Reasoning that a successful selection should produce clusters where at least one helical position has been strongly selected for, we removed selections lacking any clusters with at least one position with high information content (Supplementary Fig. 3). We used this same threshold, the maximum information content at a single helical position of a cluster, to quantitatively compare different libraries (Fig. 1e). From this analysis we found that 39–100% of the 3-bp target selections led to successful enrichment of ZFs, depending on the library (Supplementary Fig. 3). In fact, for nine of the libraries screened at least 55 of the 64 selections (>85%) successfully enriched ZFs with specific sequence composition. In addition, for each of the 64 three-base-pair targets, at least eight different library contexts resulted in successful enrichment of ZFs, demonstrating the ability of ZFs to bind any 3-bp target in a wide range of adjacent finger environments. Also note that at least one library that bound either A, C or G at the overlap successfully enriched helices in at least 61 of the 64 selections (for example, library 1 with an A overlap, library 7 with a C overlap and library 9 with a G overlap), suggesting that functional ZFs exist in a wide variety of contexts independent of the overlap base. By contrast we found libraries 6 (C overlap) and 10 (A overlap) to be the least successful (Supplementary Fig. 3). To assess the impact of the biophysical properties of the adjacent helix on library success, we performed molecular dynamics simulations using the helices utilized in library contexts. We found that the number of contacts between the adjacent finger (domain 1 in Fig. 1d) employed in each library and the DNA it specifies were related to global library success, indicating that higher affinity of the neighboring finger enables more ZF strategies (Fig. 1e). Hence, adjacent fingers have a large impact on ZF function while viable ZF binding strategies exist for each overlap base.

### G-rich binding modularity and promiscuity

**Fig. 2:**
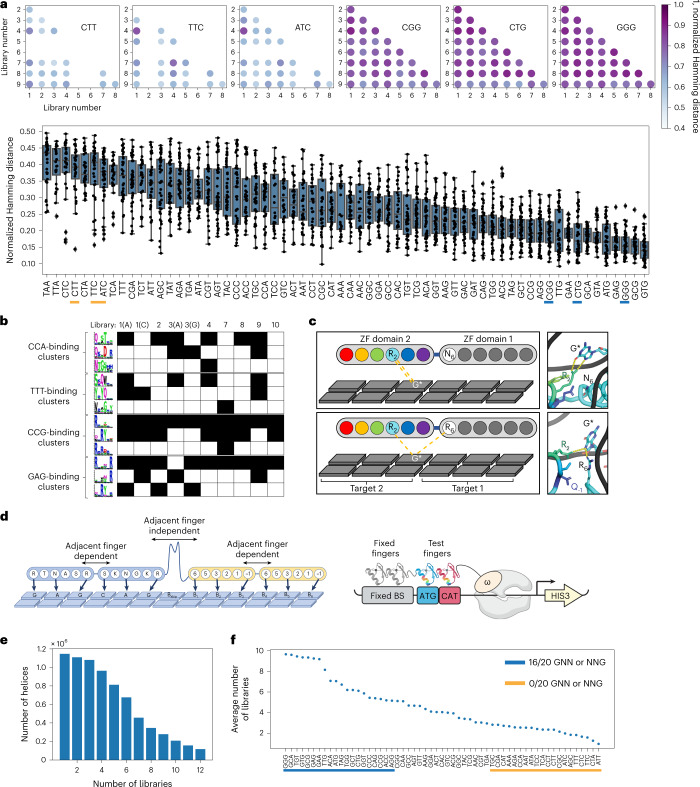
Specificity solutions are library specific. **a**, Top, dot plot comparison of 1 Hamming distance is provided comparing the similarity of helical strategies enriched in libraries 1–9 for three G-rich targets (right) and three G-poor targets (left). The darkness of the dot represents the similarity of the enriched populations, with darker dots being more similar. Empty spots indicate a failed target selection for one or both of the libraries compared. Bottom, normalized Hamming distance for all libraries across all targets, listed from least similar (left) to most similar (right). The targets compared above are underlined in yellow for G-poor targets and in blue for G-rich targets. **b**, Clusters were determined by MUSI from the enriched helices in each library selection. Three clusters are shown for four different binding sites (CCA, TTT, CCG and GAG). If a cluster was enriched in a library selection, the corresponding box is filled black in the table. **c**, Schematic illustration (top) and molecular dynamics snapshot (bottom) of hydrogen bonds between the arginine at position 2 of the domain 2 helix Qs**R**Ytt with the G* of the CCG* target when an asparagine is at position 6 of the adjacent finger (library 2 environment), or when an arginine is at position 6 of the adjacent finger (library 3, 9 environment). **d**, Left, paired format for two-finger selections using the base-skipping linker to encourage modularity, allowing test pairs (yellow) to function independently from the fixed pair (blue). Right, cartoon of B1H two-finger selections. **e**, The number of helices enriched in two-finger selections is shown as a factor of the number of single-finger libraries in which they originated. **f**, Comparison of helices enriched in the two-finger selections showing average number of single-finger libraries in which a helix originated, by binding site.

The majority of published ZF selections have been carried out with an arginine–guanine contact presented at the overlap, due to the high affinity offered by this contact and its historical presence in the parent protein scaffolds that were used to engineer specificity. Consequently we reasoned that the libraries reported here presenting adenine and cytosine contacts at the overlap would enrich novel types of ZF-binding strategies. Therefore, to measure the similarity of helices enriched in different library contexts we computed pairwise Hamming distances (normalized by sequence length) between all helices enriched for each successful 3-bp target selection across all different library contexts. We then compared the mean normalized Hamming distance for each of the 3-bp targets to compare library differences. While there were general trends that libraries employing the same overlap base were more similar (Supplementary Fig. 4), the most striking difference was found when comparing libraries with adenine and cytosine at the overlap with the two libraries displaying an arginine–guanine contact at the overlap (Supplementary Fig. 4c). The arginine–guanine contact libraries (3 (G) and 9) were more similar to each other than any other libraries screened. Interestingly, a comparison of helices selected to bind various targets across all libraries showed that G-rich binding is less influenced by library context (Fig. 2a and Supplementary Fig. 5). This suggests that G-rich binding is more modular, because these helices appear less dependent on the adjacent finger interaction. However, this independence in binding could lead to more promiscuity. To address this possibility, we calculated how frequently helices recovered in a particular 3-bp target selection were recovered in other target selections. The 15 targets with the greatest target selection entropy (that is, recovered in the majority of other selections) all had a G at the GNN or NNG position, where arginines were the dominant amino acid enriched at corresponding positions 6 and −1, respectively (Supplementary Fig. 6). Conversely, none of the 13 targets with the lowest target selection entropy had a G at these positions. These results demonstrate that helices binding a G at either the first or third position of a binding site are more likely to be promiscuous ZFs. This could help explain the G-rich bias in ZFs previously selected, engineered or assembled as modules. This may also suggest that these modules tend towards more off-target binding.

### General and specialized binding strategies

For a more fine-grained analysis of the differences between libraries, such as the types of binding strategies enabled by one library environment versus another, we compared the clusters generated by MUSI for each target site selection. For most targets we found general strategies common to several successful library selections. We also found specialized strategies recovered in a small number of selections and, in some cases, recovered with only a single library environment (Fig. 2b). Previous work has shown that recovery of helical strategies in one library versus another is indicative of activity only in the recovered contexts, rather than sampling influences^35^. In addition, because different library contexts present different structural influences at the overlap, we investigated the physical influences that might lead to the selection of a particular type of ZF in a specific library context. For example, in most NCG selections we found a cluster of ‘QxRYxx’ helices (see CCG in Fig. 2b). However, this cluster was not recovered in libraries that presented an arginine from the adjacent finger at the overlap (libraries 3 and 9). Molecular dynamics simulations suggested that this is due to potential competition between position 2 of the selected finger and the arginine at position 6 of the adjacent finger (Fig. 2c).

The data demonstrate global and specific differences in ZF function dictated by the adjacent finger environment, but they represent only a small number of potential adjacent finger influences. To test how greater variability at the interface might influence compatibility, we created 200 two-finger libraries by assembling pools of helices successfully selected to bind each 3-bp half-site of a 6-bp target. These pools represented helices preselected to bind each half-site of the target across the small but diverse adjacent finger influences assayed in our primary selections. The 6-bp targets for these 200 two-finger selections were chosen to accommodate the construction of ZF nucleases (ZFNs) that will bind longer sequences in the enhanced green fluorescent protein (eGFP) coding sequence (Supplementary Fig. 10). In this way we were able test and validate the function of the selected two-fingered modules in the context of longer arrays necessary for ZFN activity while providing detailed compatibility data that could be used to train the model. Mimicking single-finger selections, we used two fixed fingers with known specificity to anchor the binding and properly position ZF pairs in the pool library to engage the test 6-bp target (Fig. 2d). To minimize the potential influence of fixed fingers on ZF pairs in the library we employed a long, flexible linker between the fixed and library pairs to encourage independence in binding. This linker prefers a base to be skipped between the binding sites of the fixed and screened ZF pairs, reducing the potential influence of fixed fingers on ZFs in the library and encouraging these pairs to work as independent modules^36^. In this way, the screens should produce ZF pairs that are dependent on one other but also function as an independent module relative to the fixed pair in the array. We selected compatible pairs of ZFs from these 200 libraries and analyzed the number of starting library environments from which the helices were enriched. Most helices enriched in these compatibility assays were recovered in only a minority of the library environments (Fig. 2e). This suggests that, despite the fact that all of these helices were preselected to bind each half-site, only a fraction is enabled in these new environments. Interestingly, when we plotted compatible helices by target selection and examined the number of primary libraries in which they were recovered, we again found that G-binding ZFs recovered in the two-finger selections originated in a large number of the primary libraries while compatible ZFs recovered to bind G-poor targets originated in a small number of library selections (Fig. 2f). Together these results demonstrate that, even for a more comprehensive set of presented environments, the interface has a large influence on ZF function and that G-rich binding helices tend to be more modular and promiscuous. The data from these two-finger library selections offer crucial insight into the pairwise compatibility of individually functional ZFs.

### Hierarchical transformer integrates selection data

**Fig. 3:**
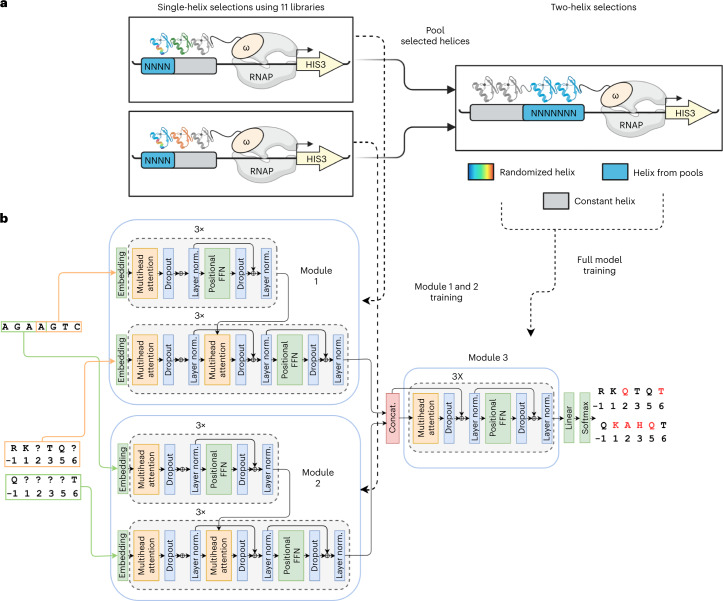
An interface-focused ZF design model. **a**, The model comprises two modules trained on single-helix B1H selections to predict residues in partially masked helices that bind 4-mer nucleotide sequences. **b**, The residue embeddings generated from these modules are fed into a third module that learns interhelix compatibility. The full model is trained on two-helix B1H selection data to predict residues in partially masked helix pairs that bind 7-mer nucleotide sequences. In the model architecture schematic, layer normalization is abbreviated to "layer norm." and concatenation is abbreviated to “concat”.

Despite considerable effort, previous attempts to generate a general ZF design code have failed. However, those attempts were hampered by sparse datasets that ignored adjacent finger influences and/or severely undersampled the potential complexity. Given the unprecedented depth of our screening data, we sought to develop a unique model that explicitly addresses these neighbor finger influences. Also artificial intelligence technology—in particular from natural language processing–vastly outperforms earlier machine learning models at capturing intricate detail in large pools of data. We separately make use of the comprehensive single-finger library selections that describe specificity in a variety of neighbor finger contexts, as well as the 200 pair selections confirming which ZFs are compatible with each other as neighbors (Fig. 3a). This information is by nature hierarchical and, to make optimal use of it, we developed a neural network architecture that implements attention modules in a hierarchical manner (Fig. 3b). The first layer of this hierarchical architecture contains two modules trained on the single-finger selection data for each of the half-sites of a desired two-finger target. Because we consider each target 3-bp plus the adjacent base, this becomes two overlapping 4-bp targets or a 7-bp, two-finger target. The single-helix modules generalize to unseen sequences; interestingly, residue–nucleotide relationships are captured in the attention values (Supplementary Figs. 7 and 8). The residue embeddings from these two modules are then fed into a top module trained on data recovered from the 200 ZF pair selections (Fig. 3b). This is akin to the experimental procedure of taking selection pools from single-finger selections and performing two-finger selections on them (Fig. 3a,b for comparison). In effect, the modules of the first layer design functional single ZFs (for a given neighbor environment) while the second layer module assembles compatible ZF pairs.

**Fig. 4:**
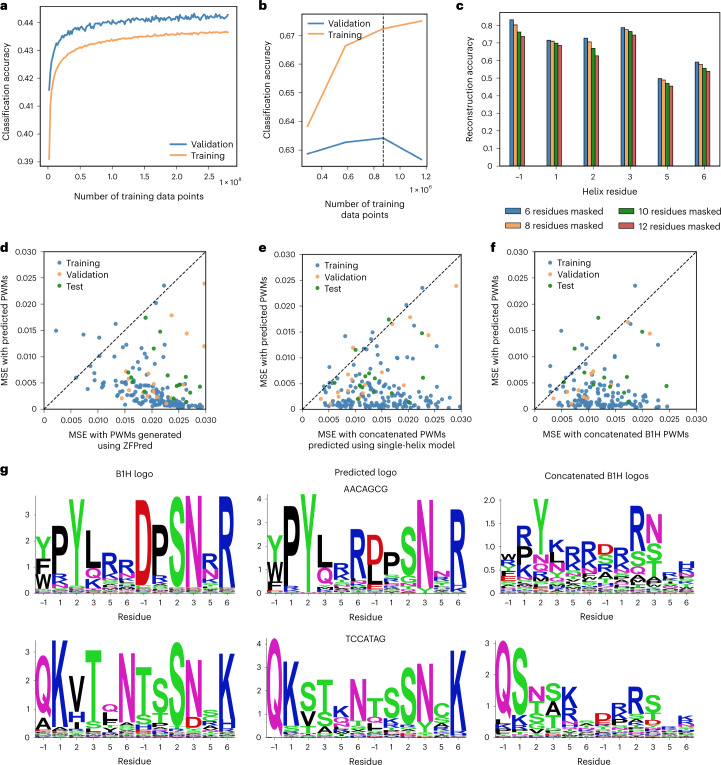
Performance of two-helix design model. **a**, Training and validation accuracy during pretraining step. **b**, Training and validation accuracy during fine-tuning step. **c**, Helix sequence reconstruction accuracy with different numbers of masked residues. **d**, Comparison of differences between predicted and real selection logos using the developed model and ZFPred based on the mean-square error (MSE) of predicted position weight matricies (PWMs) to ground-truth PWMs. **e**, Comparison of differences between predicted and real selection logos using the two-helix model and concatenated logos from the single-helix design model. **f**, Comparison of differences between predicted and real selection logos using the two-helix model and concatenated logos from single-helix B1H selections. **g**, Predicted logos, real B1H logos and concatenated single-helix B1H logos for test set sequences.

The overall model retains a traditional encoder–decoder architecture: An encoder generates a high-dimensional representation for each DNA base and a decoder then generates predictions for each residue in a ZF helix, using self-attention layers and attention layers that relate nucleotide bases to helical residues. The model was trained using the masked language model objective^37^; during training we provided the nucleotide target as well as a partially masked ZF sequence and evaluated cross-entropy loss between predicted residues and the ground-truth ZF sequence (Methods). We achieved reconstruction accuracy (sequence identity to the six masked residues) of 0.62 and 0.69 on the validation and test data, respectively; some positions (such as −1) that were strong determinants of binding specificity had higher reconstruction accuracies (Fig. 4a–c). Overall, because some variability in the 12 residues is allowable while retaining the ability to bind a target sequence, 0.62–0.69 reconstruction accuracy can be considered quite high (Fig. 4c).

### ZFDesign generates compatible ZF pairs

Our method (ZFDesign) generates sequences in an incremental fashion: Starting from an empty sequence, the model is run once for each amino acid in the ZF helix pair. At each iteration an amino acid is predicted, and this prediction is provided as context in subsequent iterations. For optimal sequence generation we adapted both an A*-based sampling method^38^ and a temperature-dependent sampling procedure^39^. We sought to compare ZFDesign with a baseline, but no previous model has explicitly attempted to perform full ZF-array design for a given target and with only a few collections of ZFs available. However, previous models designed to capture ZF binding specificity exist and can be adapted to design ZFs for given targets; we used ZFpred, a recently developed method that outperformed previous models^35^. We then used both ZFDesign and ZFpred to generate ZF sequences to target 6-mers from our test dataset. As alternative baseline comparisons, we first used the single-finger models (for example, only the bottom module in Fig. 3b) to generate ZF sequences for each DNA 3-mer and concatenated them. In a similar fashion, we also took sequences directly from each of our 3-mer bacterial one-hybrid (B1H) selections and concatenated them, which is akin to previous methods of simply concatenating pre-existing collections of fingers as modules. All three methods performed noticeably worse than our hierarchical model (Fig. 4d–f). When directly comparing representative sequence logos of the sequences generated, ZFDesign produced logos that broadly captured those from the B1H two-helix selections whereas the concatenated logos from the one-helix selections were noticeably different (Fig. 4g and Supplementary Fig. 9), underlining the fact that ZFDesign captures interhelix relationships absent from single-helix selections.

For experimental validation of ZFDesign we performed a GFP disruption assay in a U20S cell line previously used to approximate nuclease activity for ZFNs^40^, TALENs^41^ and SpCas9 (ref. ^42^), because indels in the coding sequence of GFP led to frameshifts and loss of fluorescence. For each ZFN, two ZF arrays were designed as ZFNs requiring dimerization of the Fok1 catalytic domain, presented as C-terminal fusions from each ZF array in a tail-to-tail orientation (Supplementary Fig. 10a). The arrays use a longer linker between two-finger modules to enable independent binding, because the linker allows a base to be skipped between the binding sites for each two-finger module^36^. The DNA targets for the two-finger selections detailed above had been specifically chosen to accommodate targets in the GFP coding sequence. Therefore, for each target we first assembled ZFNs that use four ZFs per monomer (eight per ZFN) based on the most frequent pairs recovered in the corresponding two-finger selections. Next, we designed five ZFNs that also use four ZFs per monomer for comparison with the B1H-selected ZFs that bind the same targets. All of the designed ZFNs were functional above background, but four of the five demonstrated decreased activity relative to the selected arrays (Supplementary Fig. 10b). However, the substitution of single modules largely increased activity (Supplementary Fig. 10c), demonstrating the stringency of the assay because a single weak module can have a large impact on overall function. Nevertheless, because these designs were functional on all targets, and longer arrays have overcome the presence of weak modules^43^, we designed and tested 16 ZFNs that use six ZFs per monomer (12 per ZFN). We found all 16 to be functional, with a mean 53.6% loss of fluorescence (Supplementary Fig. 10d). Finally, to determine whether six fingers are sufficient for monomeric binding, we designed a six-finger array to label a genomic locus as a GFP fusion. Because many copies of GFP are necessary to visualize punctate GFP expression, we designed the array to bind a repetitive sequence on chromosome 14, which appears three times in HEK293T cells. We observed three points of GFP fluorescence by live cell imaging (Supplementary Fig. 10e). These results suggest that ZFDesign consistently produces highly functional ZF arrays and that six or more fingers routinely produce strong on-target activity in the human genome.

### Seamless reprogramming of human transcription factors

**Fig. 5:**
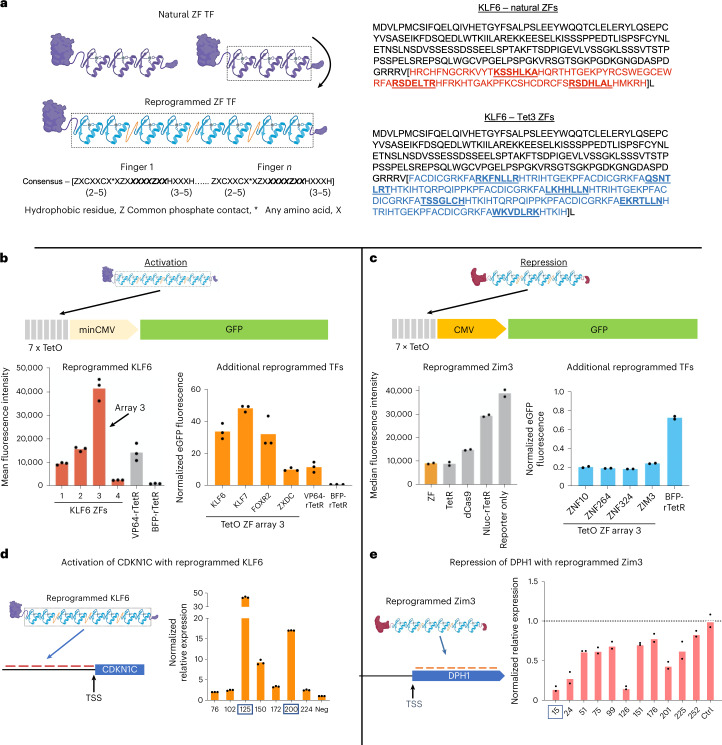
RTFs. **a**, Left, the ZFs of KLF6 are seamlessly replaced by designed ZFs. The consensus ZF motif, listed below, is used to guide the seamless replacement of parent ZFs. Right, sequence of the KLF6 TF and precise location of ZF replacement. **b**, A GFP reporter is activated with four ZF arrays designed to bind the tetO sequence. Array 3 is used to show that TFs other than KLF6 can also be reprogrammed to bind the tetO sequence and regulate the target. **c**, A GFP reporter is repressed by ZIM3 reprogrammed with tetO-binding array 3. This array can also be used to reprogram other repressing TFs in addition to ZIM3. **d**, Relative expression of *CDKN1C* by KLF6 reprogrammed with seven ZF arrays designed to bind sequences upstream of the TSS. **e**, Relative expression of *DPH1* repressed by ZIM3 reprogrammed with 11 ZF arrays designed to bind sequences downstream of the TSS. Source data

Because half of human TFs use ZFs to engage DNA, we reasoned that these endogenous ZF domains could be seamlessly replaced by designed ZFs without impacting the protein’s regulatory function (Fig. 5a). This approach presents the designed ZFs in the exact context in which ZFs would occur naturally in the parent protein. Such reprogrammed transcription factors (RTFs) present the effector domain in its natural context, maximize secondary interactions of the TF, avoid the use of foreign effector domains and enable research focused on the precise investigation of TF binding events. As potential therapeutics, RTFs present maximally native-like human proteins with correspondingly low immunogenicity risk. We chose the TF encoded by *KLF6* as our activation scaffold because we recently identified KLF6 as a potent activator when tethered to a reporter gene^44^. To test the activity of the KLF6 architecture, we replaced KLF6 ZFs with a series of ZF arrays designed to bind the tet operator sequence (tetO) (Supplementary Fig. 12). We replaced KLF6 ZFs with these designed ZF arrays and expressed RTFs in a stable HEK293T cell line containing a GFP reporter with a minimal promoter and seven tetO sites^44,45^ (Fig. 5b). Three of the four designs activated the reporter at a similar or greater level than rTetR-VP64. Next, we replaced the DBDs of three other activating TFs (genes *KLF7*, *FOXR2* and *ZXDC*)^44^ with Tet ZF array 3 (Fig. 5b). All of these RTFs activated the reporter as well or better than the rTetR-VP64 control. This included the FOXR2 RTF, where its natural forkhead DBD was replaced by the ZF array (Supplementary Fig. 13a).

We chose a TF encoded by *ZIM3* as our TF scaffold for repression, because the ZIM3 KRAB domain has proven a potent repressor as a SpCas9 fusion^46^. We replaced ZIM3 ZFs with the same series of tetO-binding ZF arrays tested with KLF6. We expressed these ZIM3 RTFs in a HEK293T cell line with a GFP reporter driven by a constitutive promoter. Three of the four ZF arrays repressed GFP expression relative to controls, with array 3 outperforming dCas9 (Fig. 5c and Supplementary Fig. 13b). To confirm that this RTF approach for repression was not restricted to the ZIM3 protein, we replaced the ZFs of three other KRAB-containing proteins (genes *ZNF10*, *ZNF264* and *ZNF324*) with ZF array 3. In all cases we observed similar levels of repression (Fig. 5c). Interestingly, despite the fact that the KOX1 KRAB domain (ZNF10) provides less repression potential than the ZIM3 KRAB domain when expressed as an isolated spCas9 fusion domain^46^, its activity was similar when expressed here as RTFs, suggesting that the presentation context can have a large impact on the potency of these domains.

Moving beyond reporter-based assays, we next designed RTFs to regulate genes from their natural loci in the human genome. We applied the ZIM3 architecture to repress three endogenous genes (*DPH1*, *RAB1A* and *UBE4A*). For each target gene we designed three arrays that bind at the transcriptional start site (1) and both the forward (2) and reverse sequence (3) that corresponds to a guide RNA target previously identified as a potent repressor of these genes by CRISPR interference. To maximize the likelihood of function, we designed these as eight-finger proteins and maintained the base-skipping linker between each designed ZF pair (Supplementary Fig. 15). HEK293T cells were transfected with RTFs and expression levels assayed by quantitative PCR with reverse transcription (RT–qPCR). While eight of the nine RTFs repressed the target gene, only two did so by >50% (Supplementary Fig. 15b). However, considering the extreme size difference between Cas9 and ZFs, it is possible that these functional positions for Cas9 are not optimal for ZFs. Therefore, we designed 11 ZF arrays to bind sequences across a 252-bp region downstream of the transcription start site (TSS) for *DPH1*. Again, we expressed these arrays in the ZIM3 scaffold and assayed *DPH1* expression. All arrays repressed *DPH1* relative to controls but two arrays, 15 and 126, repressed *DPH1* by >80% (Fig. 5e). Finally, to activate an endogenous target we took a similar approach and reprogrammed KLF6 with a series of arrays designed to canvass a 150-bp region upstream of the TSS in the *CDKN1C* promoter. All seven RTFs increased the expression of CDKN1C (in three of the seven by 9–43-fold) (Fig. 5d).

### Specificity and genome-wide regulatory activity of RTFs

**Fig. 6:**
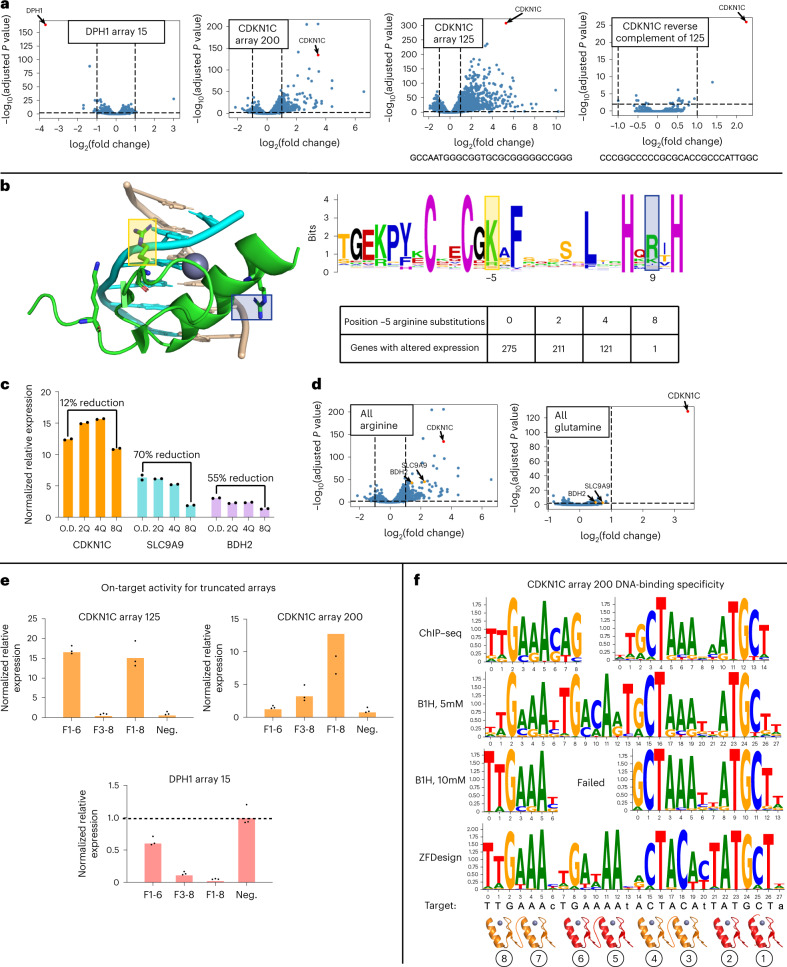
ZF specificity and genome-wide activity. **a**, Genome-wide RNA-seq results for CDKN1C arrays 125 and 200 and DPH1 array 15, and comparison with an array that binds the reverse complement of CDKN1C array 125. **b**, Left, structure of a ZF bound to DNA highlighting two potential phosphate contacts^51^. Right, the human ZF consensus with phosphate-contacting positions highlighted in yellow (−5) and blue (9)^49^. **c**, qPCR comparison for activation of the on-target CDKN1C gene as well as two off-target sequences with CDKN1C array 200 with between none and eight modifications at phosphate-contacting position −5. **d**, RNA-seq results for CDKN1C array 200 with arginines or glutamines at the −5 position of each ZF. **e**, On-target qPCR results for arrays with the N-terminal (F3–8) or C-terminal (F1–6) ZF pairs removed compared to an empty vector negative control (neg.). **f**, Specificity of CDKN1C array 200 array with glutamine at position −5 as determined by ChIP–seq, B1H selection at low (5 mM) and high (10 mM) stringency and specificity as predicted by ZFDesign. B1H specificity is a concatenation of the specificities determined for each of the two-finger pairs. ChIP–seq peaks contained two independent motifs, suggesting that the base-skipping linker allows modular, independent binding. Source data

ZFDesign enables the reprogramming of TFs for either activation or repression. To test the precision of regulation we used RNA sequencing (RNA-seq) to quantify RTF on- and off-target regulation. We focused on the two most potent KLF6 RTF activators for *CDKN1C* (arrays 125 and 200) and the most potent ZIM3 RTF repressor of *DPH1* (array 15) (Fig. 5d,e). In all cases the target gene was either the most—or one of the most—significantly regulated genes, but off-target activity ranged from seven (DPH1, array 15), to 268 (CDKN1C, array 200) and to 1,173 (CDKN1C, array 125) misregulated genes (Fig. 6a). Because KLF6 and ZIM3 are human TFs, we tested whether off-target activity is due to secondary interactions of the TF rather than ZF arrays. RNA-seq was carried out for CDKN1C arrays 125 and 200 using VP64 as the activation domain in place of KLF6, as well as KLF6 without any ZFs. These data suggest that off-target activity is primarily dictated by ZF arrays, because the KLF6 and VP64-ZFs had similar off-targets and KLF6 without ZFs resulted in only four genes with altered expression (Supplementary Fig. 16).

The specificity of ZF arrays can be impacted by both target content and affinity. As noted, G-rich binding tends to be more promiscuous. Consistent with this observation, the CDKN1C target with the lowest G content (no. 200; Supplementary Fig. 17) also led to the fewest off-target events while target 125 led to the most. To further test the influence of G-rich binding, we designed an array to target the reverse complement of the 125 sequence, which is necessarily a C-rich sequence. This approach reduced off-target activity to just one off-target gene (Fig. 6a, right). In addition to minimization of target G content, ZF specificity can be improved by reduction in the nonspecific affinity provided by contacts made between each ZF and phosphate backbone^47,48^ (Fig. 6b). This puts more pressure on the base-specifying interactions of each helix to provide the binding affinity necessary for function. We created mutant versions of CDKN1C array 200 that replace two, four or eight phosphate-contacting arginines at position −5 of the ZF scaffold with glutamines. Position −5 is dominated by basic residues in an analysis of human ZFs^49^ (Fig. 6b). We first compared the impact of these mutations by qPCR at both the target locus and two off-target loci found to be upregulated by RNA-seq (Fig. 6c). The expression of these off-target genes was reduced by up to 70 or 55%, respectively, as we increased the number of phosphate-contacting modifications. On-target activity, on the other hand, was reduced by only 12%. RNA-seq demonstrated that the number of off-targets was decreased as the number of modifications was increased, and that only *CDKN1C* was upregulated with the full eight arginine-to-glutamine modifications, thus providing single-target regulation. Interestingly, taking the same approach with *DPH1*-repressing array 15 resulted in a large reduction in on-target activity (Supplementary Fig. 18). It is notable, however, that the unmodified version of the array produced only six misregulated genes initially. This might suggest that DPH1 array 15 could already be at a low-affinity regime that cannot be lowered while preserving on-target activity. Together these data suggest that affinity and genome-wide specificity are tightly linked.

For the arrays used to regulate endogenous targets we used four pairs of ZFs to provide a greater opportunity for function, but it is not clear whether eight ZFs are necessary. A six-finger array provides up to 18 bp of specificity that would give single-target resolution in the human genome. To test whether eight fingers are necessary in each array, we tested the on-target activity of CDKN1C arrays 125 and 200 and DPH1 array 15 with either the N- or C-terminal ZF pairs removed (Fig. 6e). In the case of CDKN1C array 200, removal of either terminal pair led to a large decrease in activity. Conversely, removal of the C-terminal pair for CDKN1C array 125 or of the N-terminal pair for DPH1 array 15 had very little impact on activity. Together, these data suggest that in some (CDKN1C array 125 and DPH1 array 15), but not all (CDKN1C array 200), a six-finger version of proteins can provide similar activity but eight-finger proteins may capture activity for more challenging targets.

Finally, to characterize the DNA-binding specificity of the ZF arrays, we first performed independent B1H binding selections for each pair within CDKN1C arrays 125 and 200 and DPH1 array 15 (Supplementary Fig. 19). Because the flexible linker between pairs in these arrays will lead to modular and independent binding (Fig. 2d), these screens were used to confirm that each pair can bind their subtarget independent of the full array. We screened these pairs with an 8-bp library at two stringencies and found that specificities were in general agreement with their designed targets (Fig. 6f and Supplementary Fig. 20). Next, we tested the genome-wide specificity of the full eight-finger arrays by chromatin immunoprecipitation sequencing (ChIP–seq). Here we found that, despite the proper specificity provided by each pair, subsets of ZFs appear to drive binding genome wide. This is probably due to the modular design of our arrays that allows each pair to bind independently, and therefore the highest-affinity arrays will guide most ChIP–seq peaks. We used this design approach for simplicity in this proof-of-concept stage but, in future work, ZF arrays employing conventional linkers that do not skip bases should exhibit reduced off-target binding because in those, much less independent binding of ZF pairs will occur. It is furthermore worth noting that ZFDesign can be used to approximate the specificity of designed arrays with relatively high accuracy (Methods), and with much better accuracy than approaches trained specifically to predict ZF specificity (Fig. 6f and Supplementary Fig. 20). This suggests that ZFDesign can potentially be used to identify ZF arrays that are more likely to show specific binding before experimental validation.

## Discussion

In this study we present ZFDesign, a hierarchical attention-based artificial intelligence model trained on comprehensive screens of ZF–DNA interactions that consider the influence of multiple adjacent finger environments. ZFDesign captures these influences to provide a general design model for ZF arrays. By contrast, previous efforts produced incomplete collections of ZF modules that often fail out of context and produce low on-target activity. Conversely, our model consistently produced ZF arrays across a wide range of targets at high efficacy as nucleases, repressors and activators. Thus, ZFDesign represents an important advance because the design of ZFs for any given target is now available at the push of a button and is open to the academic community for study of a myriad of academic and therapeutic applications, with the advantages of small size and low immunogenicity.

The modularity of our arrays led to a large amount of off-target binding, despite the fact that each pair within the array presents a specificity profile that would accommodate the target sequence. Off-target binding is probably driven by the highest-affinity pairs in the array that will bias the binding towards their targets, because our linker arrangement will encourage this modularity. These data are juxtaposed by the RNA-seq results that demonstrate very low off-target activity. Together these data demonstrate that, much like natural TFs that have thousands of binding sites across the genome, the position of binding is critical for regulation and not all binding events have the potential to modify gene expression. This disparity between binding and activity has also been seen with SpCas9, where ChIP–seq can reveal thousands of off-target binding events dominated by seed specificity despite minimal evidence of off-target catalytic activity^50^.

Design limitations remain because our model was trained on two-finger selections that sampled <5% of the possible 6-bp targets, and single-finger selections did not sample T at the overlap positions. Therefore, we are most confident in those domains that bind A, C or G at the overlap. Further, we are just beginning to understand specificity and off-target activity because the model was first built to capture compatibility. As more arrays are screened and specificities generated, the model will be continuously improved to capture the rules of specific binding. Nevertheless, we have shown that the modification of nonspecific affinity can improve specificity for ZF designs, even at single-target resolution. This suggests that ZFDesign arrays do prefer the on-target sequence, but modularity and our understanding of their discrimination against alternative sequences is limited. Nevertheless, a ZFDesign user might choose high-activity designs that maintain natural phosphate contacts or high-specificity designs by starting with the modified domains.

Finally, we present a generalizable design method that allows for the seamless replacement of a TF natural DNA-binding domain to direct the TF to any target of interest. These RTFs can produce activation and repression activities similar to CRISPR-based tools, establishing these proteins as attractive therapeutics comprising solely human components. In addition, these tools allow us to better probe TF function because they more accurately mimic natural TFs. We believe these tools will open exciting opportunities in systems and synthetic biology for the investigation and modification of gene regulation on a genome-wide scale.

## Methods

### Experimental

#### Library builds

##### Primary ZF libraries

All primary ZF libraries were built actions^35,52^. To provide templates for PCR, gBlocks were ordered from IDT that coded for the finger 0 and 1 domains of each library. The critical difference between libraries is the different environment presented at the interface between domain 1 and library domain 2. gBlock provides the template that will differentiate each library. These libraries were chosen to present side chains at the interface with a range of biochemical properties (basic, acidic, polar, aromatic and hydrophobic interactions) and thereby comprehensively capture a broad range of potential adjacent finger influences. Next, an oligonucleotide was designed with degeneracy (NNS) at the codon positions corresponding to the six critical residue positions of the ZF domain 2 alpha-helix. This oligo was used to build all libraries with only the gBlock template differentiating one library from the next. For each library, PCR reactions were run in 96-well-plate format and pooled. PCR products were digested and cloned into the B1H expression vector. Library ligations were electroporated into 15 aliquots of electrocompetent cells and recovered in 1 l of super optimal broth with catabolite repression (SOC) media for expansion. To select for transformants, carbenicillin was then added to the culture 1 h post electroporation and grown to mid-log. Library DNA was then recovered by Qiagen maxiprep. Library sizes ranged 1–3 × 10^9^. This approach has been consistently shown to produce libraries with diversity approximating random^52^.

##### Two-finger libraries

Second-round selections were used to select compatible pairs from preselected ZF pools generated in the primary ZF library selections. We pooled recovered plasmid DNA from our primary single-finger screens on a binding site basis, resulting in a pool of diverse helices (termed ‘round 2 pools’) with broad compatibility for each of the 64 different binding sites. These round 2 pools were used as a PCR template to create either ‘domain 1’ or ‘domain 2’ amplicons using the Expand High Fidelity PCR system (Roche), and 15 cycles of PCR and two-finger pools were then assembled by overlapping PCR using the domain 1 and 2 amplicon as the template for a subsequent round of PCR. These two-finger pools were purified, digested and cloned into our two-finger expression vector. One hundred nanograms of the ligation was electroporated into USO-ω cells, recovered in SOC for 1 h and titered to measure library size. Based on these cell counts, 5 × 10^6^ cells were plated on 15-cm rich medium agar plates with carbenicillin, grown at 30 °C for 12–14 h, harvested by scraping and miniprepped to obtain the final two-finger libraries.

##### Eight-base-pair library

The specificity of two-finger modules was determined using a modified B1H reporter (conceptually similar to a 10-bp library used to characterize ZF–homeodomain fusions^53^; Supplementary Fig. 19). The library was built as previously described^54^, with a random barcode and library window placed between the Not1 and EcoRI restriction sites of the B1H reporter vector.

#### ZF selection

##### Primary ZF libraries

Libraries were built in a vector that expresses ZFs as a fusion to the omega subunit of the bacterial polymerase that acts as the activation domain in this system. Each binding site reporter vector was built by placing the binding site of interest 10 bp upstream of the −35 box of the promoter that drives HIS3 and GFP expression in the previously described GHUC vector. For each selection, the Δ*rpoZ* selection strain was transformed with the ZF library and the appropriate reporter plasmid by electroporation. Cells were expanded in 10 ml of SOC for 1 h at 37 °C with rotation, recovered and resuspended in minimal medium supplemented with histidine and grown with rotation for an additional 1 h at 37 °C. Finally, cells were washed in minimal medium lacking histidine, recovered in 1 ml of this medium and 20 μl was plated in serial dilution on rich plates containing kanamycin and carbenicillin to quantify double transformants. This plate was grown at 37 °C overnight while the remaining 980 μl of transformed cells was stored at 4 °C. Once grown, serial dilutions were counted and a volume containing a minimum of 5 × 10^8^ cells were taken from the stored transformants and plated on selective medium containing 2 mM 3-AT, a competitive inhibitor of HIS3. Plates were incubated for 36–48 h at 37 °C. Colonies were counted, cells pooled and DNA harvested and amplified for Illumina sequencing.

#### Compatible two-finger module selection

Round 2 libraries were cotransformed with the matching reporter vector in USO-ω cells, recovered and titered for cell count, then 1 × 10^6^ cells were added in triplicate to a 96-well deep-well plate containing a sterile bead for efficient agitation. Selections were performed in 1 ml of NM + Ura/-His supplemented with 100 μg ml^–1^ carbenicillin, 50 μg ml^–1^ kanamycin, 1 μM isopropyl-β-d-thiogalactopyranoside and 5 mM 3-AT. These were grown at 37 °C in a plate shaker for 18, 24 or 40 h and harvested on reaching visible turbidity (typically optical density >0.6). Triplicates were pooled, miniprepped and deep sequenced on an Illumina NextSeq 500.

##### Eight-bp library selections

Four-finger proteins were expressed as C-terminal fusions to omega (*rpoZ*) that functions as the activation domain in the B1H assay (Supplementary Fig. 19). The first two fingers are fixed and their target sequence is placed downstream of the random region of the library. In this way, the fixed fingers function as anchors that site the test pair of fingers in an optimal position to interact with DNA sequences in the library window. An upstream 4-bp barcode is used to filter the rare cells that escape selective pressure, to minimize their influence on downstream analysis. Each ZF expression vector was cotransformed with the 8-bp library vector, and 6.5 × 10^6^ cells were plated on selective medium supplemented with either 5 mM 3-AT (low stringency) or 10 mM 3-AT (high stringency). Plates were incubated for 24–36 h at 37 °C. Surviving colonies were pooled, DNA recovered and sequenced by Illumina.

#### U20S GFP disruption assay

Zinc finger nuclease activity was assessed by measuring disruption of an integrated, constitutively expressed eGFP reporter in a clonal U2OS cell line previously described^41^. Cells were cultured in DMEM supplemented with 10% FBS, 2 mM GlutaMax (Life Technologies), 1% penicillin/streptomycin, 1% MEM nonessential amino acids (Life Technologies), 2 mM sodium pyruvate and 400 μg ml^–1^ G418. One microgram of each ZFN monomer plasmid DNA and 200 ng of ptdTomato-N1 plasmid DNA were transfected in duplicate into 5 × 10^5^ cells using a Lonza Nucleofector 2b Device (Kit V, Program X-001). In each assay 2 μg of parental empty vector (a modified derivative of the JDS71 vector from Addgene) and 200 ng of ptdTomato-N1 were used as a negative control, and 2 μg of a dual-spCas9-guide-expressing vector (modified Addgene plasmid no. 41815) and 200 ng of ptdTomato-N1 as a positive control, in each experiment. Cells were grown in six-well dishes for 3 days post transfection, harvested, maintained on ice and analyzed for expression of eGFP and tdTomato on a Sony SH800 cell sorter. To restrict the analysis to cells that probably received both ZFN monomer plasmids, populations were first gated on the top 15–25% tdTomato^+^ cells then analyzed for loss of eGFP expression (Supplementary Fig. 11).

#### Next-generation sequencing and preparation

Following selection from ≥5 × 10^8^ library variants, surviving colonies were pooled, miniprepped and DNA barcoded for sequencing on an Illumina NextSeq 500. Two microliters of pooled plasmid DNA was used as a template for barcoding in a 25-μl reaction with Taq Polymerase (NEB), with the following cycling parameters: 95 °C for 5 min, 20 cycles of (95 °C, 20 s; 52 °C, 30 s; 68 °C, 30 s) and 68 °C for 10 min, and was then held at 4 C. Each 5-μl reaction was visualized on 1% agarose gel to confirm apparent equal amplification, and reactions were pooled in equal volumes. These were run out on 1% agarose gel, gel purified and submitted to the NYU Genome Technology Center for sequencing on a NextSeq 500.

#### Live cell imaging of ZF–GFP fusion

We designed ZFs to bind the sequence 5′-CGCCCAGCTGGGGGCGGGGGA-3′, a sequence that is repeated 111 times at the *Brf1* locus on chromosome 14 (hg38 chr14: 105229626–105240946). The coding sequence for the designed ZF array was ordered from IDT (gBlock) A SV40 NLS was added to the C termini by PCR. Next, we added GFP as an N-terminal fusion to the ZFs using the NT-GFP Fusion TOPO TA Expression Kit (Invitrogen). Successful cloning into the expression vector was confirmed by Sanger sequencing.

The GFP–ZF fusion expression vector was transfected into 293 T cells and grown on 0.01% Poly-l-lysine-coated 35-mm MatTek dishes using X-treme-GENE 9 DNA transfection reagent (Sigma-Aldrich). Transfected cells were Hoechst stained the following day and then imaged. A titration experiment was conducted to explore optimal plasmid concentration. Clear foci were visible at a range of concentrations, but 333 ng of plasmid yielded the optimal balance of transfection efficiency and signal-to-noise ratio.

#### Chemically mediated transfection of HEK293T cells

##### Mirus transfection

For KLF6-based RTFs, 18–24 h before transfection, HEK293T cells were passaged and 7.5 × 10^5^ cells added to 2.5 ml of medium in a six-well dish. Cells were transfected with 2 μg of plasmid DNA at a 4:1 ratio of DNA:*Trans*IT-LT1 transfection reagent (Mirus) according to the manufacturer’s instructions.

##### Effectene transfection

Transfection of Zim3-based RTFs were performed using a modified Effectene reagent (Qiagen) protocol as previously described^55^, because we found the improved transfection efficiency with this protocol was necessary to achieve high levels of bulk repression. Per transfection, 0.4 µg of DNA was used in 100 µl of of EC buffer at a DNA:enhancer ratio of 1:8 (3.2 µl) and DNA:Effectene ratio of 1:15 (6 µl). The resulting transfection complexes were added to each well in a six-well plate freshly seeded with 5 × 10^5^ HEK293 T cells in 2 ml of medium. All transfections were performed in triplicate.

#### Cell culture and RT–qPCR analysis of repressors and activators

HEK293T cells were transfected with ZF activators and repressors, and target transcript levels measured by RT–qPCR as follows. Cells were cultured in DMEM supplemented with 10% FBS, 2 mM GlutaMax (Life Technologies), 1% penicillin/streptomycin, 1% MEM nonessential amino acids (Life Technologies) and 2 mM sodium pyruvate. Medium was changed 2 days post transfection and cells harvested for RT–qPCR 3 days post transfection. Cells were washed once with sterile PBS, 350 μl of Buffer RLT Plus (Qiagen) containing 1% β-mercaptoethanol was added and samples were either stored at −80 °C or processed immediately using the RNeasy Plus Mini Kit (Qiagen) according to the manufacturer’s instructions.

One microgram of pure RNA was reverse transcribed using the SuperScript IV First-Strand Synthesis System (Invitrogen) according to the manufacturer’s instructions. Random hexamers were used as primers. qPCR reactions were established in technical duplicate or triplicate using the equivalent of 25 or 50 ng of reverse-transcribed RNA per reaction and the KAPA SYBR FAST qPCR Master Mix (2X) (Roche).

RT–qPCR was performed on a LightCycler 480 Instrument II (Roche) using the cycling program recommended for KAPA SYBR FAST reagent with the LightCycler 480 (annealing temperature 60 °C). Cycle threshold values were calculated using the on-board Absolute Quantification/2^nd^ Derivative Max analysis option. Input was first normalized using the housekeeping gene *RPS18*, and fold change in expression for a given gene of interest was calculated relative to the appropriate negative control. A table of RT–qPCR primers used in this study can be found in Supplementary Data.

#### Production of GFP-tagged TF expressing HEK293 Flp-In-TRex cell lines

Cell lines were produced using an engineered Flp-In vector backbone with an N-terminal eGFP tag. Parental cells were transfected with each specific ZF vector separately (FuGENE HD Transfection Reagent, Promega) in six-well plates and transferred to hygromycin selection medium after 48 h. All colonies from the same plate were pooled and used for further experiments. Cells were treated with doxycycline (100 ng ml^–1^) 24 h before crosslinking, and GFP expression was confirmed by fluorescent microscopy.

#### ChIP–seq

Chromatin immunoprecipitation was performed as previously described^56^. In brief, ∼2 × 10^7^ HEK293 Flp-In-TRex cells were crosslinked for 10 min in 2.7% formaldehyde followed by 10 min of quenching. Lysates were sonicated to a DNA fragment length of 200–300 bp using a Bioruptor (Diagenode). GFP-tagged transcription factors were immunoprecipitated with a polyclonal anti-GFP antibody (no. ab290, Abcam) and Dynabeads Protein G (Invitrogen). Subsequently, crosslinks were reversed at 65 °C overnight and bound DNA fragments purified (QIAquick PCR Purification Kit, Qiagen). Each construct was tested in duplicate.

### Computational analysis

#### Sequence recovery and filtering

All paired-end Illumina reads were demultiplexed and trimmed into 21-mers with in-house Unix scripts based on EMBOSS 6.6.0. Trimmed DNA sequences are translated, and amino acid sequences considered if they had a least two read counts and were coded by at least two different DNAs. Invariant leucine at helix position +4 is excluded.

#### Clustering and filtering selection

For each selection, helix sequences were clustered using MUSI software^34^. Each sequence was assigned to the cluster associated with the PWM for which it was assigned the highest responsibility. For each cluster generated, Shannon entropy value was calculated for each helix residue based on the PWM for that cluster. If a selection lacked a cluster with at least one position with entropy of two or less, that selection was filtered out for downstream analysis.

#### Computation of similarity between selections by Hamming distance

To compare helices from two selections, A and B, pairwise normalized Hamming distances were computed between the two sets of filtered sequences based on the number of identical amino acids. The minimum normalized Hamming distance was then computed from each helix in selection A to each helix in selection B, as well as from each helix in selection B to each helix in selection A. The overall distance between the two selections was computed as the mean of these distances.

#### Molecular dynamic simulations

Similar to our previous studies^35,57^, the PDB file 1AAY^51^ was used as template and DNA was elongated by 2 bp at each end using X3DNA to avoid the melting end effect so that the binding of ZFs would not be affected. DNA and protein sequences were mutated using Chimera (www.cgl.ucsf.edu/chimera/) for each library and test case, and the protonated states were determined by WHAT IF (swift.cmbi.umcn.nl/whatif/). The prepared structures were then solvated into a TIP3P water box with a 15-Å buffer of water extending from the protein–DNA complex in each direction, with the addition of sodium ions to ensure overall charge neutrality. The FF99 Barcelona forcefield was used for the protein–DNA complex and zinc amber forcefield for zinc ions. The particle mesh Ewald method was used for electrostatics calculations. The SHAKE algorithm was used to constrain hydrogen-containing bond length, which allowed a 2-fs time step for MD simulation. The nonbonded cutoff was set to 12.0 Å. The systems were energy minimized using a combination of steepest descent and conjugate gradient methods. The systems were then thermalized and equilibrated for 3 ns using a multistage protocol. The first step was a 1.5-ns gradual heating from 100 to 300 K followed by 1.5 ns of density equilibration, both at 1 fs step length. A Berendsen thermostat and barostat were used for both temperature and pressure regulation for a further 6-ns equilibration at 2-fs step length, with gradually reduced positional constraints at 300 K. The systems were built with tleap and simulations conducted with graphics processing unit-accelerated Amber18 (ref. ^58^). For each system, three 500-ns trajectories were simulated. Hydrogen bond analysis was performed using BioPython. We considered as hydrogen bonds any contacts <3.5 Å between atoms O6 and N7 (for a guanine), between atoms NH1 and NH2 (for an arginine) or between atoms ND2 and OD1 (for an asparagine). Bifurcated hydrogen bonds between a guanine and arginine were identified when pairs 06–NH1/2 and N7–NH1/2 were found, allowing the tautomeric bifurcated hydrogen bond.

#### Calculation of entropy of binding for core helices across libraries

To quantify the promiscuity of helices targeting each nucleotide 3-mer, Shannon entropy was computed. For each nucleotide 3-mer, a position frequency matrix of nucleotide sequences targeted by every set of core residues (−1, 2, 3, 6) was computed. Entropy was calculated in a position-wise fashion and then summed to obtain an overall metric for specificity.

#### Neural network architecture

We developed a hierarchical neural network architecture that mimics the B1H experimental setup and captures the modularity of ZF proteins. This architecture is composed of three modules (Fig. 3). The first two modules are trained to generate helices that bind to a particular nucleotide 4-mer that includes the target 3-mer and the overlap base. The residue embeddings from these modules are concatenated and used as input to a third module that is designed to learn compatibility between the helices in a pair (Fig. 3a). The first module generates residue embeddings for the first helix in a pair based on the last four bases in a target 7-mer, and the second module generates residue embeddings for the second helix based on the first four bases in a target 7-mer (Fig. 3b). The full model is trained to predict all core residues in two helices given a nucleotide 7-mer.

The architecture of the first two modules is largely based on the Transformer model^59^. An encoder generates a high-dimensional representation for each base in a nucleotide 4-mer. A decoder then generates predictions for each core residue in a ZF helix using self-attention and attention layers that relate nucleotide bases to helix residues. While the decoder in a conventional Transformer strictly generates sequences from left to right^59^, the decoders in this model use bidirectional information. A portion of the residues in a helix is masked and the decoder outputs amino acid predictions at these positions. The third module consists of repeating self-attention and feedforward layers that allow the model to update residue embeddings based on interhelix compatibility (Fig. 3b).

Variants of the first module with different numbers of attention heads and embedding dimensions were trained and evaluated on the initial task of predicting residues in a single helix (Supplementary Table 1). In the final model, all attention layers were repeated three times and each attention layer had four heads. The model-embedding dimension (*d*_model_) was set to 128. The value- and key-embedding dimensions for computation of scaled dot-product attention (*d*_v_ and *d*_k_, respectively) were both set to 256. The hidden dimension in the feedforward layers was set to 128. For regularization, dropout layers were included after every feedforward and attention layer with a dropout percentage of 0.3.

#### Training datasets

The models were trained and evaluated on data derived from B1H selections. B1H screening data were filtered using a previously described approach in which helices were evaluated based on the diversity of encoding nucleotide sequences found in the screen^35,52,60^. Shannon entropy for each helix (or helix pair) was calculated based on the number of reads associated with each possible encoding nucleotide sequence. Helices were filtered based on previously defined thresholds^35^. Specifically, helices with fewer than ten reads or Shannon entropy <0.07 were removed.

Modules 1 and 2 were pretrained using data from single-helix B1H selections performed against nucleotide 4-mers. The data included selections performed with 11 libraries against 192 different nucleotide 4-mers. In total, the dataset included 2,071,764 data points. For initial training and hyperparameter tuning, data points were split into training, test and validation datasets at proportions of 80, 10 and 10%, respectively, by 4-mer sequence. For pretraining, data were instead split by helix sequence.

The full model was trained using data from helix-pair B1H selections performed against nucleotide 7-mers. An initial dataset of selections against 189 seven-mers was split into training and validation datasets at proportions of 90 and 10%, respectively. This dataset contained a total of 327,792 data points. To ensure that the validation set was sufficiently different from the training dataset, a graph was generated where nucleotide 7-mers were represented as nodes, and edges connected 7-mers within two base substitutions from each other. While most of the nodes formed a single connected component, there were separate components included in the validation dataset (Supplementary Fig. 22a). Nodes with the lowest degree in the graph, and their neighbors, were then added to the validation dataset. Most of the sequences in the validation dataset were consequently at least three mutations away from any sequence in the training dataset (Supplementary Fig. 22b). A separate set of 15 selections, filtered to ensure at least 100 unique helix pairs, was used as an independent test set for model evaluation.

#### Model training

In both training steps, a nucleotide target and a sequence of partially masked core residues from either a single ZF or helix pair were provided to the model; 50% of core residues were masked and cross-entropy loss was evaluated based on output probabilities. Training was done carried out an Adam optimizer with a learning rate of 1 × 10^–4^, and a minibatch size of 128 was used. Early stopping was carried out based on validation loss. Pretraining modules 1 and 2 required, at most, 1.3 million iterations; training the full model required, at most, 3.4 million iterations. When training the full model, the parameters for modules 1 and 2 were either randomly initialized, transferred from the pretraining step, or transferred and from the pretraining step and frozen (Supplementary Fig. 23).

#### De novo design of ZF–helix pairs

When predicting zinc finger residues, the model makes use of context provided by known residues. Helix sequences are generated incrementally where the network is run once for each missing residue. At each iteration, a single residue is added to increase the sequence context. For a pair of helices, there are about 4.1 × 10^15^ possible sequences and about 4.8 × 10^8^ orders in which each sequence can be generated. Enumeration of all possibilities to find the sequence with the highest likelihood is thus computationally intractable.

To generate sequences we adapted the A* search algorithm, as done previously^38,61^. This approach involves iteratively filling in masked residues while maintaining a priority queue of partially masked sequences. At every iteration, the top partially masked sequence is taken from the priority queue and passed through the network. All possible labels for every masked residue are evaluated. Any label with probability >0.05 is accepted and that label added to a copy of the input sequence before being pushed onto the priority queue. This is repeated until a set number of sequences areis completely generated. The following equation is used to assign a priority to each partially masked sequence:

$$p_j = \mathop {\sum}\limits_{i = 1}^j {{{{\mathrm{log}}}}\left( {p_i} \right)} + \mathop {\sum}\limits_j^{12} {{{{\mathrm{log}}}}\left( {p^ \ast } \right)}$$

This heuristic approximates the maximum expected probability of a sequence that would be attained by predicting the remaining residues. $p_i$ denotes the probability assigned to the prediction made at iteration *i*, and *j* denotes the number of predicted residues. $p^*$ denotes the expected maximum probability that would be assigned by the network to later predictions. This parameter can be tuned to move the search closer to a greedy or breadth-first search. This parameter was set to 0.1 whenever A* was performed in this work.

We also implemented an alternative biased sampling approach using temperature-adjusted distributions, as done previously^39^. This approach generally resulted in higher likelihood sequences (Supplementary Figs. 22 and 23). At every iteration, the probability of predicting an amino acid *i* at position *j* is the following:

$$p\left( {x_{i,j}|n,x_{\left( {k,m} \right) \in S}} \right)^{\left( T \right)} = \frac{{p\left( {x_{i,j}|n,\,x_{(k,m) \in S}} \right)^{\frac{1}{T}}}}{{\mathop {\sum}\nolimits_{a = 1}^{20} {\mathop {\sum}\nolimits_{b = 1}^{12} {p\left( {x_{a,b}{{{\mathrm{|}}}}n,\,x_{\left( {k,m} \right) \in S}} \right)^{\frac{1}{T}}} } }}$$

where *n* denotes the input nucleotide sequence and *S* the set of pairs of amino acids and positions already predicted. *T* is an adjustable parameter that controls the bias of the distribution; this parameter was set to 0.6 when this method was used. In total, 10^5^ ZF pairs were sampled and the maximum-likelihood pair when performing de novo design.

#### Comparison with ZFPred

To generate distributions over helix sequences using ZFPred^35^, 10^6^ helix sequences were randomly sampled. The binding specificities of these helices were predicted using ZFPred. Sequence distributions for a particular nucleotide sequence were then generated by normalization of the predicted scores of the sampled helices for that nucleotide sequence. Predictions for 3-mers were concatenated to generate predictions for 6-mer sequences.

#### RNA-seq analysis

RNA-seq library preps were constructed with the Illumina TruSeq Stranded mRNA Library Prep kit (no. 20020595) using 500–1,000 ng of total RNA as input, amplified by 10–12 cycles of PCR and sequenced paired-end 50 cycles on Illumina sequencers with 2% PhiX spike-in. Between 25 and 30 million reads were obtained for each sample. Paired-end reads were aligned to hg38 using STAR aligner^62^. Read counts were computed using FeatureCounts, and differential expression analysis was subsequently performed using DESeq2 (ref. ^63^).

#### Statistical analysis

Two-sided Wilcoxon rank-sum tests were performed using the SciPy python library. Boxplot centerlines show medians, box limits show upper and lower quartiles, whiskers are 1.5× interquartile range and points show outliers.

#### Specificity estimation with ZFDesign

To estimate the specificity of a helix pair, the pseudolog-likelihood of the pair with every 7-mer was calculated using ZFDesign. This was done by iteratively masking each residue and computing its log-likelihood with the remaining amino acids provided as context. Pseudolog-likelihood is the sum of these log-likelihoods and has previously been shown as an effective way to score sequences generated using masked language models^64^. The resulting pseud-log-likelihoods are then normalized to obtain a distribution over 7-mers for a particular helix pair.

#### ChIP–seq analysis

Paired-end reads were aligned to hg38 using bowtie2 (ref. ^65^). MACS2 was used to call peaks using input DNA as a control^66^. Motif finding was done using MEME–ChIP^67^.

#### B1H specificity analysis

For specificity analysis, B1H reads were filtered using three criteria. First, 8-mer sequences with fewer than ten reads in total were removed. Additionally, 8-mers recovered on only a single unique plasmid, based on the upstream barcode, were removed. Finally, if the entropy of the distribution of reads for a particular 8-mer across different plasmids was <0.1, reads corresponding to the most frequent plasmid were filtered out. This was done to ensure that cells that escaped selective pressure were not overrepresented. MUSI was used to cluster the set of 8-mers into two different motifs. The resulting read distribution was normalized using the distribution of reads in the sequenced background library.

### Reporting summary

Further information on research design is available in the Nature Portfolio Reporting Summary linked to this article.

## Online content

Any methods, additional references, Nature Portfolio reporting summaries, source data, extended data, supplementary information, acknowledgements, peer review information; details of author contributions and competing interests; and statements of data and code availability are available at 10.1038/s41587-022-01624-4.

## Supplementary information

Supplementary Information Supplementary Figs. 1–24 and MTA agreements.

Supplementary Data Data for generation of Supplementary figs.

Reporting Summary

## Data Availability

Access to the ZF selection data used to define and train the model is available for academic purposes through execution of a material transfer agreement. Please contact the NYU Langone Health Technology Opportunities team (innovationscontracts@nyulangone.org) or the corresponding authors for details (pm.kim@utoronto.ca and marcus.noyes@nyulangone.org). Source data are provided with this paper.

## Source data

Source Data Source Data Figs. 5 and 6: sequence information for oligonucleotides, proteins, DNA targets and ChIP–seq sequences used to support binding motifs and PCR primer list.

## Code availability

Access to ZFDesign and the underlying code is available for academic purposes through execution of a material transfer agreement. Please contact The Governing Council of the University of Toronto (innovations.partnerships@utoronto.ca) or the corresponding authors for details (pm.kim@utoronto.ca and marcus.noyes@nyulangone.org).
