# Supplementary material for: A universal deep-learning model for zinc finger design enables transcription factor reprogramming
Source: Nat Biotechnol. 2023 Jan 26;41(8):1117–29. doi: 10.1038/s41587-022-01624-4 (PMC10421740; doi:10.1038/s41587-022-01624-4)
Supplement: Supplementary file 1 — Supplementary Figs. 1–24 and MTA agreements. [file 41587_2022_1624_MOESM1_ESM.pdf]

nature biotechnology

Article https://doi.org/10.1038/s41587-022-01624-4

A universal deep-learning model for zinc finger design enables transcription factor reprogramming

In the format provided by the authors and unedited

A universal deep-learning model for zinc finger design enables transcription factor reprogramming

Supplementary Figures:

Table of contents

Figure S1 Comparison of DNA-binding domain size and relation to DNA...………**page 2**
Figure S2 Zinc finger interface and common selection strategies……………….**page 3,4**
Figure S3 List of libraries and interfaces tested……………..……..……..………..**page 5,6**
Figure S4 Global Hamming distance comparisons ………………………..…...…**page 7-9**
Figure S5 1-Hamming distance dot plot comparison of libraries by target .....**page 10-12**
Figure S6 Promiscuity of G-rich binding ……………...……………………….....…**page 13**
Figure S7 Performance of single-helix design modules……………...………...… **page 14**
Figure S8 Attention values comparing nucleotide to amino acid distances……..**page 15**
Figure S9 Predicted, real B1H, and concatenated single-helix B1H logos…..…**page 16**
Figure S10 Designed zinc finger nucleases…………………………..…………..…**page 17**
Figure S11 Cytometry plots of positive and negative controls for ZFN assay…..**page 18**
Figure S12 TetO-targeting ZF arrays for activation or repression ……………....**page 19**
Figure S13 RTF sequences with the Tet array#3 ZF…………………………..**page 20,21**
Figure S14 EGFP repression by ZIM3 RTFs……………………...……………...…**page 22**
Figure S15 Repression of endogenous genes with ZIM3 RTFs……...…………**page 23**
Figure S16 Global RNA-seq by CDKN1C-targeting ZF arrays……………...…..**page 24**
Figure S17 The influence of target G-content and nonspecific affinity………...…**page 25**
Figure S18 Phosphate-contacting substitution and DPH1 array #15…………….**page 26**
Figure S19 8-bp B1H binding site selections for 2-finger characterization…...…**page 27**
Figure S20 Specificity for CDKN1C array #125 and DPH1 array #15..…………**page 28**
Figure S21 Distribution of target sequences for training and validation data……**page 29**
Figure S22 The effect of pre-training on model performance……………………..…**page 30**
Figure S23 Impact of scale on A* or temperature dependent sampling……..…..**page 30**
Figure S24 Comparison of ChIP-seq peaks with RNA-seq……………………….**page 31**

Supplementary Figures:

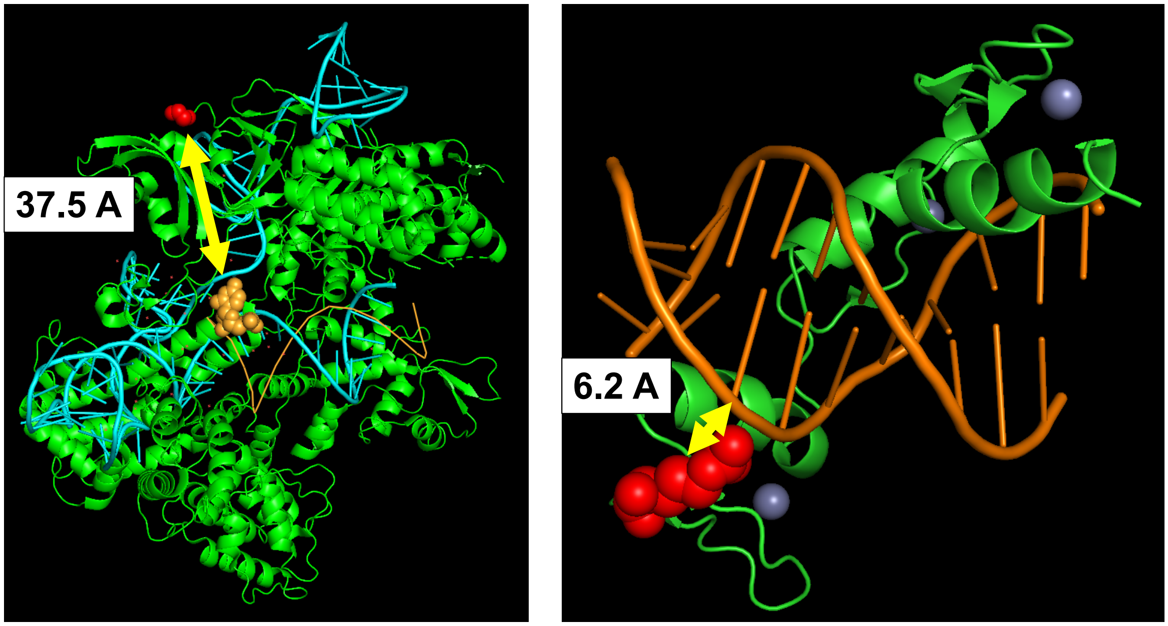

DNA-binding domain	Human TFs[1]	Monomeric size (aa)	Functional state	Total size (aa)	~Target length (bp)	aa's per base
Forkhead	49	102	Monomer or dimer	204	12	17
Basic Leucine Zipper	71	61	Dimer	122	10	12.2
Basic helix-loop-helix	111	54	Dimer	108	8	13.5
Homeodomain	222	60	Monomer or dimer	120	12	10
C_2H_2 zinc finger	760	28	Monomer (Requires arrays of multiple domains)	252 (The average human ZF-TF has 9 ZFs)	3-4bp per monomer	9.3
SpCas9	-	1368	Monomer	1368	23	59.4

Fig. S1. **Comparison of DNA-binding domain size and relation to DNA.** *Left,* X-ray crystal structure of spCas9 bound to DNA[2]. *Right*, Structure of zinc fingers bound to DNA[3]. Arrows indicate approximate distance between the C-terminus of the domain and the bound DNA.

Table shows the number of human transcription factors that use five common DNA-binding domains[1] and their comparative size. As many DNA-binding domains require dimerization, their monomeric and multimeric sizes are listed. A comparison of the multimeric size and the domain's common target length allows a calculation of amino acids required per base specified.

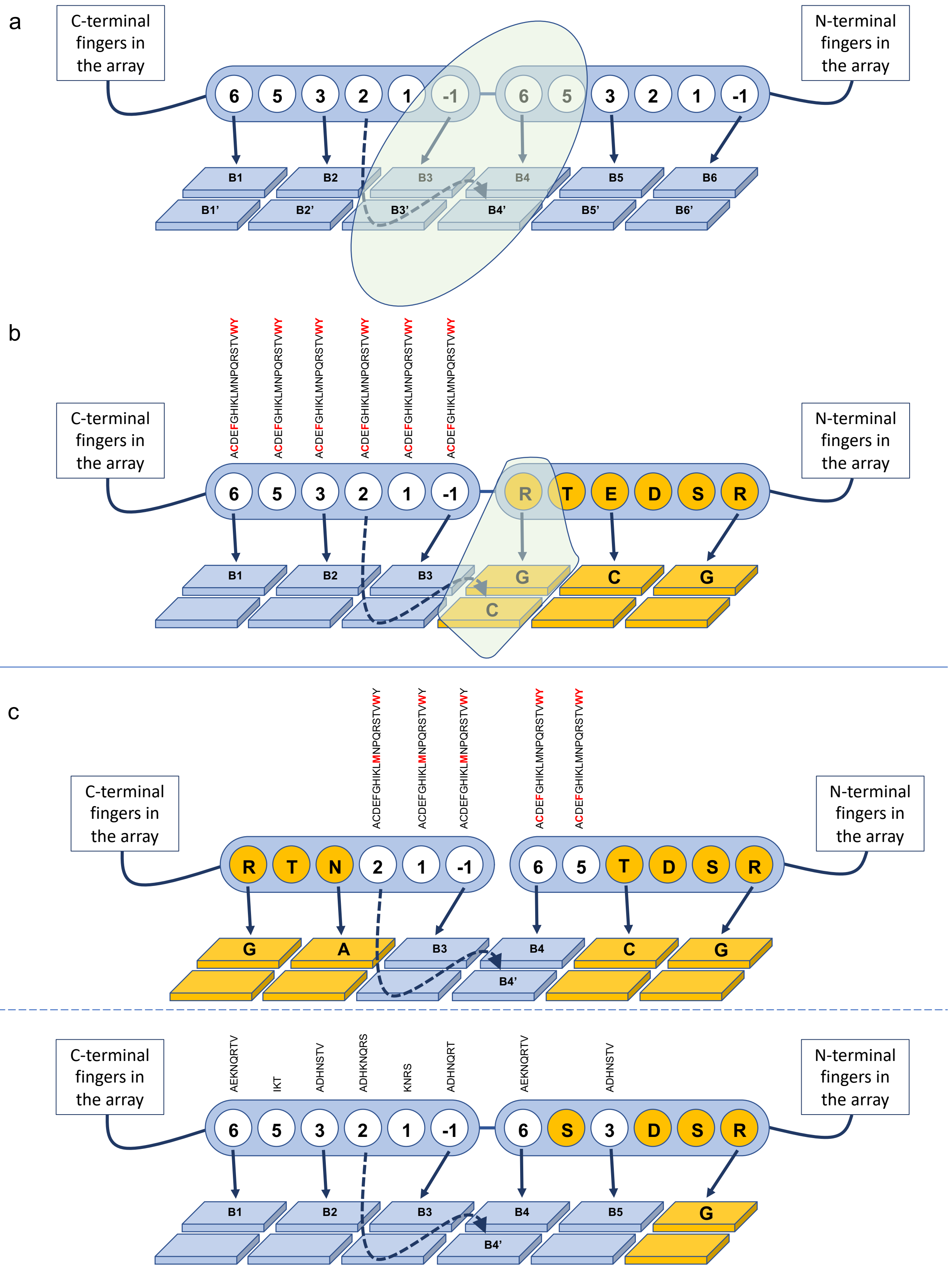
a
C-terminal fingers in the array
N-terminal fingers in the array
6 5 3 2 1 -1 6 5 3 2 1 -1
B1 B2 B3 B4 B5 B6
B1' B2' B3' B4' B5' B6'
b
C-terminal fingers in the array
N-terminal fingers in the array
ACDEFGHIKLMNPQRSTVWY
6 5 3 2 1 -1 R T E D S R
B1 B2 B3 G C G
C
c
C-terminal fingers in the array
N-terminal fingers in the array
ACDEFGHIKLMNPQRSTVWY
R T N 2 1 -1 6 5 T D S R
G A B3 B4 C G
B4'
C-terminal fingers in the array
N-terminal fingers in the array
AEKNQRTV
IKT
ADHNSTV
ADHKNQRS
KNRS
ADHNQRT
AEKNQRTV
ADHNSTV
6 5 3 2 1 -1 6 S 3 D S R
B1 B2 B3 B4 B5 G
B4'

Fig. S2. Zinc finger interface and common selection strategies. A. Cartoon of two adjacent fingers interacting with DNA. The six positions of the helix with base-specifying potential are shown. Position 4 is not shown as it is typically a hydrophobic residue that packs into the core of the domain. Position 4 is not randomized in any selection schemes. The interface and overlap contacts are highlighted with an oval. **B**. Cartoon of a single finger selection approach where all the randomization is on one of the two fingers[4-11]. These were mostly done with an arginine-guanine contact (highlighted) adjacent to the selected finger[4-7,9-11] or, in one case, where the library was the N-terminal finger[6]. On the randomized helix the letter's in bold and red (CFWY) were not coded for in the OPEN and other zinc finger libraries[6,7,10,11]. **C**. Two versions of libraries that selected interface interactions are shown. *Top*. Many of the contacts were fixed with 5 positions incompletely randomized. The red and bold amino acids were not available in these libraries[12,13]. *Bottom*. Another approach randomized more positions but used a very small subset of amino acids. Only available amino acids are listed[14-16].

Library #	Domain 1 helix	Overlap base	Overlap environment	Total helices recovered	Total helical "cores" recovered	successful selections
1	RSDNLRA	A	hydrophobic	383952	27731	97%
1b	RSDNLRA	C	hydrophobic	580513	40882	98%
2	QLATLSN	A	polar	294432	37005	86%
3	DQSNLTR	A	basic	298638	34484	100%
3b	DQSNLTR	G	basic	735906	63649	92%
4	FQSGLIQ	A	polar	398709	27434	97%
5	HKRNLTD	C	acidic	264253	47964	78%
6	DQSALLG	C	small	128306	36919	41%
7	TKQNLTH	C	basic	494026	35203	100%
8	QLATLSY	C	aromatic	293300	31362	97%
9	RNGNLTR	G	basic	1089522	46578	97%
10	YQPNLIN	A	polar	620359	88293	39%

a

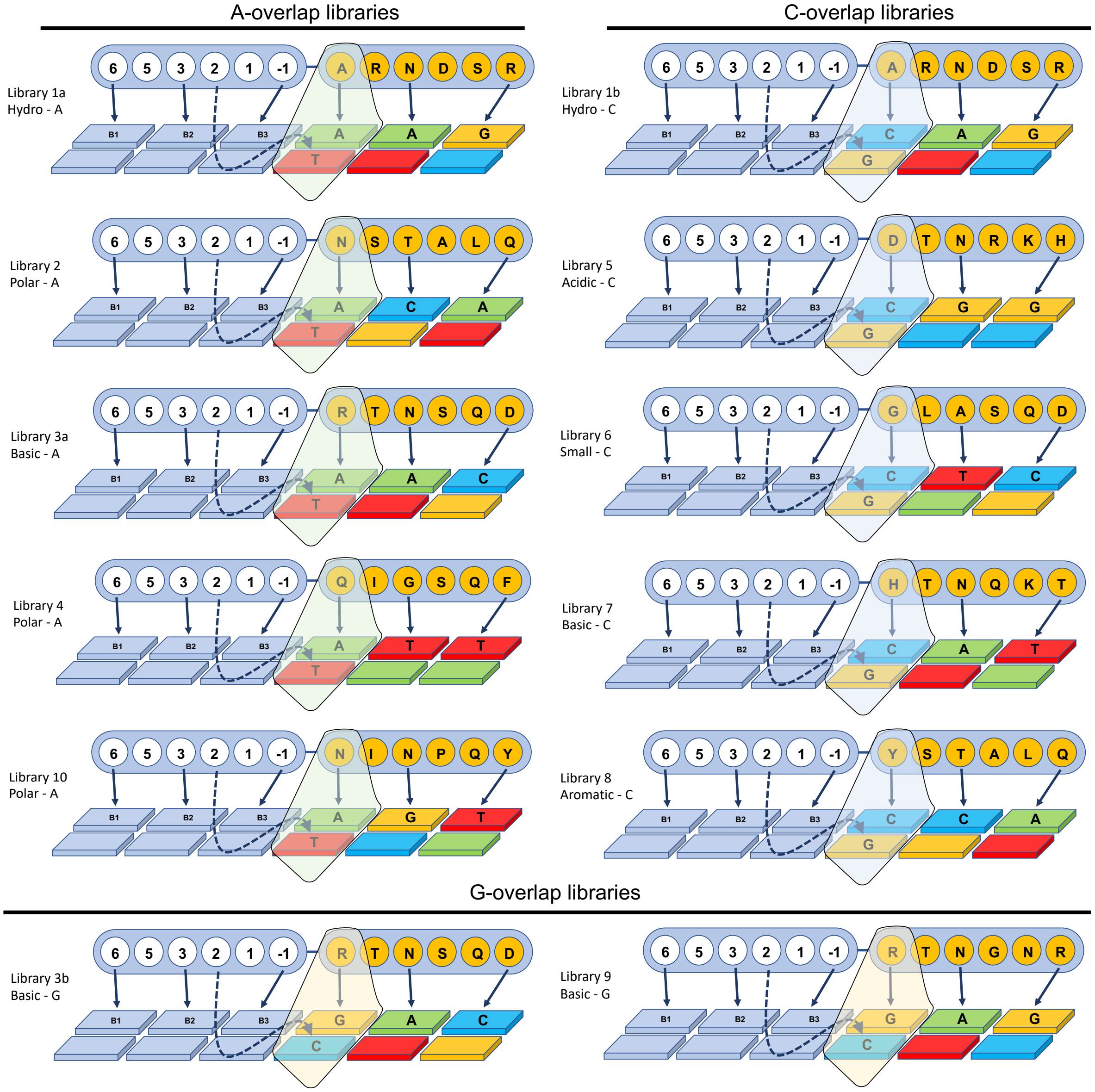

Fig. S3. **List of libraries and interfaces tested. Table**. All libraries screened in this manuscript are listed. The helical residues for the zinc finger adjacent to the library (domain 1 as shown in Figure 1) are shown for each library. The residue presented at the interface (underlined), the overlap base, and the biophysical category of this side chain is noted. Helical enrichment numbers and selection success is also listed. Core helices are defined as the helical residues at positions -1, 2, 3, and 6 that make the primary contacts with the bases of the target site. *Note, library 1a and 1b are the same library using a different base at the overlap position. The same is true for library 3a and 3b. In the manuscript these are referred to as libraries 1(A), 1(C), 3(A), and 3(G) to indicated what overlap base was used in the selections. This is why we have 10 libraries but completed 12 screens.* **a.** Cartoons are shown to depict what environment is presented to the selected zinc finger in each library with A overlaps on the left, C overlaps on the right, and G overlaps at the bottom.

a

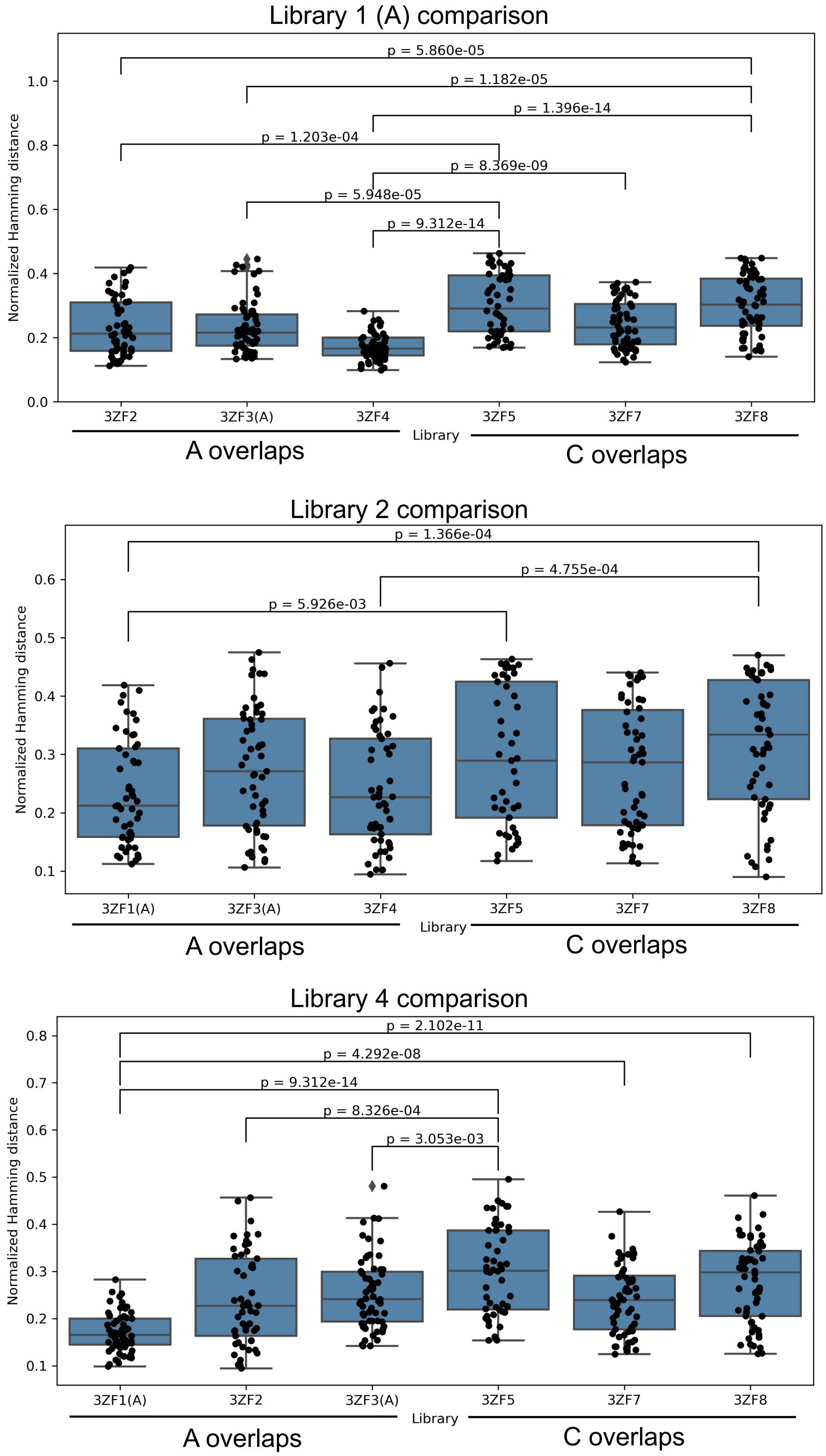

Library 1 (A) comparison
p = 5.860e-05
p = 1.182e-05
p = 1.396e-14
p = 1.203e-04
p = 8.369e-09
p = 5.948e-05
p = 9.312e-14
Normalized Hamming distance
1.0
0.8
0.6
0.4
0.2
0.0
3ZF2
3ZF3(A)
3ZF4
3ZF5
3ZF7
3ZF8
Library
A overlaps
C overlaps
Library 2 comparison
p = 1.366e-04
p = 4.755e-04
p = 5.926e-03
Normalized Hamming distance
0.6
0.5
0.4
0.3
0.2
0.1
3ZF1(A)
3ZF3(A)
3ZF4
3ZF5
3ZF7
3ZF8
Library
A overlaps
C overlaps
Library 4 comparison
p = 2.102e-11
p = 4.292e-08
p = 9.312e-14
p = 8.326e-04
p = 3.053e-03
Normalized Hamming distance
0.8
0.7
0.6
0.5
0.4
0.3
0.2
0.1
3ZF1(A)
3ZF2
3ZF3(A)
3ZF5
3ZF7
3ZF8
Library
A overlaps
C overlaps

b

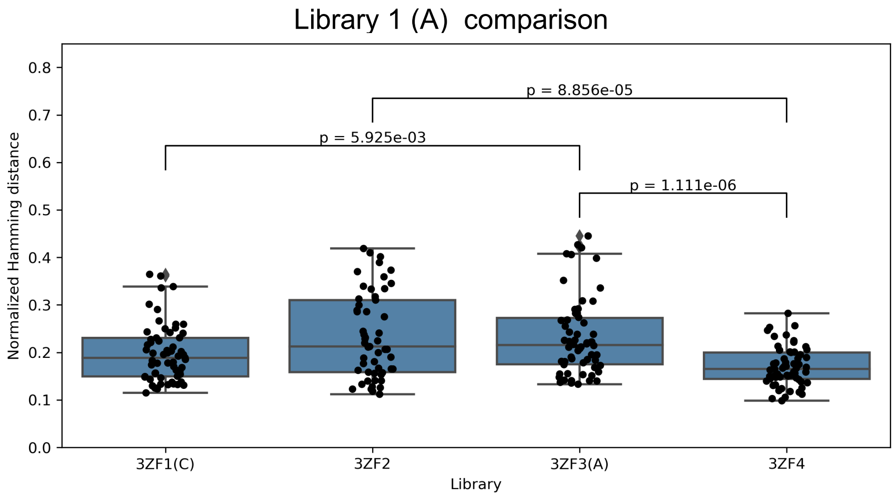
Library 1 (A) comparison
p = 8.856e-05
p = 5.925e-03
p = 1.111e-06
Normalized Hamming distance
0.0
0.1
0.2
0.3
0.4
0.5
0.6
0.7
0.8
3ZF1(C)
3ZF2
3ZF3(A)
3ZF4
Library

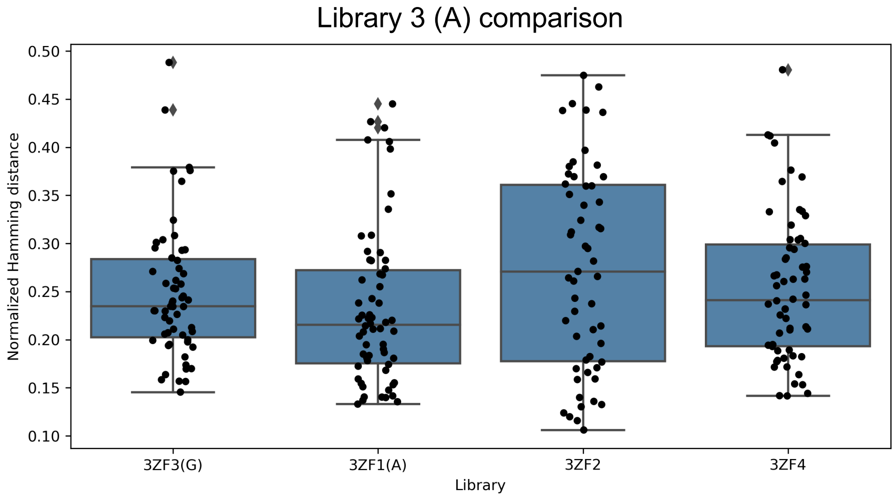
Library 3 (A) comparison
Normalized Hamming distance
0.10
0.15
0.20
0.25
0.30
0.35
0.40
0.45
0.50
3ZF3(G)
3ZF1(A)
3ZF2
3ZF4
Library

c

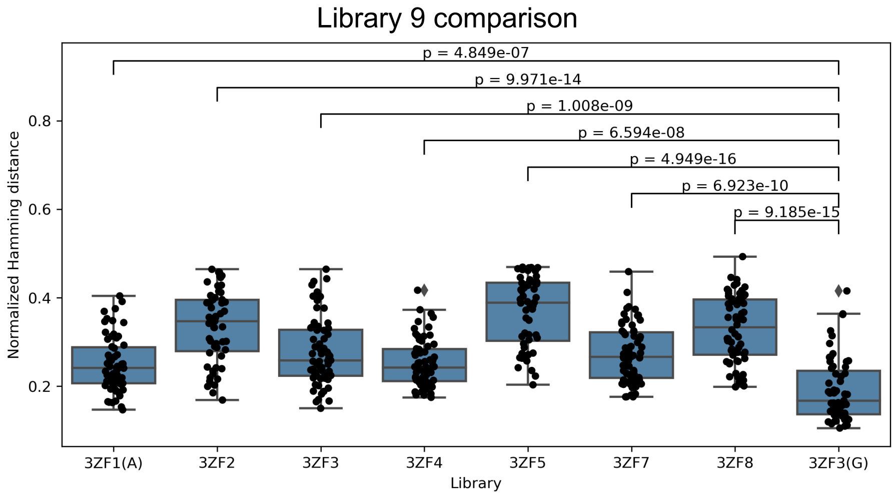
Library 9 comparison
p = 4.849e-07
p = 9.971e-14
p = 1.008e-09
p = 6.594e-08
p = 4.949e-16
p = 6.923e-10
p = 9.185e-15
Normalized Hamming distance
0.2
0.4
0.6
0.8
3ZF1(A)
3ZF2
3ZF3
3ZF4
3ZF5
3ZF7
3ZF8
3ZF3(G)
Library

Fig. S4. Global Hamming distance comparisons for libraries that present different overlap bases at the interface. a. Hamming distance comparison across all successful selections for library 1(A)-top, 2-middle, and 4-bottom, with the remaining libraries that were successful across most target selections (two-sided Wilcoxon rank-sum test). Libraries 6 and 10 were omitted because of their poor performance. A-overlap libraries are to the left and C-overlap libraries to the right. Libraries 1(A), 2, and 4 all bind adenine at the overlap and for the most part they are more similar to other A-overlap libraries than they are to C-overlap libraries. **b**. Libraries 1 and 3 are able to bind A or C and A or G, respectively, at the overlap. A comparison of these libraries using A at the overlap demonstrated that the same library with a different base at the overlap is approximately as similar as the comparison to other A overlap selections (two-sided Wilcoxon rank-sum test). **c**. A comparison of **library 9**, *that uses an arginine-guanine contact at the interface*, is significantly more similar to the only other library screened that also placed an arginine-guanine contact at the overlap **library 3(G)**, than compared to any other library screened (two-sided Wilcoxon rank-sum test).

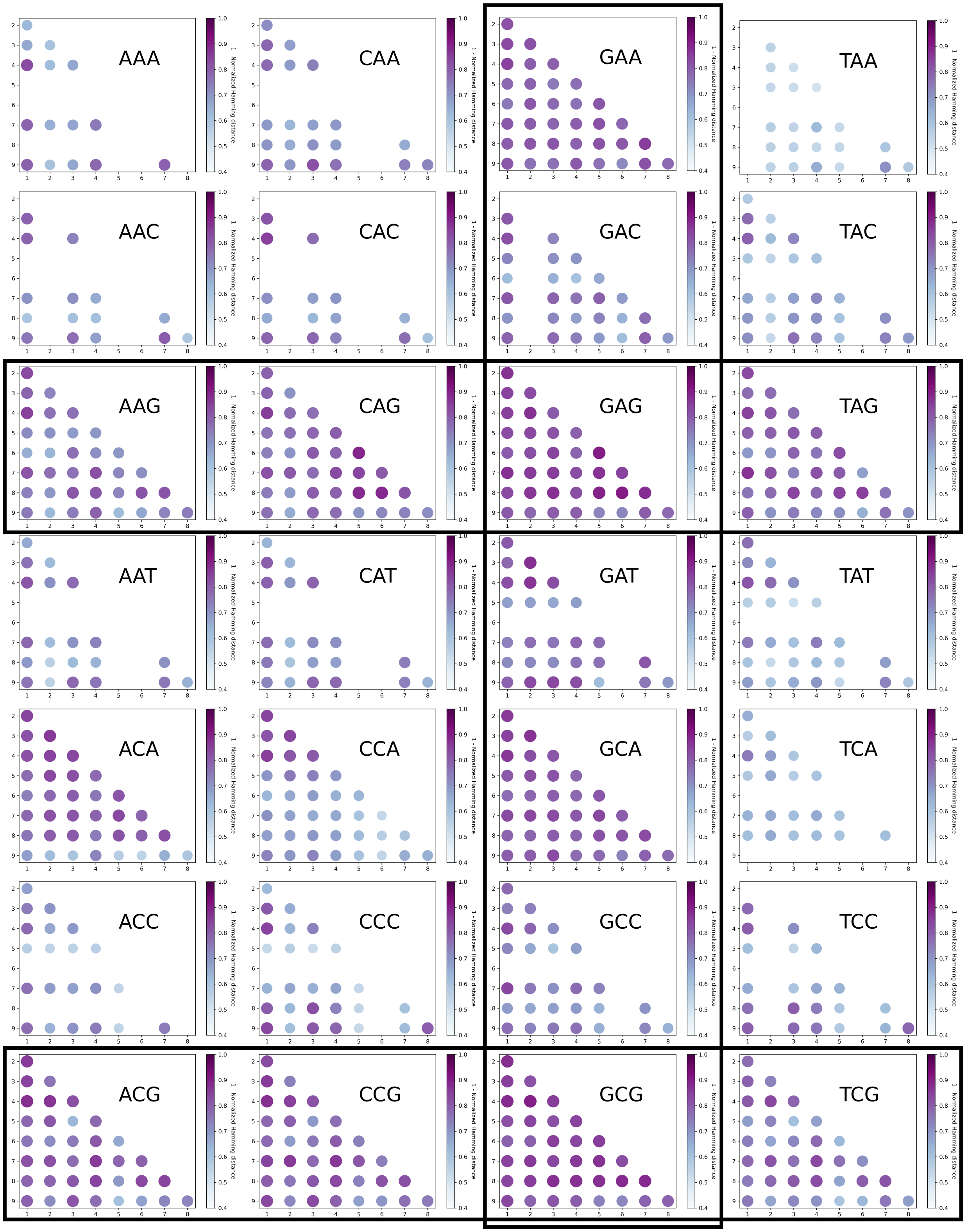
AAA
CAA
GAA
TAA
AAC
CAC
GAC
TAC
AAG
CAG
GAG
TAG
AAT
CAT
GAT
TAT
ACA
CCA
GCA
TCA
ACC
CCC
GCC
TCC
ACG
CCG
GCG
TCG
1 - Normalized Hamming distance

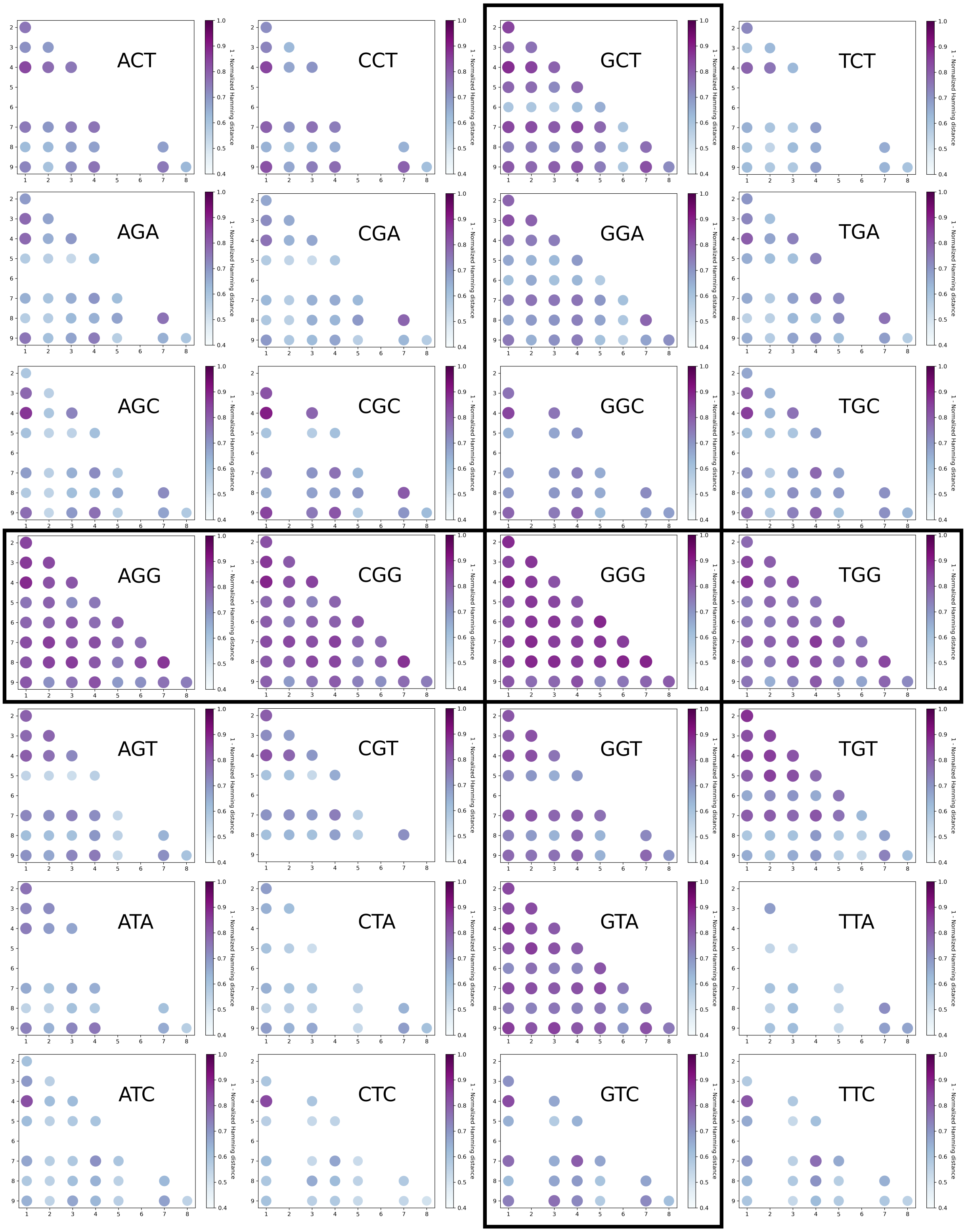
ACT
CCT
GCT
TCT
AGA
CGA
GGA
TGA
AGC
CGC
GGC
TGC
AGG
CGG
GGG
TGG
AGT
CGT
GGT
TGT
ATA
CTA
GTA
TTA
ATC
CTC
GTC
TTC
1 - Normalized Hamming distance

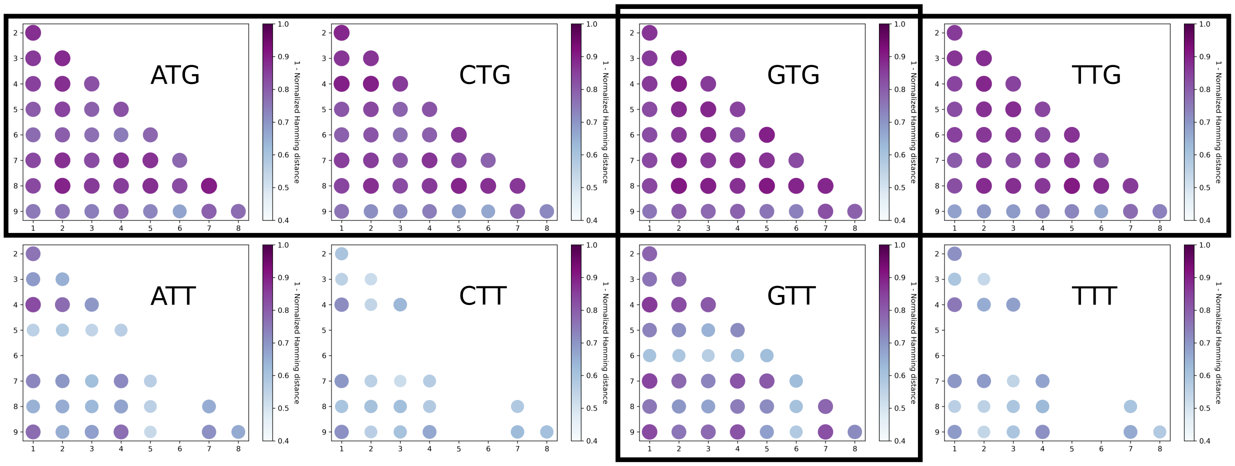

Fig. S5. **1-Hamming distance dot plot comparison of libraries by target sequence**. Here we compare the similarity of all successful selections for the screens of the primary libraries 1 thru 9 for all 64 triplets. As the plot is 1 – Hamming distance, the darker the dot, the more similar the selections. An empty space indicates that the selection for one or both of the libraries failed and therefore no comparison can be made. All plots are on a scale of 0.4 to 1 so that comparisons can be made between plots. GNN (vertical) and NNG (horizontal) targets are boxed to highlight how similar these selections.

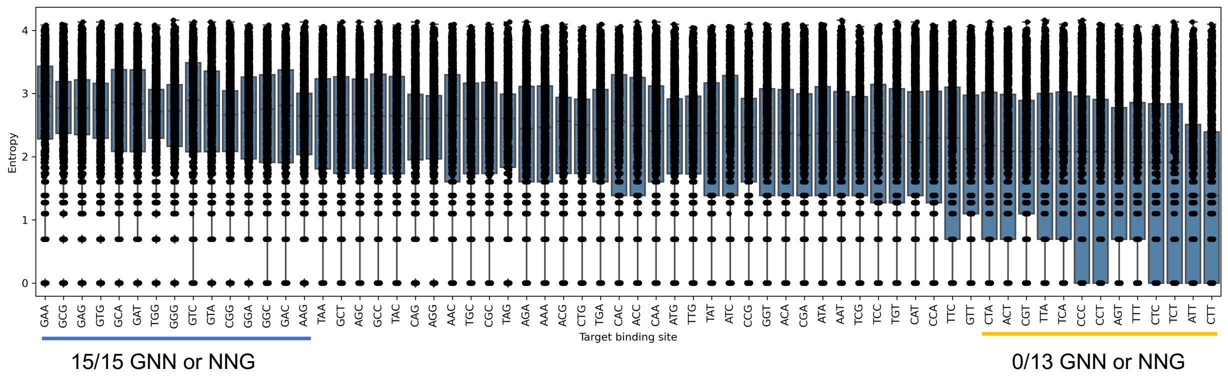

Fig. S6. Promiscuity of G-rich binding. For the helices enriched in the target selections shown we calculated the number of alternative binding sites these helices were also recovered in and computed the entropy of the resulting position weight matrix. Therefore, the target entropy provides a measure of the general specificity or promiscuity of the helices recovered in these selections. The distribution of entropies is shown as a boxplot. Note the top 15 binding sites produce helices with the most target entropy and these are exclusive composed of GNN and NNG targets. Conversely, there are no GNN or NNG target in the 13 selections with the lowest target entropy and only 2 of the bottom 24. The full distribution for each target selection is shown over the corresponding boxplot.

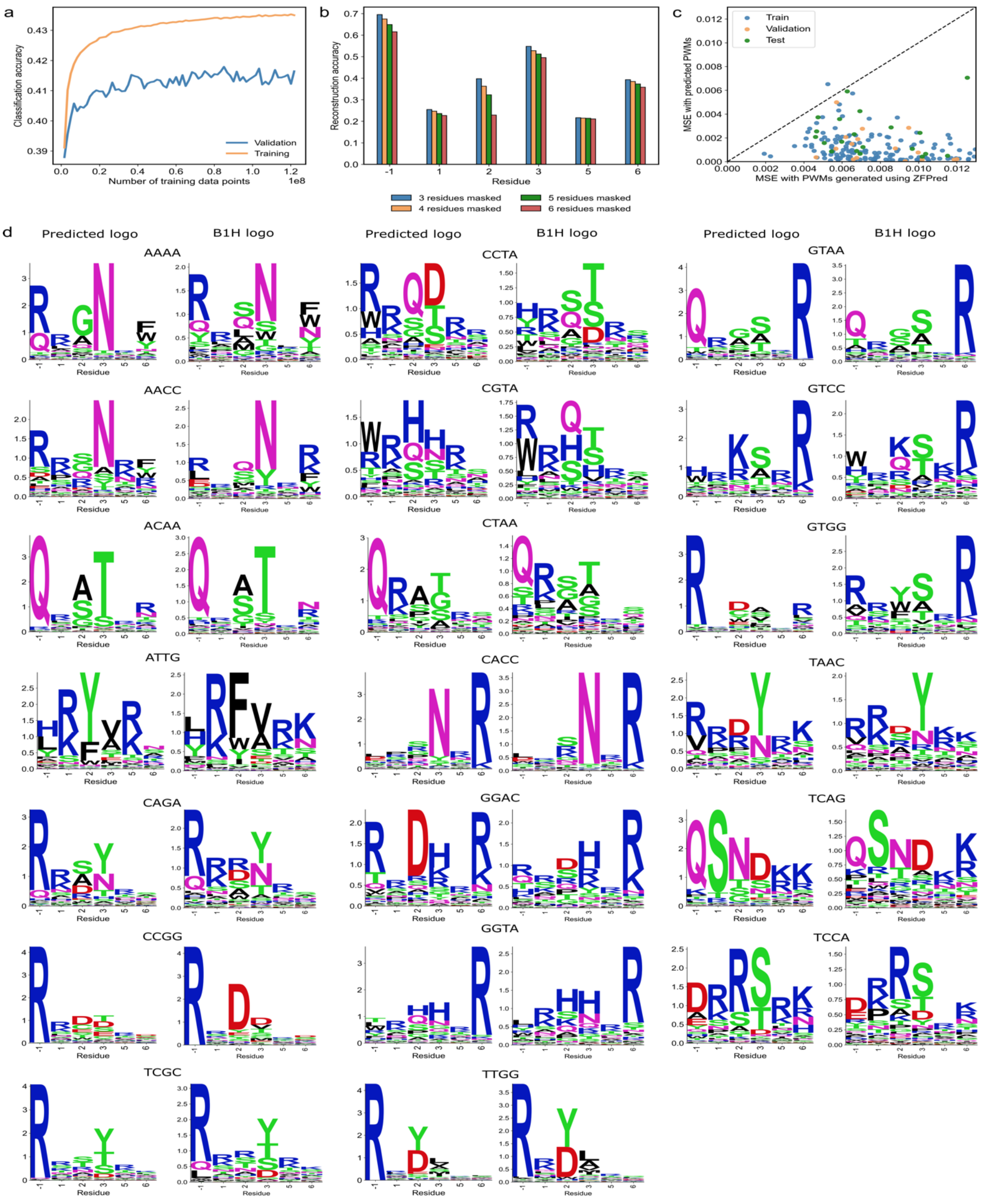

Fig. S7. Performance of single-helix design modules. a) Training and validation accuracy during pre-training step. **b)** Helix sequence reconstruction accuracy with different numbers of masked residues. **c)** Comparison of differences between predicted and real selection logos using the developed model and ZFPred. **d)** Predicted logos and real B1H logos for test set sequences.

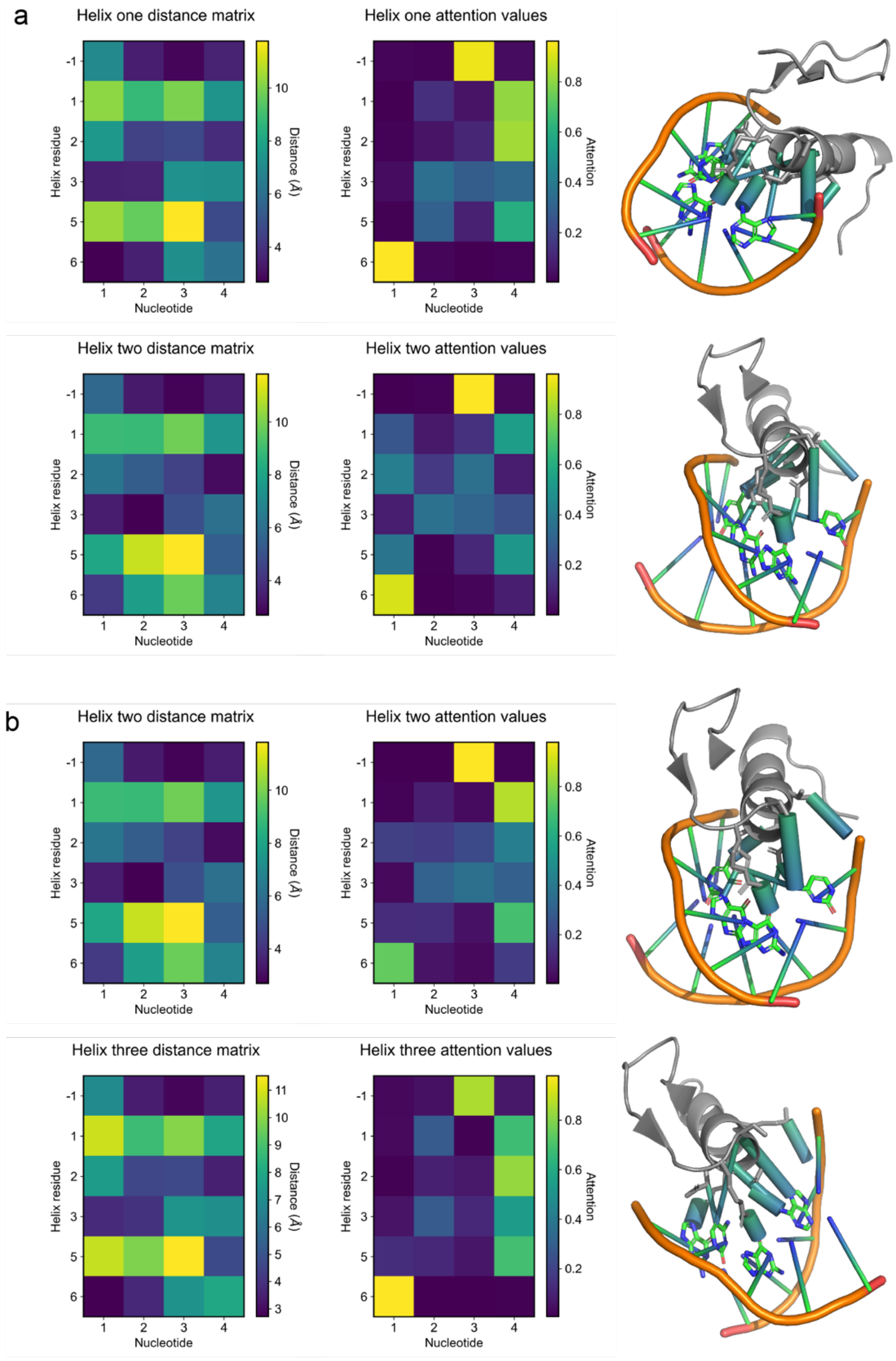

Fig. S8. Attention values in a layer one and head four of modules one and two compared to distances between nucleotides and residues in Zif268. Attention values and distances for the first **a)** and second **b)** helix pairs in Zif268. Attention values are represented by the width of the cyan cylinders in the structural figures, with attention values >= 0.2 shown.

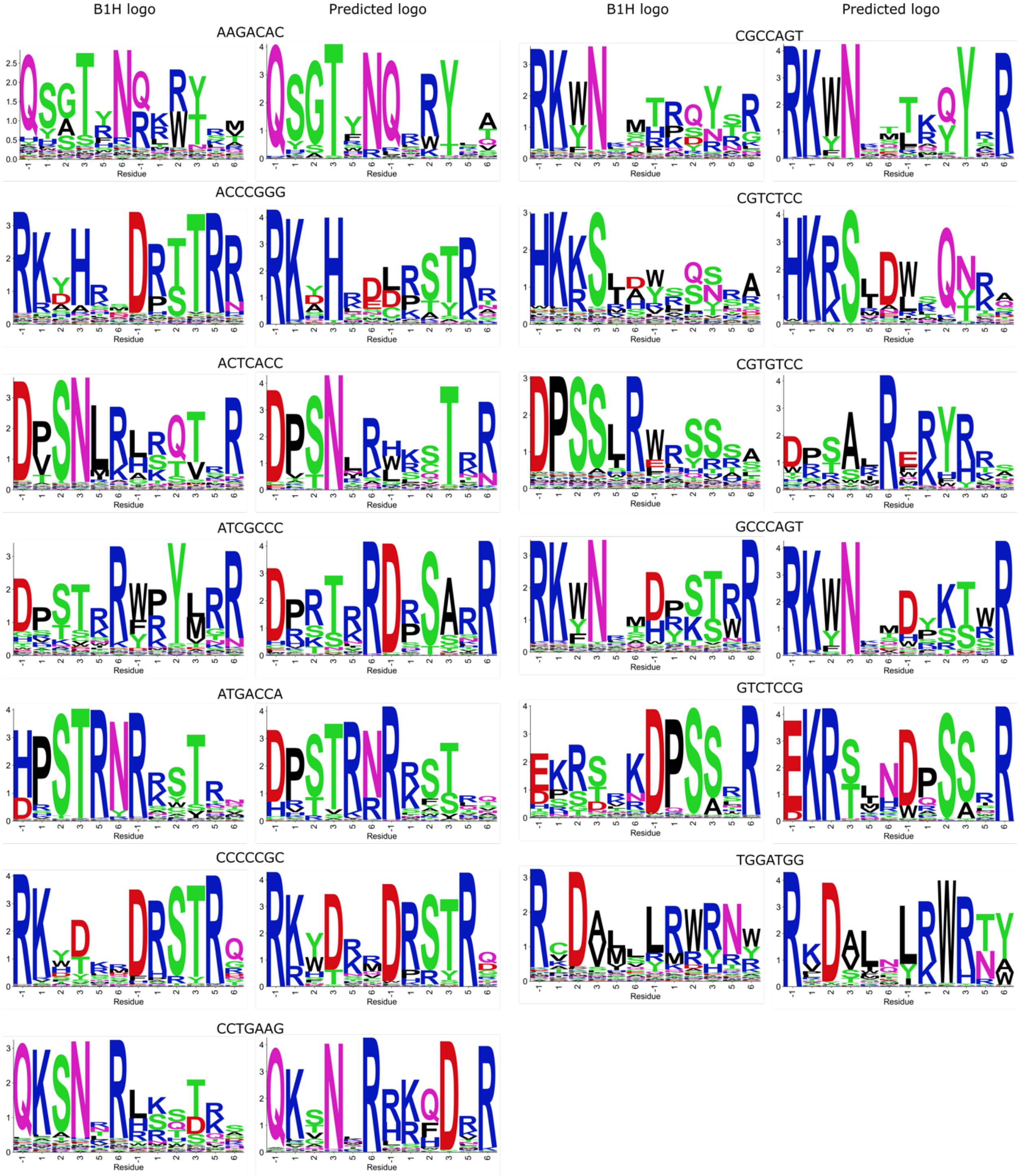

Fig. S9. Predicted logos, real B1H logos, and concatenated single-helix B1H logos for all test set sequences.

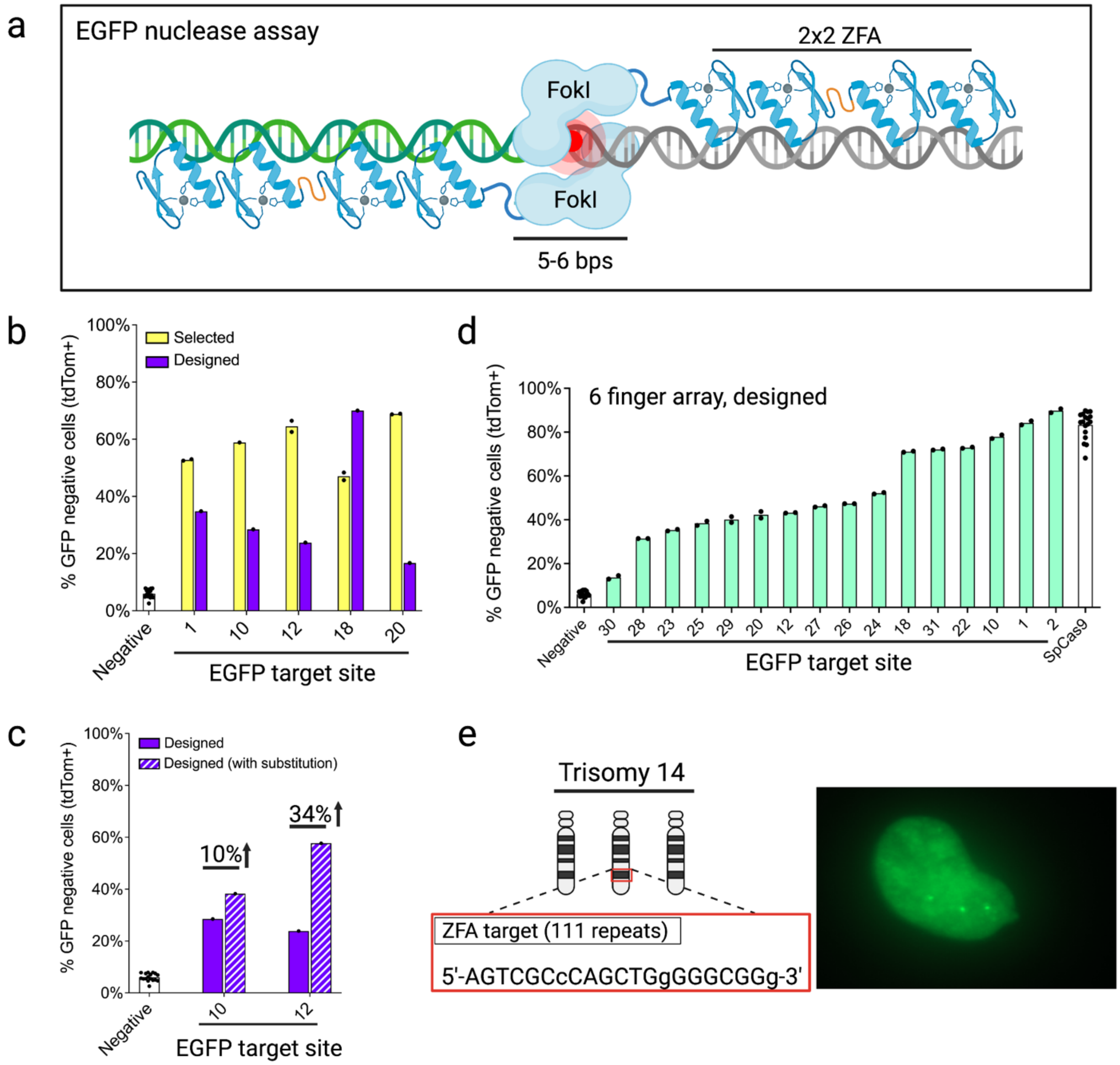

Fig. S10. Designed Zinc Finger Nucleases (**A**) ZFNs bind DNA as dimers in a tail-to-tail orientation, spaced by 5 or 6bp. The cartoon shows each monomer with two pairs of ZFs separated by a base-skipping linker, for a total 8-finger ZFN. (**B**) A comparison of loss of fluorescence in a GFP disruption assay for 8-finger ZFNs that were either selected or designed to cut the same targets. (**C**) Substitution of 2 of the 8-fingers in designed arrays with selected fingers increase activity. (**D**) Sixteen 12-finger ZFNs, 6 per monomer, are tested for loss of fluorescence. (**E**) A six-finger array was designed to bind a repeat sequence on chromosome 14, expressed as a GFP fusion, and visualized by live cell imaging.

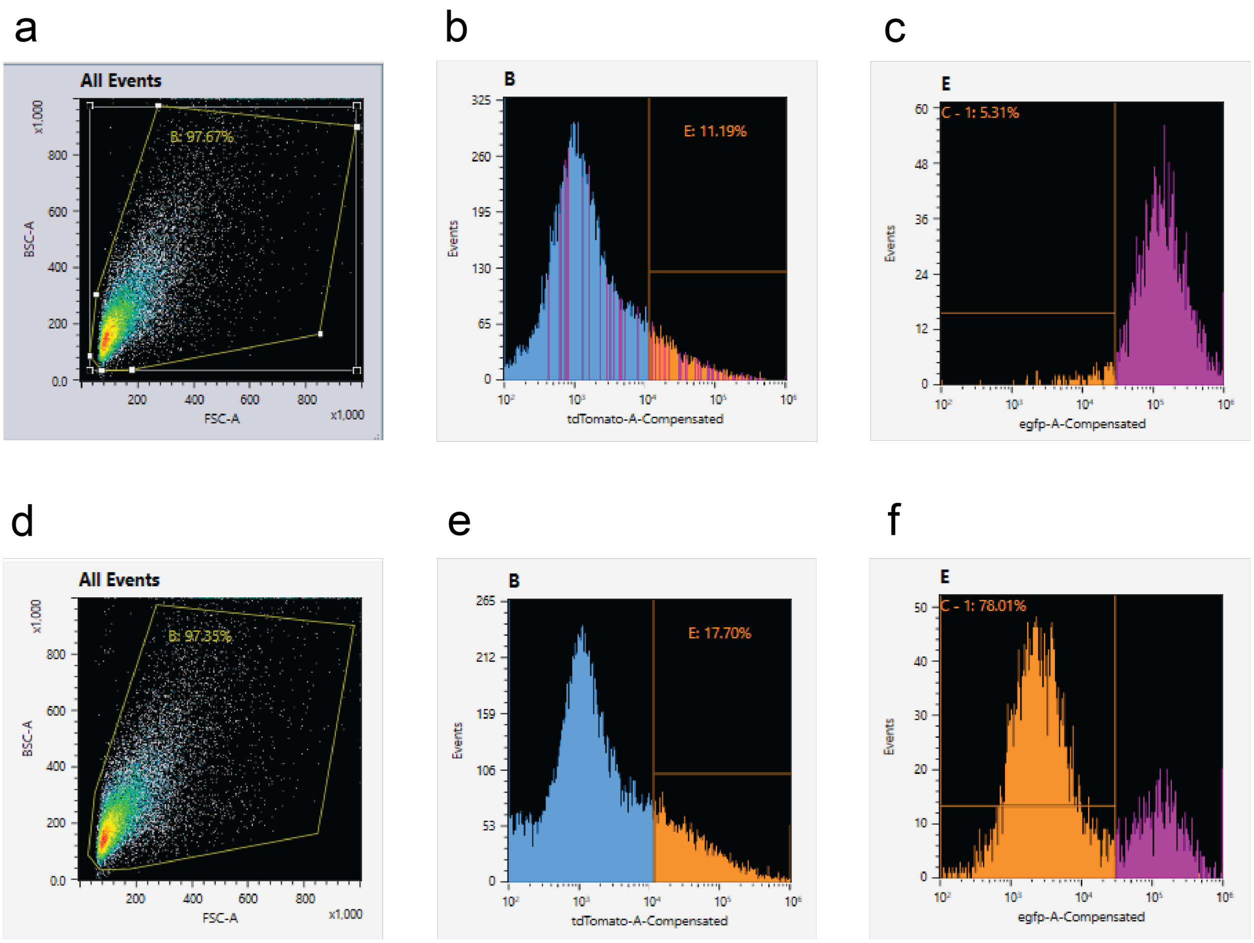

Fig. S11. Cytometry plots of positive and negative controls for ZFN assay.
a) Negative control – depicts all events (20,000) and gating strategy for subsequent plot.
b) Negative control – depicts gating strategy to define tdTomato$^+$ events.
c) Negative control – depicts gating strategy to define EGFP$^-$ (negative) events that are also tdTomato$^+$.
d-f) Positive control– as above.

DNA targets (5' to 3')

Forward	1x TetO (For) = GTCTCTATCACTGATAGGGAGA<br>Tet1 For = GTCTCTaTCACTGaTAGGGAg<br>Tet2 For = TCTCTAtCACTGAtAGGGAGa
Reverse	1x TetO (Rev) = TCTCCCTATCAGTGATAGAGAC<br>Tet3 Rev = TCTCCCtATCAGTgATAGAGa<br>Tet4 Rev = CTCCCTaTCAGTGaTAGAGAc

6 ZF helices (Nterm to Cterm)

Tet1 For = QKVHLQS RKWTLSV RKGTLQD QYSSLYK RKGDLNK DPSSLRR

Tet2 For = RKYNLLR RRYSLSA QKAHLLS DPSNLRR QKRLLQN WKVDLRK

Tet3 Rev = RKFNLLR QSNTLRT LKHHLLN TSSGLCH EKRTLLN WKVDLRK

Tet4 Rev = QKTHLLT RRDYLTK RKFTLLR QSNDLRK LKQTLQD RRDRLRR

>ZIM3 scaffold_Tet3_Rev
MNNSQGRVTFEDVTVNFTQGEWQRLNPEQRNLYRDVMLENYSNLVSVGQGETTKP
DVILRLEQGKEPWLEEEEVLGSGRAEKNGDIGGQIWKPKDVKESLAREVPSINKETLT
TQKGVECDGSKKILPLGIDDVSSLQHYVQNNSHDDNGYRKLVGNNPSKFVGQQ
FACDICGRKFARKFNLLRHTRIHTGEKPFACDICGRKFAQSNTLRTHTKIHTQRPQIPPKP
FACDICGRKFALKHHLLNHTRIHTGEKPFACDICGRKFATSSGLCHHTKIHTQRPQIPPKP
FACDICGRKFAEKRTLLNHTRIHTGEKPFACDICGRKFAWKVDLRKHTKIHSR*
(ZIM3 repressor scaffold, ZF scaffold, Helix, Base-skipping linker)

>KLF6 scaffold_Tet3_Rev
MDVLPMCSIFQELQIVHETGYFSALPSLEEYWQQTCLELERYLQSEPCYVSAS
EIKFDSQEDLWTKIILAREKKEESELKISSSPPEDTLISPSFCYNLETNSLNSDV
SSESSDSSEELSPTAKFTSDPIGEVLVSSGKLSSSVTSTPPSSPELSREPS
QLWGCVPGELPSPGKVRSGTSGKPGDKGNGDASPDGRRRV
FACDICGRKFARKFNLLRHTRIHTGEKPFACDICGRKFAQSNTLRTHTKIHTQRPQIPPKP
FACDICGRKFALKHHLLNHTRIHTGEKPFACDICGRKFATSSGLCHHTKIHTQRPQIPPKP
FACDICGRKFAEKRTLLNHTRIHTGEKPFACDICGRKFAWKVDLRKHTKIHSR*
(KLF6 activator scaffold, ZF scaffold, Helix, Base-skipping linker)

Fig. S12. Zinc finger arrays that target the TetO sequence for both activation (KLF6) or repression (Zim3). Top box, the TetO sequence is listed in the forward (for) and reverse (rev) direction. Two registers of these sequences were used as the target for zinc finger arrays shown below each and numbered Tet1 – Tet4. Lowercase letters indicate the base that is skipped between 2-finger modules. **Bottom box**, the helices used to specify each of the Tet target sequences are listed as they are expressed in the protein from N-term to C-term. **Below**, the template sequences for where these helices are expressed in the RTFs KLF6 and Zim3 are shown.

a.

>KLF7 scaffold_Tet3_Rev
MDVLASYSIFQELQLVHDTGYFSALPSLEETWQQTCLELERYLQTEPRRISETFGEDLDCFLHAS
PPPCIEESFRRLDPLLLPVEAAICEKSSAVDILLSRDKLLSETCLSLQPASSSLDSYTAVNQAQLN
AVTSLTPPSSPELSRHLVKTSQTLSAVDGTVTLKLVAKKAALSSVKVGGVATAAAAVTAAGAVK
SGQSDSDQGGLGAEACPENKKRVPFACDICGRKFARKFNLLRHTRIHTGEKPFACDICGRKFA
QSNTLRTHTKIHTQRPQIPPKPFACDICGRKFALKHHLLNHTRIHTGEKPFACDICGRKFA
TSSGLCHHTKIHTQRPQIPPKPFACDICGRKFAEKRTLLNHTRIHTGEKPFACDICGRKFA
WKVDLRKHTKIHI*
(KLF7 activator scaffold, ZF scaffold, Helix, Base-skipping linker)

>FOXR2 scaffold_Tet3_Rev
MDLKLKDCEFWYSLHGQVPGLLDWDMRNELFLPCTTDQCSLAEQILAKYRVGVMKPP
EMPQKRRPSPDGDGPPCEPNLWMWVDPNILCPLGSQEAPKPSGKEDLTNISPFPQPPQK
DEGSNCSEDKVVESLPSSSSEQSPLQKQGIHSPSDFELTEEEAEEPDDNSLQSPEMKCYQS
QKLWQINNQEKSFACDICGRKFARKFNLLRHTRIHTGEKPFACDICGRKFA
QSNTLRTHTKIHTQRPQIPPKPFACDICGRKFALKHHLLNHTRIHTGEKPFACDICGRKFA
TSSGLCHHTKIHTQRPQIPPKPFACDICGRKFAEKRTLLNHTRIHTGEKPFACDICGRKFA
WKVDLRKHTKIHIQECMSQPELLTSLFDL*
(FOXR2 activator scaffold, ZF scaffold, Helix, Base-skipping linker)

>ZXDC scaffold_Tet3_Rev
MDLPALLPAPTARGGQHGGGPGPLRRAPAPLGASPARRRLLLVRGPEDGGPGAR
PGEASGPSPPPAEDDSDGDSFLVLLEVPHGGAAAEAAGSQEAEPGSRVNLASRP
EQGPSGPAAPPGPGVAPAGAVTISSQDLLVRLDRGVLALSAPPGPATAGAAAP
RRAPQASGPSTPGFACDICGRKFARKFNLLRHTRIHTGEKPFACDICGRKFA
QSNTLRTHTKIHTQRPQIPPKPFACDICGRKFALKHHLLNHTRIHTGEKPFACDICGRKFA
TSSGLCHHTKIHTQRPQIPPKPFACDICGRKFAEKRTLLNHTRIHTGEKPFACDICGRKFA
WKVDLRKHTKIHSRRQDLLPQLEAPSSLTPSSELSSPGQSELTNMDLAALFSDTPANAS
GSAGGSDEALNSGILTIDVTSVSSSLGGNLPANNSSLGPMEPLVLVAHSDIPPSLDSPLVL
GTAATVLQQGSFSVDDVQTVSAGALGCLVALPMKNLSDDPLALTSNSNLAAHITTPTSSS
TPRENASVPELLAPIKVEPDSPSRPGAVGQQEGSHGLPQSTLPSPAEQHGAQDTELSAGT
GNFYLESGGSARTDYRAIQLAKEKKQRGAGSNAGASQSTQRKIKEGKMSPPHFHASQNSW
LCGSLVVPSGGRPGPAPAAGVQCGAQGVQVQLVQDDPSGEGVLPSARGPATFLPFLTVDL
PVYVLQEVLPSSGGPAGPEATQFPGSTINLQDLQ*
(ZXDC activator scaffold, ZF scaffold, Helix, Base-skipping linker)

b.

>ZNF10 scaffold_Tet3_Rev
MDAKSLTAWSRTLVTFKDVFVDFTREEWKLLDTAQQIVYRNVMLENYKNLVSLGYQLTKP
DVILRLEKGEEPWLVEREIHQETHPDSETAFEIKSSVSSRSIFKDKQSCDIKMEGMARNDL
WYLSLEEVWKCRDQLDKYQENPERHLRQVAFTQKKVLTQERVSESGKYGGNCLLPAQLVL
REYFHKRDSHTKSLKHDLVLNGHQDSCASNSNECGQTFCQNIHLIQFARTHTGDKSYKCP
DNDNSLTHGSSLGISKGIHREKPFACDICGRKFARKFNLLRHTRIHTGEKPFACDICGRKFA
QSNTLRTHTKIHTQRPQIPPKPFACDICGRKFALKHHLLNHTRIHTGEKPFACDICGRKFA
TSSGLCHHTKIHTQRPQIPPKPFACDICGRKFAEKRTLLNHTRIHTGEKPFACDICGRKFA
WKVDLRKHTKIHTGEQFLTCNQCGTALVNTSNLIGYQTNHIRENAY*
(ZNF10 repressor scaffold, ZF scaffold, Helix, Base-skipping linker)

>ZNF264 scaffold_Tet3_Rev
MAAAVLTDRAQVSVTFDDVAVTFTKEEWGQLDLAQRTLYQEVMLENCGLLVSLGCPVPKA
ELICHLEHGQEPWTRKEDLSQDTCPGDKGKPKTTEPTTCEPALSEGISLQGQVTQGNSVD
SQLGQAEDQDGLSEMQEGHFRPGIDPQEKSPGKMSPECDGLGTADGVCSRIGQEQVSPG
DRVRSHNSCESGKDPMIQEEENNFACDICGRKFARKFNLLRHTRIHTGEKPFACDICGRKFA
QSNTLRTHTKIHTQRPQIPPKPFACDICGRKFALKHHLLNHTRIHTGEKPFACDICGRKFA
TSSGLCHHTKIHTQRPQIPPKPFACDICGRKFAEKRTLLNHTRIHTGEKPFACDICGRKFA
WKVDLRKHTKIHTGKNPISVTDVGRPFTSGQTSVTLRELLLLGKDFLNVTTEANILPEET
SSSASDQPYQRETPQVSSL*
(ZNF264 repressor scaffold, ZF scaffold, Helix, Base-skipping linker)

>ZNF324 scaffold_Tet3_Rev
MAFEDVAVYFSQEEWGLLDTAQRALYRRVMLDNFALVASLGLSTSRPRVVIQLERGE
EPWVPSGTDTTLSRTTYRRRNPGSWSLTEDRDVSGEWPRAFPDTPPGMTTSVFPAVG
ACHSVKSLQRQRGASPSRERKPTGVSVIYWERLLLGSGSGQASVSLRLTSPLRPPEGVRL
REKTLTEHALLGRQPRTPERQKPCAQEVPGRTFGSAQDLEAAGGRGHHRMGAVWQEPHR
LLGGQEPSTWDELGEALHAGEKSFACDICGRKFARKFNLLRHTRIHTGEKPFACDICGRKFA
QSNTLRTHTKIHTQRPQIPPKPFACDICGRKFALKHHLLNHTRIHTGEKPFACDICGRKFA
TSSGLCHHTKIHTQRPQIPPKPFACDICGRKFAEKRTLLNHTRIHTGEKPFACDICGRKFA
WKVDLRKHTKIHTGEKTVRRSRASLHPQARSVAGASSEGAPAKETEPTPASGPAAVSQPAEV*
(ZNF324 repressor scaffold, ZF scaffold, Helix, Base-skipping linker)

Fig. S13. Reprogrammed transcription factor sequences with the Tet3 zinc fingers. a) The sequence for the activating RTFs, color coded with the parent protein purple, the zinc finger array blue, helices black, and base-skipping linker orange. **b)** The sequence for the KRAB containing RTFs for repression, color coded with the parent protein red, the zinc finger array blue, helices black, and base-skipping linker orange.

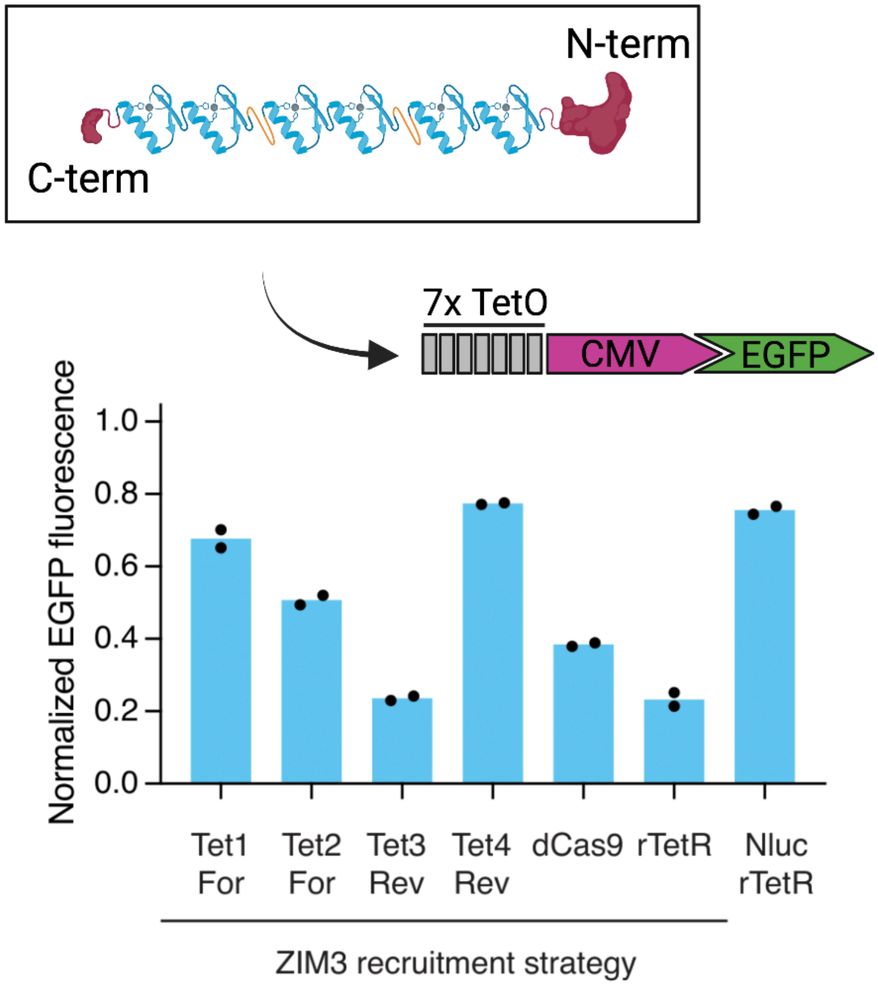

Fig. S14. EGFP repression by ZIM3 RTFs. The zinc fingers of ZIM3 were replaced with the TetO-binding zinc finger arrays described in Figures 5 and S11. These were expressed in a HEK293T cell line with EGFP expression driven by a constitutive promoter. EGFP fluorescence relative to controls are shown.

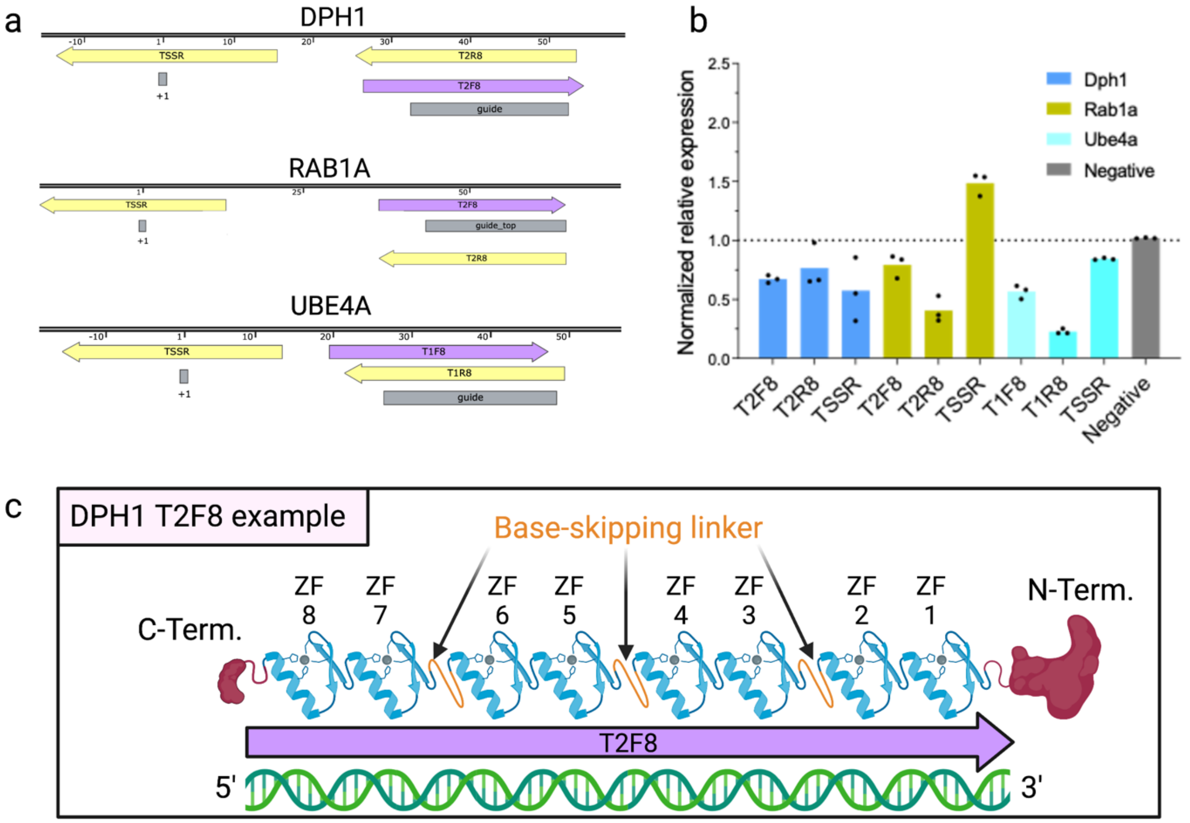

DPH1 T2F8 ZF helices (-1, 1, 2, 3, L, 5, 6)

	ZF1	ZF2	ZF3	ZF4	ZF5	ZF6	ZF7	ZF8	
N.term-	RKWNLLM	RSTNLRD	YPYLLRN	ERSKLRR	RVDTLLD	HLSNLRK	DPSALIR	RLDVLRA	-C.term

DPH1 T2F8 DNA target

5'-CTGGTCgTATCCGgGGCAGCgGAGCAGg-3'

>DPH1_T2F8
MNNSQGRVTFEDVTVNFTQGEWQRLNPEQRNLYRDVMLENYSNLVSVGQGETTKPDVILRL
EQGKEPWLEEEEVLGSGRAEKNGDIGGQIWKPKDVKESLAREVPSINKETLTTQKGVECDGSK
KILPLGIDDVSSLQHYVQNNSHDDNGYRKLVGNNPSKFVGQQFACDICGRKFARKWNLLMHT
RIHTGEKPFACDICGRKFARSTNLRDHTKIHTQRPQIPPKPFACDICGRKFAYPYLLRNHTRIHT
GEKPFACDICGRKFAERSKLRRHTKIHTQRPQIPPKPFACDICGRKFARVDTLLDHTRIHTGEKP
FACDICGRKFAHLSNLRKHTKIHTQRPQIPPKPFACDICGRKFADPSALIRHTRIHTGEKPFACDI
CGRKFARLDVLRAHTKIHSR*

(ZIM3 scaffold, ZF scaffold, Helix, Base-skipping linker)

Fig. S15. Repression of endogenous genes with ZIM3 RTFs. a) Three zinc finger arrays were designed to bind sequences near the TSS of DPH1, RAB1a, and UBE4A as shown. The position of the active gRNA used by spCas9 is also shown for comparison. **b**) expression levels as measured by RT-qPCR are shown for each RTF. **c**) Cartoon and sequence of the DPH1 T2F8 RTF is shown for reference and clarity. In all RTFs, the ZIM3 (red) and ZF scaffold (blue) are the same with only the black helical residues changing per construct (see Supplemental Data for all sequences).

a

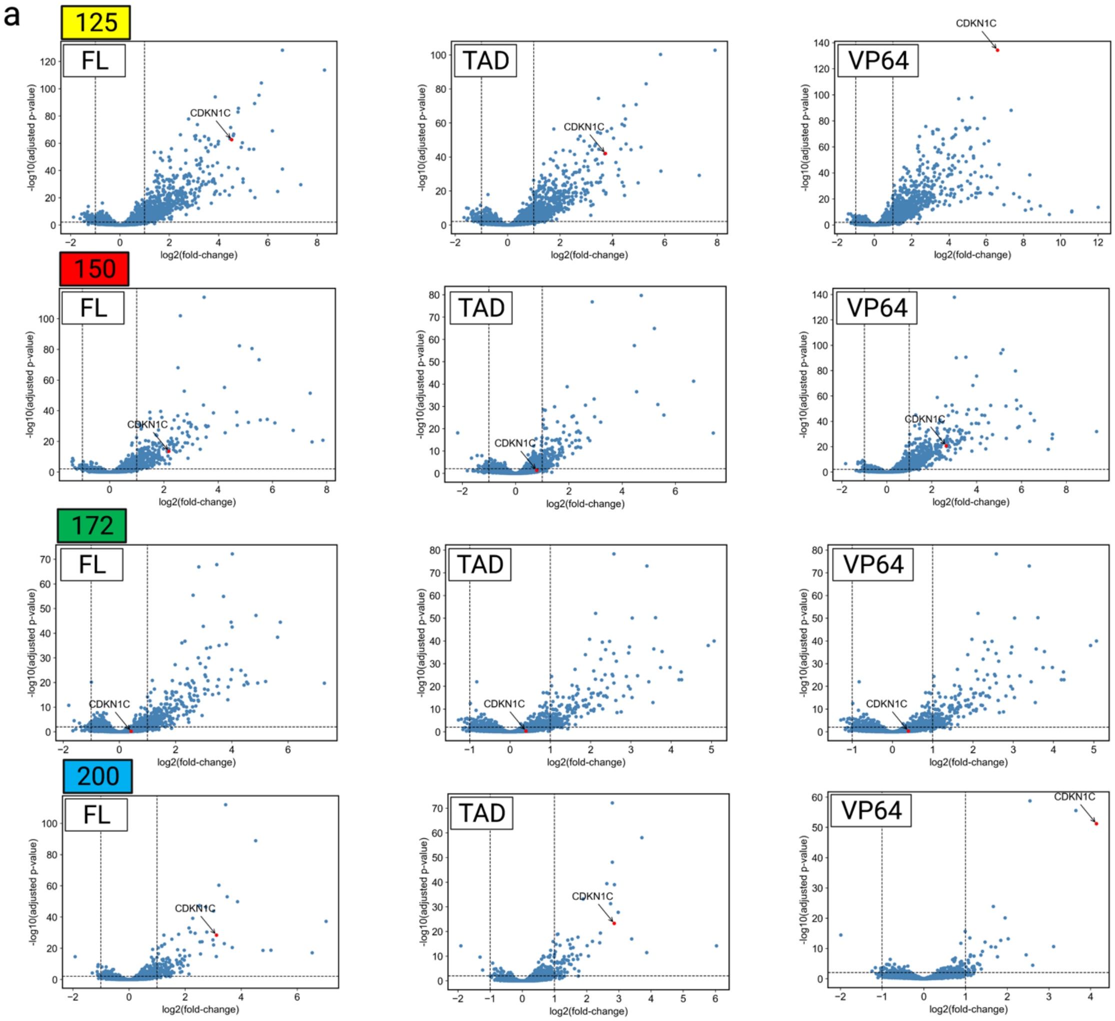

b

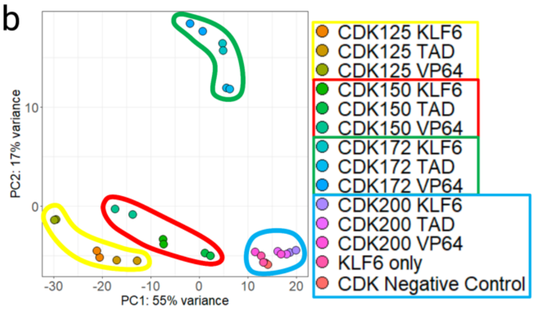

c

	CDK125 FL	CDK125 TAD	CDK125 VP64	CDK150 FL	CDK150 TAD	CDK150 VP64	CDK172 FL	CDK172 TAD	CDK172 VP64	CDK200 FL	CDK200 TAD	CDK200 VP64
CDK125 FL	1173											
CDK125 TAD	865	972										
CDK125 VP64	841	732	1674									
CDK150 FL	324	300	404	567								
CDK150 TAD	169	163	192	270	295							
CDK150 VP64	428	384	744	464	240	945						
CDK172 FL	216	209	255	163	98	197	683					
CDK172 TAD	209	204	276	152	100	211	427	598				
CDK172 VP64	302	272	504	215	113	371	468	461	947			
CDK200 FL	29	30	36	20	12	29	65	55	61	268		
CDK200 TAD	19	21	27	15	8	24	37	42	48	115	141	
CDK200 VP64	29	27	48	23	9	40	36	51	56	79	68	139

Fig. S16. A comparison of the global regulation induced by CDKN1C-targeting zinc finger arrays as RTF and when expressed as fusion to truncated activation domains. a) For the CDK125, 150, 172, and 200 zinc finger arrays we expressed these as KLF6 RTFs (FL) as well as fusions to either the truncated KLF6 transactivation domain (TAD) as defined[17] or VP64. RNA-seq results are shown. **b**) PCA of RNA-seq results demonstrates that regulated genes mostly cluster by the zinc finger arrays employed, not the mode of activation. **c**) comparison of common regulated genes shows again that most off-target regulation clusters by which zinc fingers are employed.

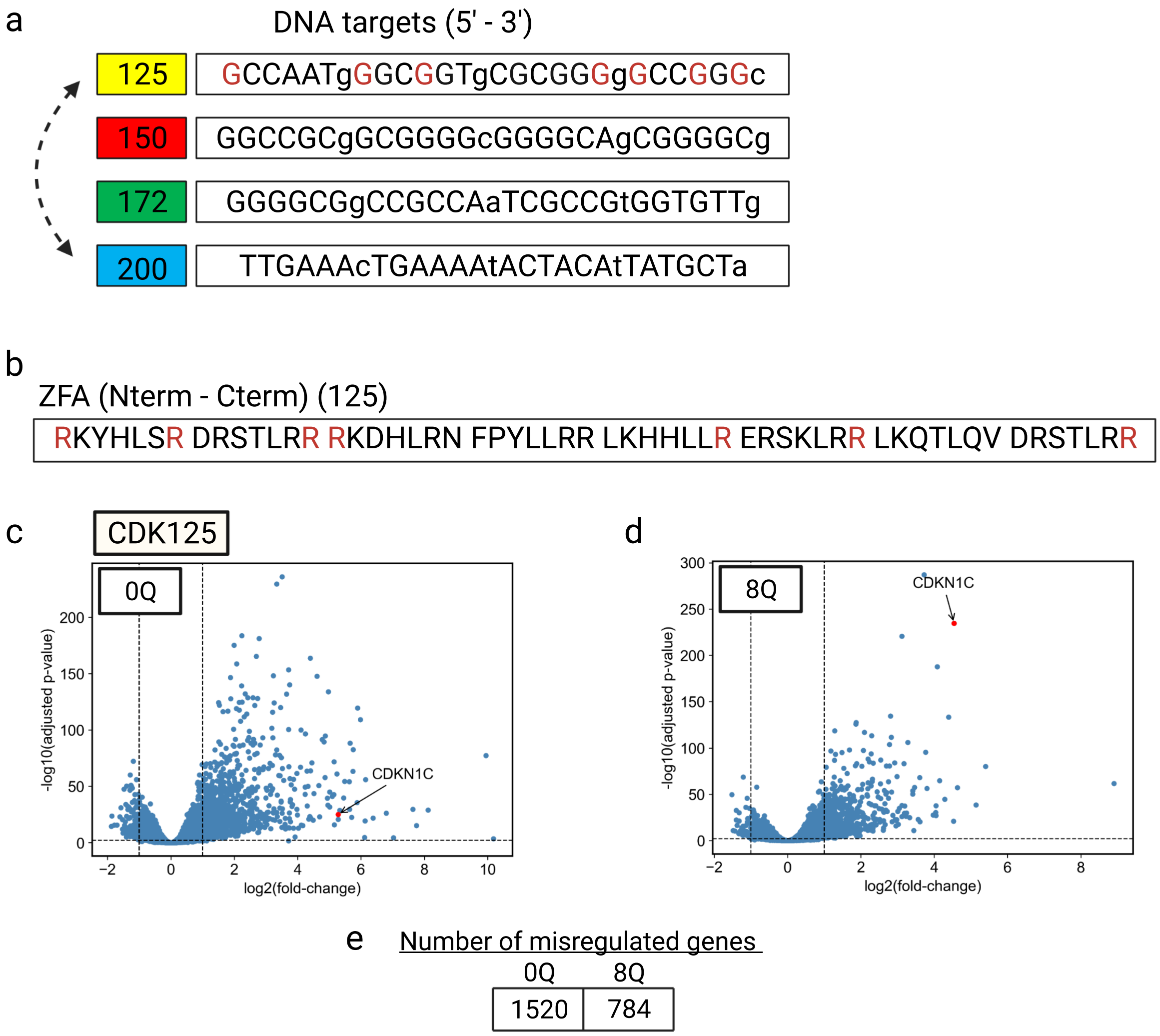

Fig. S17. The influence of target G-content and nonspecific affinity. a) The DNA targets for the 4 best arrays designed to activate CDKN1C are shown with CDK200 demonstrating the lowest G-count. **b**) The helices used by the most promiscuous CDK125 are shown with the position -1 and 6 arginines in red. These are designed to bind guanine and likely prefer guanine. However, arginines at these helical positions are also able to bind *any* base at their target positions which likely contributes to the high degree of off-target regulation with arrays designed to bind these G-rich targets. **c**) RNA-seq results for CDK125 without phosphate modifications. **d**) RNA-seq results for CDK125 with 8 phosphate contacts modified. **e**) Table of misregulated genes demonstrates that, despite the G-rich target for CDK125, nearly half of the misregulated genes are lost by reducing the nonspecific affinity.

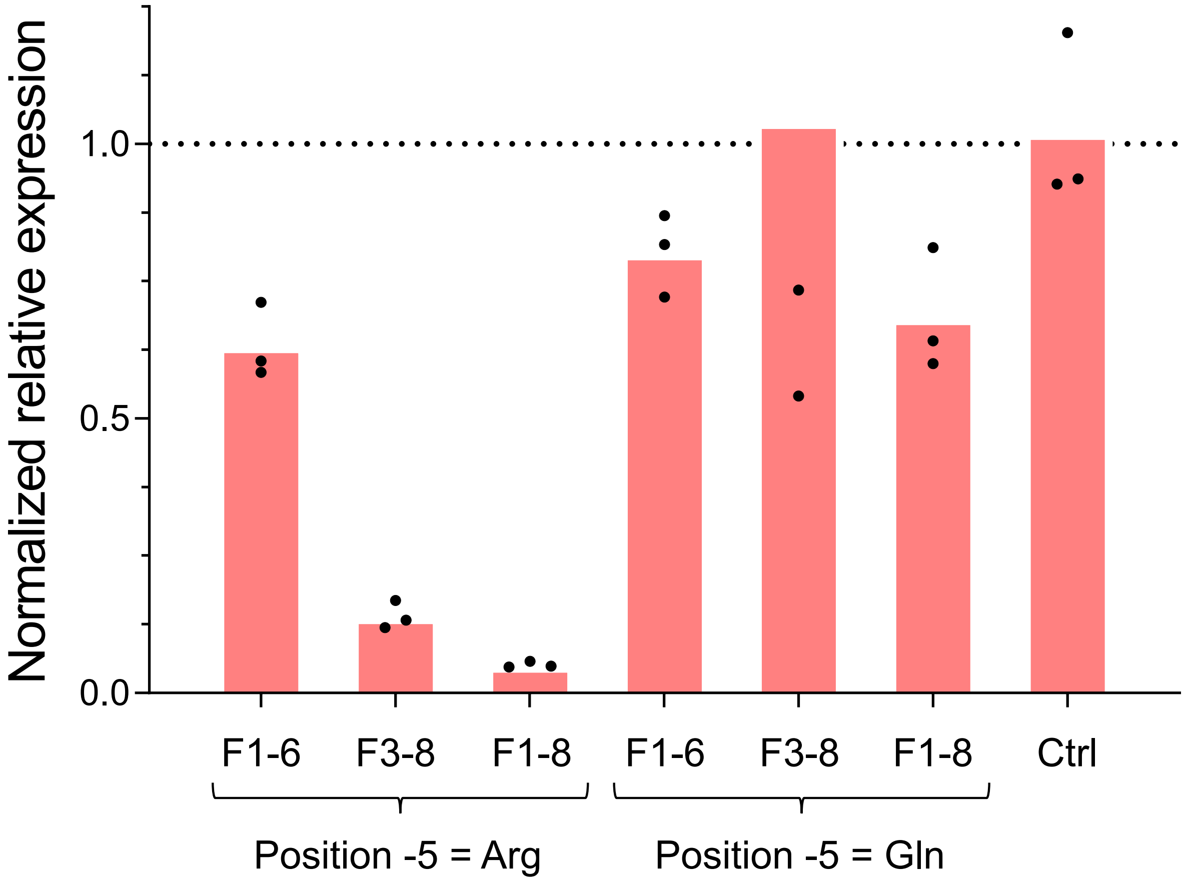

Fig. S18. The influence of substitutions of phosphate-contacting residues for the DPH1 array #15. Glutamine substitutions at the -5 phosphate contacting position as a negative impact on activity for the full 8-fingered protein as well as with N and C-terminal truncations.

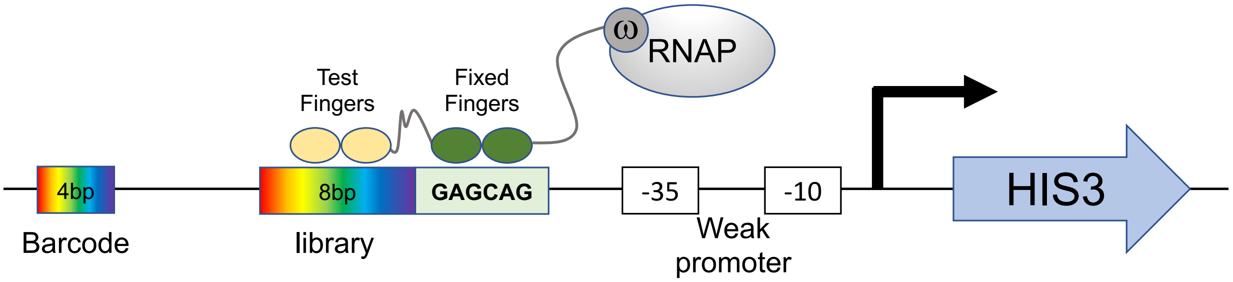

FQCRICMQNFSRKGNLKSHIRTHTGEKPFACDICGQKFARSANLTRHTKIHTQRPQIPPKP
FACDICGRKFAXXXXLXXHTRIHTGEKPFACDICGQRFAXXXXLXXHTKIHTQR

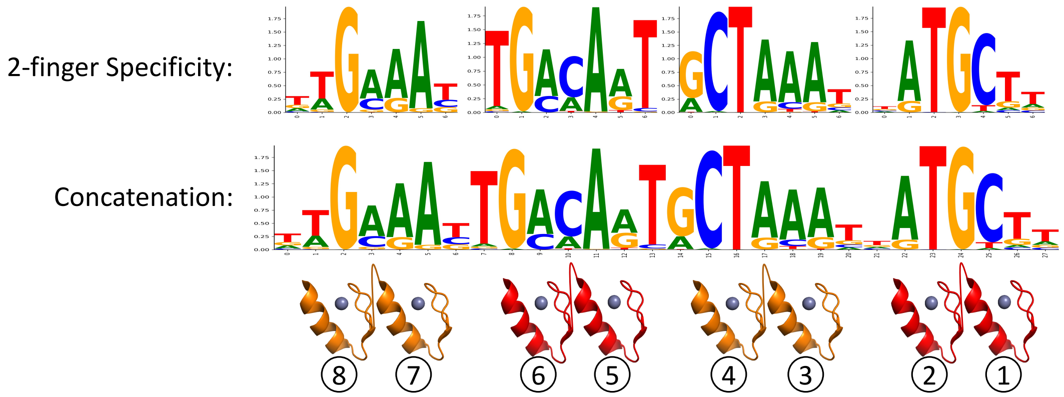

Fig. S19. Bacterial one-hybrid 8bp library to characterize the specificity of 2-finger modules. Top) A reporter vector was design as previously described[18] to make survival of the bacteria dependent on activation of HIS3 and therefore dependent on a compatible protein-DNA interaction. An 8bp region of random DNA sequences was placed upstream of a sequenced that fixed fingers in an array are known to bind (GAGCAG, green). Binding of the fixed fingers would position the test fingers (yellow) so that they can sample sequences in the library. If the test fingers and library sequence are compatible, HIS3 is activated and that cell survives the selective conditions. Note that the extended, base skipping linker is used between the fixed and test fingers so the test fingers will bind independently. In addition, to bias activity towards a functional test pair – DNA interaction, the position -5 residues of the fixed fingers have been mutated from Arginine to Glutamine (red) to decrease the affinity. **Bottom)** The specificities as characterized by this B1H assay for the CDKN1C #200 array are shown as directly produced by this assay and as a concatenation of all 8-fingers binding.

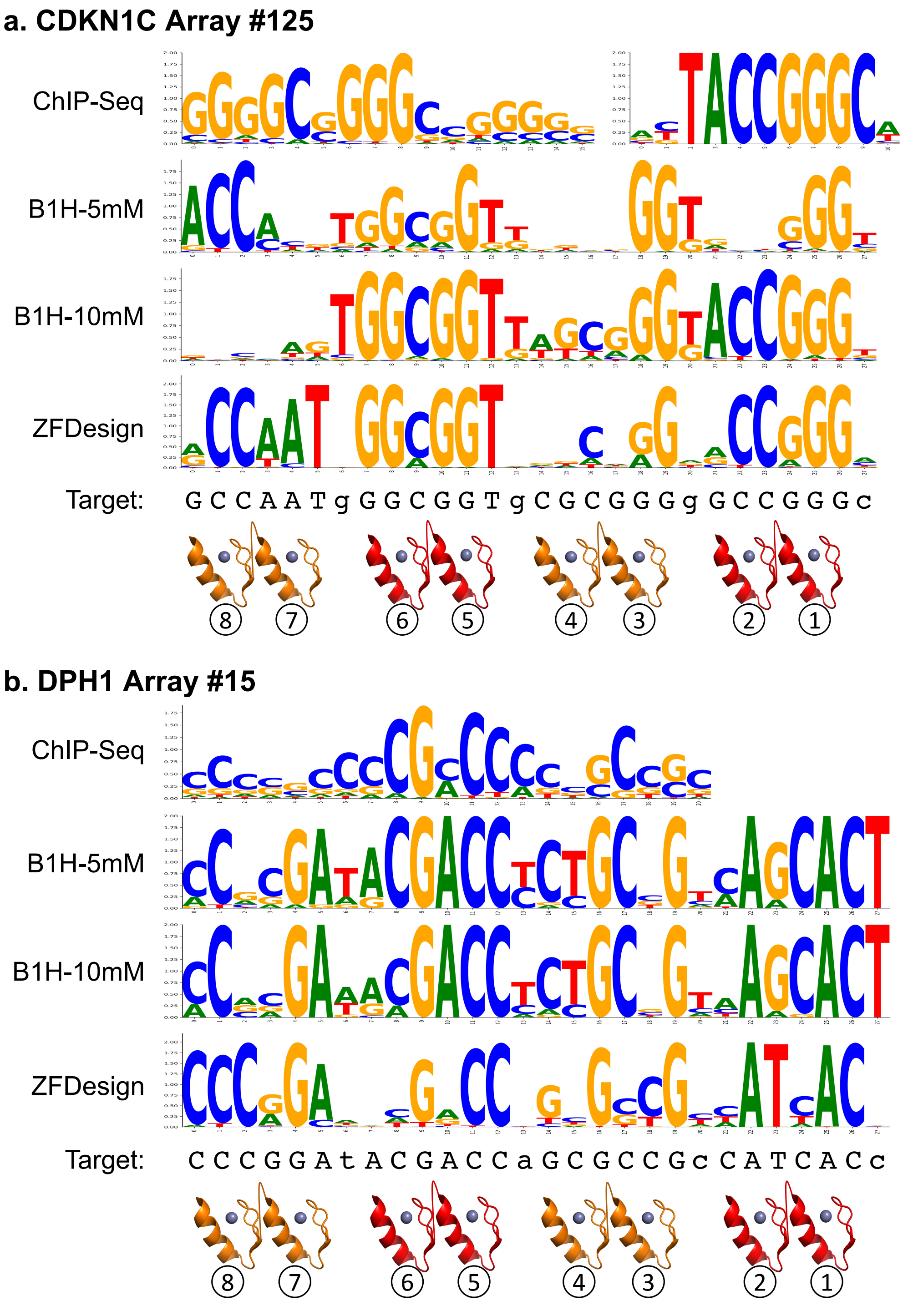

Fig. S20. Zinc finger arrays specificity a) The specificity of the CDKN1C #125 array as determined by ChIP-Seq, B1H selection at low (5mM) and high (10mM) stringency, and the ZFDesign predicted specificity. ChIP-seq returned two unique logos likely driven by different sets of ZFs. The B1H Logos are a concatenation of specificity determined independently for each of the 4 pairs of ZFs in the array. **b**) The specificity of the DPH1 #15 array as determined by ChIP-Seq, B1H selection at low (5mM) and high (10mM) stringency, and the ZFDesign predicted specificity.

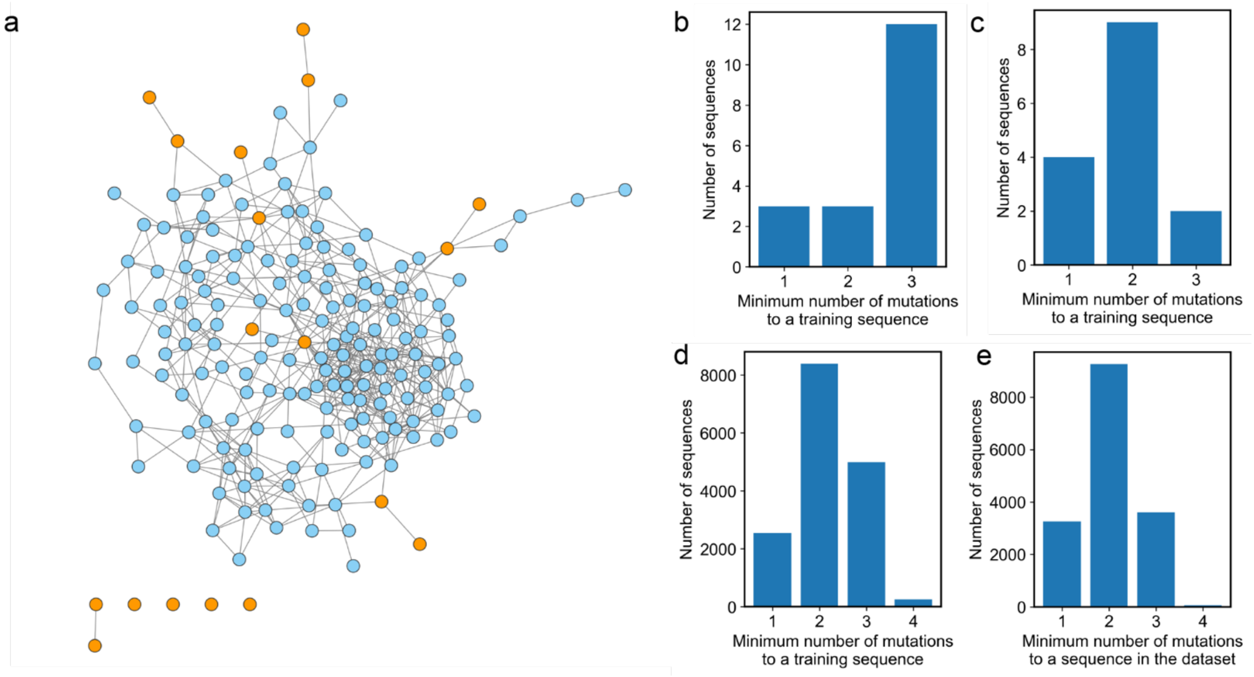

Fig. S21. Distribution of target sequences in the training and validation datasets. **a)** Graph representation of the seven-mer sequences in the training and validation datasets. Nodes represent seven-mers and edges connect nodes representing sequences within two substitutions of each other. Orange nodes are validation set sequences; blue nodes are training set sequences. **b)** Distances of validation set sequences to training set sequences. **c)** Distances of test set sequences to training set sequences. **d)** Distances of all seven-mer sequences to training set sequences. **e)** Distances of all seven-mer sequences to all sequences against which selections were performed.

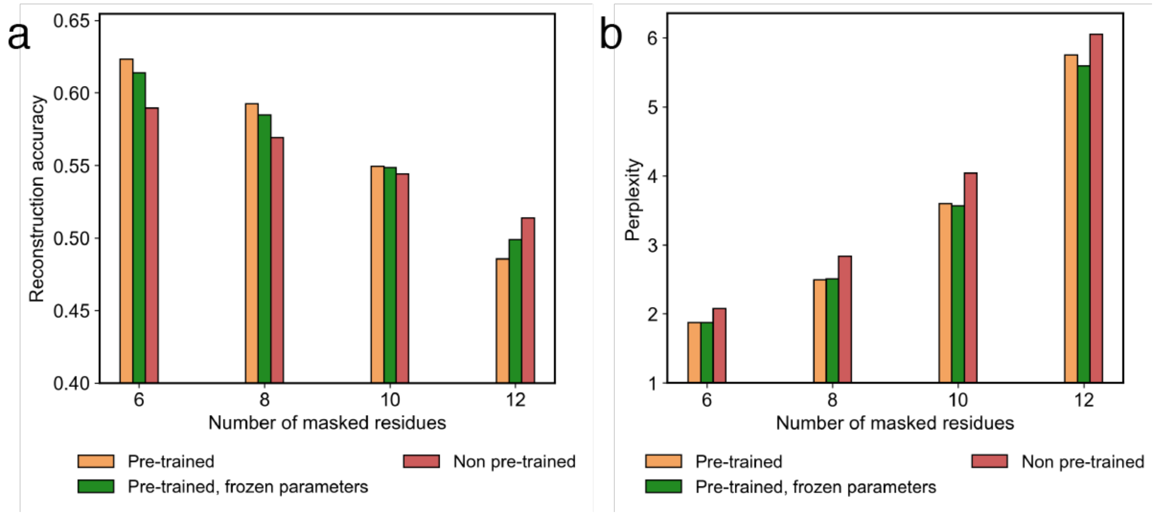

Fig. S22. Quantification of the effect of pre-training on model performance. a) Comparison of reconstruction accuracies when the model is pre-trained on single-helix selections and re-trained, re-trained with parameters of the single-helix modules frozen, and not pre-trained. **b)** Comparison of the perplexities when the model is pre-trained on single-helix selections and re-trained, re-trained with parameters of the single-helix modules frozen, and not pre-trained.

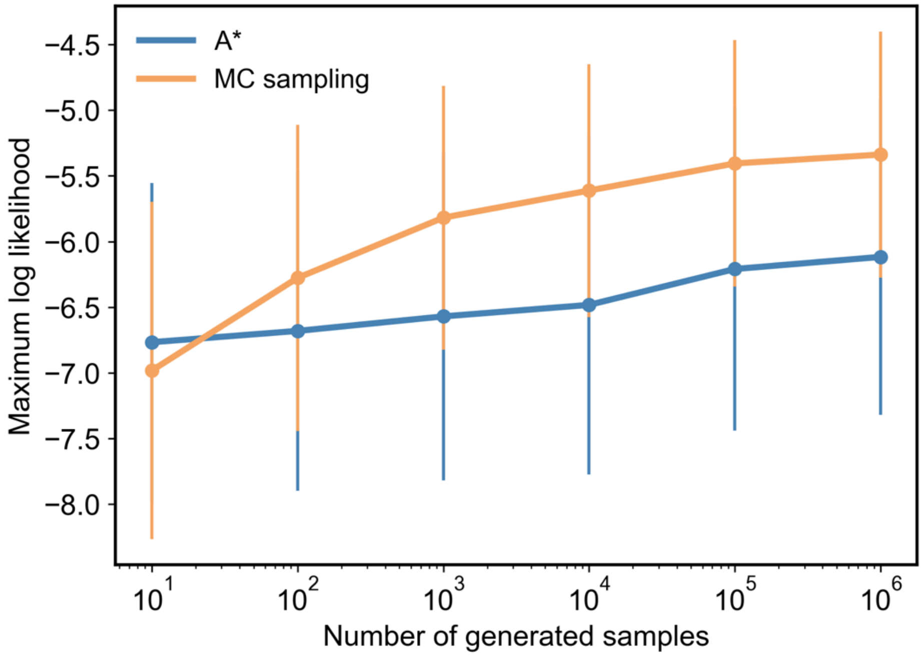

Fig. S23. Impact of number of generated samples on maximum likelihood design using A* or temperature dependent sampling. Error bars show the standard deviation (n=18).

Samples	Number of peaks within 500 bp of a transcript	Number of differentially expressed transcripts	Overlap
CDK1NC #200 8R	1791	110	29
CDK1NC #200 8Q	328	2	2
CDK1NC #125 8R	20171	798	756
CDK1NC #125 8Q	11135	458	430
Dph015 #15 8R	6157	7	3

Fig. S24. Comparison of ChIP-seq peaks with number of differentially expressed transcripts. Only a small fraction of the peaks returned by ChIP-seq result in a change in expression of a transcript suggesting the position, and potentially affinity of the protein-DNA interaction, play significant roles in regulation.

References

1 Lambert, S. A. *et al.* The Human Transcription Factors. *Cell* **175**, 598-599, doi:10.1016/j.cell.2018.09.045 (2018).

2 Jinek, M. *et al.* A programmable dual-RNA-guided DNA endonuclease in adaptive bacterial immunity. *Science* **337**, 816-821, doi:10.1126/science.1225829 (2012).

3 Elrod-Erickson, M., Rould, M. A., Nekludova, L. & Pabo, C. O. Zif268 protein-DNA complex refined at 1.6 A: a model system for understanding zinc finger-DNA interactions. *Structure* **4**, 1171-1180, doi:10.1016/s0969-2126(96)00125-6 (1996).

4 Rebar, E. J. & Pabo, C. O. Zinc finger phage: affinity selection of fingers with new DNA-binding specificities. *Science* **263**, 671-673, doi:10.1126/science.8303274 (1994).

5 Greisman, H. A. & Pabo, C. O. A general strategy for selecting high-affinity zinc finger proteins for diverse DNA target sites. *Science* **275**, 657-661, doi:10.1126/science.275.5300.657 (1997).

6 Maeder, M. L. *et al.* Rapid "open-source" engineering of customized zinc-finger nucleases for highly efficient gene modification. *Mol Cell* **31**, 294-301, doi:10.1016/j.molcel.2008.06.016 (2008).

7 Segal, D. J., Dreier, B., Beerli, R. R. & Barbas, C. F., 3rd. Toward controlling gene expression at will: selection and design of zinc finger domains recognizing each of the 5'-GNN-3' DNA target sequences. *Proc Natl Acad Sci U S A* **96**, 2758-2763, doi:10.1073/pnas.96.6.2758 (1999).

8 Persikov, A. V. *et al.* A systematic survey of the Cys2His2 zinc finger DNA-binding landscape. *Nucleic Acids Res* **43**, 1965-1984, doi:10.1093/nar/gku1395 (2015).

9 Choo, Y. & Klug, A. Toward a code for the interactions of zinc fingers with DNA: selection of randomized fingers displayed on phage. *Proc Natl Acad Sci U S A* **91**, 11163-11167, doi:10.1073/pnas.91.23.11163 (1994).

10 Dreier, B., Beerli, R. R., Segal, D. J., Flippin, J. D. & Barbas, C. F., 3rd. Development of zinc finger domains for recognition of the 5'-ANN-3' family of DNA sequences and their use in the construction of artificial transcription factors. *J Biol Chem* **276**, 29466-29478, doi:10.1074/jbc.M102604200 (2001).

11 Dreier, B. *et al.* Development of zinc finger domains for recognition of the 5'-CNN-3' family DNA sequences and their use in the construction of artificial transcription factors. *J Biol Chem* **280**, 35588-35597, doi:10.1074/jbc.M506654200 (2005).

12 Gupta, A. *et al.* An optimized two-finger archive for ZFN-mediated gene targeting. *Nat Methods* **9**, 588-590, doi:10.1038/nmeth.1994 (2012).

13 Zhu, C. *et al.* Using defined finger-finger interfaces as units of assembly for constructing zinc-finger nucleases. *Nucleic Acids Res* **41**, 2455-2465, doi:10.1093/nar/gks1357 (2013).

14 Isalan, M., Klug, A. & Choo, Y. A rapid, generally applicable method to engineer zinc fingers illustrated by targeting the HIV-1 promoter. *Nat Biotechnol* **19**, 656-660, doi:10.1038/90264 (2001).

15 Isalan, M., Klug, A. & Choo, Y. Comprehensive DNA recognition through concerted interactions from adjacent zinc fingers. *Biochemistry* **37**, 12026-12033, doi:10.1021/bi981358z (1998).
16 Reynolds, L. *et al.* Repression of the HIV-1 5' LTR promoter and inhibition of HIV-1 replication by using engineered zinc-finger transcription factors. *Proc Natl Acad Sci U S A* **100**, 1615-1620, doi:10.1073/pnas.252770699 (2003).
17 Alerasool, N., Leng, H., Lin, Z. Y., Gingras, A. C. & Taipale, M. Identification and functional characterization of transcriptional activators in human cells. *Mol Cell* **82**, 677-695 e677, doi:10.1016/j.molcel.2021.12.008 (2022).
18 Noyes, M. B. *et al.* A systematic characterization of factors that regulate Drosophila segmentation via a bacterial one-hybrid system. *Nucleic Acids Res* **36**, 2547-2560, doi:10.1093/nar/gkn048 (2008).

FORM OF NYU NON-COMMERCIAL RESEARCH LICENSE

Copyright 2022 New York University. All Rights Reserved.

A license to use and copy this data solely for your internal non-commercial research and evaluation purposes, without fee and without a signed licensing agreement, is hereby granted upon your download of the data, through which you agree to the following: 1) the above copyright notice, this paragraph and the following three paragraphs will prominently appear in all internal copies and modifications; 2) no rights to sublicense or further distribute this data are granted; 3) no rights to modify this data are granted; and 4) no rights to assign this license are granted. Please contact Sadhana Chitale (sadhana.chitale@nyulangone.org) at the NYU Technology, Opportunities & Venture Office for commercial licensing opportunities, or for further distribution, modification or license rights.

Created by Marcus Noyes.

IN NO EVENT SHALL NYU, OR THEIR EMPLOYEES, OFFICERS, AGENTS OR TRUSTEES ("COLLECTIVELY "NYU PARTIES") BE LIABLE TO ANY PARTY FOR DIRECT, INDIRECT, SPECIAL, INCIDENTAL, OR CONSEQUENTIAL DAMAGES OF ANY KIND, INCLUDING LOST PROFITS, ARISING OUT OF ANY CLAIM RESULTING FROM YOUR USE OF THIS DATA, EVEN IF ANY OF NYU PARTIES HAS BEEN ADVISED OF THE POSSIBILITY OF SUCH CLAIM OR DAMAGE.

NYU SPECIFICALLY DISCLAIMS ANY WARRANTIES OF ANY KIND REGARDING THE DATA, INCLUDING, BUT NOT LIMITED TO, NON-INFRINGEMENT, THE IMPLIED WARRANTIES OF MERCHANTABILITY AND FITNESS FOR A PARTICULAR PURPOSE, OR THE ACCURACY OR USEFULNESS, OR COMPLETENESS OF THE DATA. THE DATA AND ACCOMPANYING DOCUMENTATION, IF ANY, PROVIDED HEREUNDER IS PROVIDED COMPLETELY "AS IS". NYU HAS NO OBLIGATION TO PROVIDE FURTHER DOCUMENTATION, MAINTENANCE, SUPPORT, UPDATES, ENHANCEMENTS, OR MODIFICATIONS.

If you use this resource, cite: ____

TECHNOLOGY TRANSFER AGREEMENT

This Agreement is made between

THE GOVERNING COUNCIL OF THE UNIVERSITY OF TORONTO
(the **“Provider”**)

- and -

< Insert full legal name of individual or corporation >
(the **“Recipient”**)

effective as of the last date of signature below (the “**Effective Date**”).

Background

This Agreement sets out the understanding of the parties with respect to the provision of certain Technology created by researchers at the Provider to the Recipient, which wishes to use the Technology for non-commercial research purposes subject to the terms and conditions of this Agreement.

1.0 Definitions

The following words have the following meanings in this Agreement:

1. **Provider Scientist: Prof.**

2. **Recipient Scientist: Prof.**

3. **Technology:** means P2280/10004114: Seamless integration of Engineered Zinc Fingers into Endogenous Transcription Factors to Commandeer their Natural Functions P2254/10004093: A General Method to Design Zinc Finger Arrays to Specifically Target Any Specific DNA Sequence developed by Provider Scientist.

4. **Modifications:** Substances created by the Recipient which contain/incorporate the Technology.

5. **Commercial Purposes:** The sale, lease, license, or other transfer of the Technology or Modifications to a for-profit organization. Commercial Purposes shall also include uses of the Technology or Modifications by any organization, including Recipient, to perform contract research, to screen compound libraries, to produce or manufacture products for general sale, or to conduct research activities that result in any sale, lease, license, or transfer of the Technology or Modifications to a for-profit organization. However, industrially sponsored academic research shall not be considered a use of the Technology or Modifications for Commercial Purposes per se unless any of the above conditions of this definition are met.

6. **Nonprofit Organization(s):** A university or other institution of higher education or an organization of the type described in section 501(c)(3) of the U.S. Internal Revenue Code of 1954 (26 U.S.C. 501(c)) and exempt from taxation under section 501(a) of the Internal Revenue Code (26 U.S.C. 501(a)) or any nonprofit scientific or educational organization qualified under a state or provincial, as applicable, nonprofit organization statute. As used herein, the term also includes government agencies.

2.0 Technology Transfer

1. The Provider retains ownership of the Technology, including any Technology contained or incorporated in Modifications.

2. The Recipient and the Recipient Scientist agree that the Technology: (a) is to be used solely for teaching and academic research purposes; (b) will not be used in human subjects, in clinical trials, or for diagnostic purposes involving human subjects without the written consent of the Provider; (c) is to be used only at the Recipient organization and only in the Recipient Scientist's laboratory under the direction of the Recipient Scientist or others working under his/her direct supervision; and (d) will not be transferred to anyone else within the Recipient organization without the prior written consent of the Provider.

3. The Recipient and the Recipient Scientist agree to refer to the Provider any request for the Technology from anyone other than those persons working under the Recipient Scientist's direct supervision. To the extent supplies are available, the Provider or the Recipient Scientist agrees to make the Technology available, under a separate Agreement, to other scientists (at least those at Nonprofit Organization(s)) who wish to replicate the Recipient Scientist's research; provided that such other scientists reimburse the Provider for any costs relating to the preparation and distribution of the Technology.

4. Recipient Rights

   (a) The Recipient and/or the Recipient Scientist shall have the right, without restriction, to distribute results created by the Recipient through the use of the Technology only if those substances are not Modifications.

   (b) Under a separate Agreement (or an agreement at least as protective of the Provider's rights), the Recipient may distribute Modifications to Nonprofit Organization(s) for research and teaching purposes only.

   (c) Without written consent from the Provider, the Recipient and/or the Recipient Scientist may NOT provide Modifications for Commercial Purposes. It is recognized by the Recipient that such Commercial Purposes may require a commercial license from the Provider and the Provider has no obligation to grant a commercial license to its ownership interest in the Technology incorporated in the Modifications. Subject to Section 4 (a), nothing in this paragraph, however, shall prevent the Recipient from granting commercial licenses under the Recipient's intellectual property rights in results generated by the Recipient, or methods of their manufacture or their use.

5. The Recipient acknowledges that the Technology is or may be the subject of a patent application. Except as provided in this Agreement, no express or implied licenses or other rights are provided to the Recipient under any patents, patent applications, trade secrets or other proprietary rights of the Provider, including any altered

forms of the Technology made by the Provider. In particular, no express or implied licenses or other rights are provided to use the Technology, Modifications, or any related patents of the Provider for Commercial Purposes.

6. If the Recipient desires to use or license the Technology or Modifications for Commercial Purposes, the Recipient agrees, in advance of such use, to negotiate in good faith with the Provider to establish the terms of a commercial license. It is understood by the Recipient that the Provider shall have no obligation to grant such a license to the Recipient, and may grant exclusive or non-exclusive commercial licenses to others, or sell or assign all or part of the rights in the Technology to any third party(ies), subject to any pre-existing rights held by others and obligations to the Federal Government.

7. The Recipient is free to file patent application(s) claiming inventions made by the Recipient through the use of the Technology but agrees to notify the Provider upon filing a patent application claiming Modifications or method(s) of manufacture or use(s) of the Technology.

8. Any Technology delivered pursuant to this Agreement is understood to be experimental in nature and may have hazardous properties. The Provider makes no representations and extends no warranties of any kind, either expressed or implied. There are no express or implied warranties of merchantability or fitness for particular purpose, or that the use of Technology will not infringe any patent, copyright, trademark, or other proprietary rights.

9. Except to the extent prohibited by law, the Recipient assumes all liability for damages which may arise from its use, storage or disposal of the Technology. The Provider will not be liable to the Recipient for any loss, claim or demand made by the Recipient, or made against the Recipient by any other party, due to or arising from the use of the Technology by the Recipient, except to the extent permitted by law when caused by the gross negligence or willful misconduct of the Provider.

10. This agreement shall not be interpreted to prevent or delay publication of research findings resulting from the use of the Technology or the Modifications. The Recipient will provide a copy of any proposed publication of Project research results (a **"Publication"**) to the Provider for its review at least sixty (60) days before submission for publication or disclosure. Upon the Provider's written request received within sixty (60) days of the Provider's receipt of the Publication, the Recipient will, at the Provider's option delete identifiable references to any confidential information provided by the Provider from the proposed Publication. The Recipient Scientist agrees to provide appropriate acknowledgment of the source of the Technology in all publications.

11. The Recipient agrees to use the Technology in compliance with all applicable statutes and regulations.

12. This Agreement will terminate on the earliest of the following dates: (a) when the Technology becomes generally available from third parties, or (b) on completion of the Recipient's current research with the Technology, or (c) on thirty (30) days written notice by either party to the other, or (d) on the date specified in an implementing letter, provided that:

    i. if termination should occur under Section 12(a), the Recipient shall be bound to the Provider by the least restrictive terms applicable to the Technology obtained from the then-available sources; and

ii. if termination should occur under 12(b) or (d) above, the Recipient will discontinue its use of the Technology and will, upon direction of the Provider, return or destroy any remaining Technology. The Recipient, at its discretion, will also either destroy the Modifications or remain bound by the terms of this agreement as they apply to Modifications; and

iii. in the event the Provider terminates this Agreement under 12(c) other than for breach of this Agreement or for cause such as an imminent health risk or patent infringement, the Provider will defer the effective date of termination for a period of up to one year, upon request from the Recipient, to permit completion of research in progress. Upon the effective date of termination, or if requested, the deferred effective date of termination, Recipient will discontinue its use of the Technology and will, upon direction of the Provider, return or destroy any remaining Technology. The Recipient, at its discretion, will also either destroy the Modifications or remain bound by the terms of this agreement as they apply to Modifications.

13. The Technology is provided at no cost, or with an optional transmittal fee solely to reimburse the Provider for its preparation and distribution costs. If a fee is requested by the Provider, the amount will be indicated in an implementing letter.

3.0 Miscellaneous

1. **Notices.** Communication between the parties shall be given in writing and may be given by personal delivery, express delivery service, certified or registered mail, postage prepaid, or facsimile transmission, addressed to:

(a) if to the **Provider**

	For Legal and Administrative Matters:	*For Technical and Scientific Matters:*
**Name:**	Tina Coccia<br>Director, Partnerships	Philip Kim<br>Professor
**Department:**	Innovations & Partnership Office	The Donnelly Centre for Cellular and Biomolecular Research<br>Department of Molecular Genetics<br>Department of Computer Science
**Address:**	Banting Institute 100 College Street, Suite 413	160 College Street, Rm 616
**City, Province/State:**	Toronto, Ontario	Toronto, Ontario
**Postal/Zip Code, Country:**	M5G 1L5, Canada	M5S 3E1, Canada
**Tel:**	416-978- 5557	416-946-3419
**Email:**	innovations.partnerships@utoronto.ca	pm.kim@utoronto.ca

(b) if to the **Recipient**

	For Legal and Administrative Matters:	*For Technical and Scientific Matters:*
**Name:**	< Insert >	< Insert >
**Department:**	< Insert >	< Insert >
**Address:**	< Insert >	< Insert >
**City, Province/State:**	< Insert >	< Insert >
**Postal/Zip Code, Country:**	< Insert >	< Insert >
**Tel:**	< Insert >	< Insert >
**Email:**	< Insert >	< Insert >

2. **Survival.** Sections 2: 6, 9, 10, Section 3: 2, 3, 4, 5 and 6 shall survive termination.

3. **No Assignment.** The **Recipient** shall not assign any or all of its rights and obligations under this Agreement without the University's prior written consent, which may not be unreasonably withheld.

4. **Successors.** This Agreement will bind and enure to the benefit of the parties and their respective heirs, successors and permitted assigns.

5. **Entire Agreement.** This Agreement is the entire agreement of the parties and no change or modification will be valid unless it is in writing and signed by all parties.

6. **Governing Law.** This Agreement shall be governed by and construed in accordance with the laws of the Province of Ontario, without reference to its conflicts of laws provisions.

7. **Headings.** Paragraph headings in this Agreement are for purposes of convenience only and will not be used in the interpretation of this Agreement.

IN WITNESS WHEREOF by signature of their respective authorized officers, the parties agree to be bound by the terms of this Agreement.

THE GOVERNING COUNCIL OF THE UNIVERSITY OF TORONTO

NAME: Tina Coccia

TITLE: Director, Partnerships

DATE:

< Insert full legal name of individual or corporation >

NAME:

TITLE:

DATE:

Recipient Researcher:

Having read this Agreement, I hereby agree to act in accordance with all the terms and conditions herein and applicable University of Toronto policies, and, if applicable, further agree to inform all participants of their obligations under such terms and conditions.

NAME:

DATE:
