# Supplementary material for: A universal deep-learning model for zinc finger design enables transcription factor reprogramming
Source: Nat Biotechnol. 2023 Jan 26;41(8):1117–29. doi: 10.1038/s41587-022-01624-4 (PMC10421740; doi:10.1038/s41587-022-01624-4)
Supplement: Supplementary file 3 — Reporting Summary [file 41587_2022_1624_MOESM3_ESM.pdf]

nature research

Corresponding author(s): Marcus Noyes, PhD

Last updated by author(s): Nov 9, 2022

# Reporting Summary

Nature Research wishes to improve the reproducibility of the work that we publish. This form provides structure for consistency and transparency in reporting. For further information on Nature Research policies, see our Editorial Policies and the Editorial Policy Checklist.

## Statistics

For all statistical analyses, confirm that the following items are present in the figure legend, table legend, main text, or Methods section.

| n/a | Confirmed | |
|---|---|---|
| ☐ | ☒ | The exact sample size (*n*) for each experimental group/condition, given as a discrete number and unit of measurement |
| ☐ | ☒ | A statement on whether measurements were taken from distinct samples or whether the same sample was measured repeatedly |
| ☐ | ☒ | The statistical test(s) used AND whether they are one- or two-sided<br>*Only common tests should be described solely by name; describe more complex techniques in the Methods section.* |
| ☐ | ☒ | A description of all covariates tested |
| ☐ | ☒ | A description of any assumptions or corrections, such as tests of normality and adjustment for multiple comparisons |
| ☐ | ☒ | A full description of the statistical parameters including central tendency (e.g. means) or other basic estimates (e.g. regression coefficient) AND variation (e.g. standard deviation) or associated estimates of uncertainty (e.g. confidence intervals) |
| ☐ | ☒ | For null hypothesis testing, the test statistic (e.g. *F*, *t*, *r*) with confidence intervals, effect sizes, degrees of freedom and *P* value noted<br>*Give P values as exact values whenever suitable.* |
| ☐ | ☒ | For Bayesian analysis, information on the choice of priors and Markov chain Monte Carlo settings |
| ☐ | ☒ | For hierarchical and complex designs, identification of the appropriate level for tests and full reporting of outcomes |
| ☒ | ☐ | Estimates of effect sizes (e.g. Cohen's *d*, Pearson's *r*), indicating how they were calculated |

*Our web collection on statistics for biologists contains articles on many of the points above.*

## Software and code

Policy information about availability of computer code

| | |
|---|---|
| Data collection | SH800 software version 2.1.6 was used to collect FACS data. |
| Data analysis | RNA-Seq analysis was done using STAR aligner version 2.7.8a, FeatureCounts from subread version 2.0.1, and DESeq2 version 1.30.1. Python version 3.8.3 was used with pytorch version 1.9.0, numpy version 1.21.0, scipy version 1.7.0, biopython version 1.78, pandas version 1.2.5, and matplotlib version 3.4.1. |

For manuscripts utilizing custom algorithms or software that are central to the research but not yet described in published literature, software must be made available to editors and reviewers. We strongly encourage code deposition in a community repository (e.g. GitHub). See the Nature Research guidelines for submitting code & software for further information.

## Data

Policy information about availability of data

All manuscripts must include a data availability statement. This statement should provide the following information, where applicable:

- Accession codes, unique identifiers, or web links for publicly available datasets
- A list of figures that have associated raw data
- A description of any restrictions on data availability

The Data generated to produce the ZF design model is proprietary. However, for the purposes of reproducibility, and to encourage others to use designer ZFs in their work, the design model ZFDesign will be available to the academic community with an MTA. All constructs described in this manuscript are available upon request and base cloning constructs such as the KLF6 and Zim3 scaffolds will be deposited with addgene.

# Field-specific reporting

Please select the one below that is the best fit for your research. If you are not sure, read the appropriate sections before making your selection.

☒ Life sciences ☐ Behavioural & social sciences ☐ Ecological, evolutionary & environmental sciences

For a reference copy of the document with all sections, see nature.com/documents/nr-reporting-summary-flat.pdf

# Life sciences study design

All studies must disclose on these points even when the disclosure is negative.

| | |
|---|---|
| Sample size | The nature of this work is exploratory screening, and as such sample sizes were determined by what was a feasible number of constructs to test given the lab size. |
| Data exclusions | No data other than those collected during assay development (i.e. troubleshooting technical difficulties) were excluded. The reported data were collected according to the finalized protocols detailed in the methods. |
| Replication | Large scale screens were performed once as it would be infeasible to perform true replicates, however the degenerate NNS coding scheme allows the simultaneous assessment of functionally identical ZF helices. Only ZFs encoded by more than one DNA sequence were considered for analysis. Transcriptional activation/repression and ZFN assays contain two or three biological replicates, which typically showed good agreement (all data are plotted). |
| Randomization | This is not relevant to our study as there were no groups to assign. |
| Blinding | Blinding was not performed since the data is quantitative and therefore statistical differences are clear. |

# Behavioural & social sciences study design

All studies must disclose on these points even when the disclosure is negative.

| | |
|---|---|
| Study description | *Briefly describe the study type including whether data are quantitative, qualitative, or mixed-methods (e.g. qualitative cross-sectional, quantitative experimental, mixed-methods case study).* |
| Research sample | *State the research sample (e.g. Harvard university undergraduates, villagers in rural India) and provide relevant demographic information (e.g. age, sex) and indicate whether the sample is representative. Provide a rationale for the study sample chosen. For studies involving existing datasets, please describe the dataset and source.* |
| Sampling strategy | *Describe the sampling procedure (e.g. random, snowball, stratified, convenience). Describe the statistical methods that were used to predetermine sample size OR if no sample-size calculation was performed, describe how sample sizes were chosen and provide a rationale for why these sample sizes are sufficient. For qualitative data, please indicate whether data saturation was considered, and what criteria were used to decide that no further sampling was needed.* |
| Data collection | *Provide details about the data collection procedure, including the instruments or devices used to record the data (e.g. pen and paper, computer, eye tracker, video or audio equipment) whether anyone was present besides the participant(s) and the researcher, and whether the researcher was blind to experimental condition and/or the study hypothesis during data collection.* |
| Timing | *Indicate the start and stop dates of data collection. If there is a gap between collection periods, state the dates for each sample cohort.* |
| Data exclusions | *If no data were excluded from the analyses, state so OR if data were excluded, provide the exact number of exclusions and the rationale behind them, indicating whether exclusion criteria were pre-established.* |
| Non-participation | *State how many participants dropped out/declined participation and the reason(s) given OR provide response rate OR state that no participants dropped out/declined participation.* |
| Randomization | *If participants were not allocated into experimental groups, state so OR describe how participants were allocated to groups, and if allocation was not random, describe how covariates were controlled.* |

# Ecological, evolutionary & environmental sciences study design

All studies must disclose on these points even when the disclosure is negative.

| | |
|---|---|
| Study description | *Briefly describe the study. For quantitative data include treatment factors and interactions, design structure (e.g. factorial, nested, hierarchical), nature and number of experimental units and replicates.* |

| | |
|---|---|
| Research sample | *Describe the research sample (e.g. a group of tagged Passer domesticus, all Stenocereus thurberi within Organ Pipe Cactus National Monument), and provide a rationale for the sample choice. When relevant, describe the organism taxa, source, sex, age range and any manipulations. State what population the sample is meant to represent when applicable. For studies involving existing datasets, describe the data and its source.* |
| Sampling strategy | *Note the sampling procedure. Describe the statistical methods that were used to predetermine sample size OR if no sample-size calculation was performed, describe how sample sizes were chosen and provide a rationale for why these sample sizes are sufficient.* |
| Data collection | *Describe the data collection procedure, including who recorded the data and how.* |
| Timing and spatial scale | *Indicate the start and stop dates of data collection, noting the frequency and periodicity of sampling and providing a rationale for these choices. If there is a gap between collection periods, state the dates for each sample cohort. Specify the spatial scale from which the data are taken* |
| Data exclusions | *If no data were excluded from the analyses, state so OR if data were excluded, describe the exclusions and the rationale behind them, indicating whether exclusion criteria were pre-established.* |
| Reproducibility | *Describe the measures taken to verify the reproducibility of experimental findings. For each experiment, note whether any attempts to repeat the experiment failed OR state that all attempts to repeat the experiment were successful.* |
| Randomization | *Describe how samples/organisms/participants were allocated into groups. If allocation was not random, describe how covariates were controlled. If this is not relevant to your study, explain why.* |
| Blinding | *Describe the extent of blinding used during data acquisition and analysis. If blinding was not possible, describe why OR explain why blinding was not relevant to your study.* |

Did the study involve field work? ☐ Yes ☐ No

## Field work, collection and transport

| | |
|---|---|
| Field conditions | *Describe the study conditions for field work, providing relevant parameters (e.g. temperature, rainfall).* |
| Location | *State the location of the sampling or experiment, providing relevant parameters (e.g. latitude and longitude, elevation, water depth).* |
| Access & import/export | *Describe the efforts you have made to access habitats and to collect and import/export your samples in a responsible manner and in compliance with local, national and international laws, noting any permits that were obtained (give the name of the issuing authority, the date of issue, and any identifying information).* |
| Disturbance | *Describe any disturbance caused by the study and how it was minimized.* |

# Reporting for specific materials, systems and methods

We require information from authors about some types of materials, experimental systems and methods used in many studies. Here, indicate whether each material, system or method listed is relevant to your study. If you are not sure if a list item applies to your research, read the appropriate section before selecting a response.

### Materials & experimental systems

| n/a | Involved in the study | |
|---|---|---|
| ☒ | ☐ | Antibodies |
| ☐ | ☒ | Eukaryotic cell lines |
| ☒ | ☐ | Palaeontology and archaeology |
| ☒ | ☐ | Animals and other organisms |
| ☒ | ☐ | Human research participants |
| ☒ | ☐ | Clinical data |
| ☒ | ☐ | Dual use research of concern |

### Methods

| n/a | Involved in the study | |
|---|---|---|
| ☐ | ☒ | ChIP-seq |
| ☐ | ☒ | Flow cytometry |
| ☒ | ☐ | MRI-based neuroimaging |

## Antibodies

| | |
|---|---|
| Antibodies used | *Describe all antibodies used in the study; as applicable, provide supplier name, catalog number, clone name, and lot number.* |
| Validation | *Describe the validation of each primary antibody for the species and application, noting any validation statements on the manufacturer's website, relevant citations, antibody profiles in online databases, or data provided in the manuscript.* |

## Eukaryotic cell lines

Policy information about cell lines

| | |
|---|---|
| Cell line source(s) | The HEK293T line is available from ATCC: CRL-3216. The exact U2OS-GFP reporter line used in this is not known to be publicly available. It is referenced in several papers, and has been available to this lab for over a decade. references:<br>1) https://www.nature.com/articles/nbt.2170<br>2) https://www.nature.com/articles/nature16526<br>3) https://www.ncbi.nlm.nih.gov/pmc/articles/PMC3300077/. |
| Authentication | Cell lines were not authenticated since they were used solely as a platform to test zinc finger technologies. |
| Mycoplasma contamination | Cell lines were not routinely tested for mycoplasma. |
| Commonly misidentified lines (See ICLAC register) | No commonly misidentified cell lines were used in this study. |

## Palaeontology and Archaeology

| | |
|---|---|
| Specimen provenance | *Provide provenance information for specimens and describe permits that were obtained for the work (including the name of the issuing authority, the date of issue, and any identifying information).* |
| Specimen deposition | *Indicate where the specimens have been deposited to permit free access by other researchers.* |
| Dating methods | *If new dates are provided, describe how they were obtained (e.g. collection, storage, sample pretreatment and measurement), where they were obtained (i.e. lab name), the calibration program and the protocol for quality assurance OR state that no new dates are provided.* |

☐ Tick this box to confirm that the raw and calibrated dates are available in the paper or in Supplementary Information.

| | |
|---|---|
| Ethics oversight | *Identify the organization(s) that approved or provided guidance on the study protocol, OR state that no ethical approval or guidance was required and explain why not.* |

Note that full information on the approval of the study protocol must also be provided in the manuscript.

## Animals and other organisms

Policy information about studies involving animals; ARRIVE guidelines recommended for reporting animal research

| | |
|---|---|
| Laboratory animals | *For laboratory animals, report species, strain, sex and age OR state that the study did not involve laboratory animals.* |
| Wild animals | *Provide details on animals observed in or captured in the field; report species, sex and age where possible. Describe how animals were caught and transported and what happened to captive animals after the study (if killed, explain why and describe method; if released, say where and when) OR state that the study did not involve wild animals.* |
| Field-collected samples | *For laboratory work with field-collected samples, describe all relevant parameters such as housing, maintenance, temperature, photoperiod and end-of-experiment protocol OR state that the study did not involve samples collected from the field.* |
| Ethics oversight | *Identify the organization(s) that approved or provided guidance on the study protocol, OR state that no ethical approval or guidance was required and explain why not.* |

Note that full information on the approval of the study protocol must also be provided in the manuscript.

## Human research participants

Policy information about studies involving human research participants

| | |
|---|---|
| Population characteristics | *Describe the covariate-relevant population characteristics of the human research participants (e.g. age, gender, genotypic information, past and current diagnosis and treatment categories). If you filled out the behavioural & social sciences study design questions and have nothing to add here, write "See above."* |
| Recruitment | *Describe how participants were recruited. Outline any potential self-selection bias or other biases that may be present and how these are likely to impact results.* |
| Ethics oversight | *Identify the organization(s) that approved the study protocol.* |

Note that full information on the approval of the study protocol must also be provided in the manuscript.

## Clinical data

Policy information about clinical studies

All manuscripts should comply with the ICMJE guidelines for publication of clinical research and a completed CONSORT checklist must be included with all submissions.

| | |
|---|---|
| Clinical trial registration | *Provide the trial registration number from ClinicalTrials.gov or an equivalent agency.* |
| Study protocol | *Note where the full trial protocol can be accessed OR if not available, explain why.* |
| Data collection | *Describe the settings and locales of data collection, noting the time periods of recruitment and data collection.* |
| Outcomes | *Describe how you pre-defined primary and secondary outcome measures and how you assessed these measures.* |

## Dual use research of concern

Policy information about dual use research of concern

### Hazards

Could the accidental, deliberate or reckless misuse of agents or technologies generated in the work, or the application of information presented in the manuscript, pose a threat to:

| No | Yes | |
|---|---|---|
| ☐ | ☐ | Public health |
| ☐ | ☐ | National security |
| ☐ | ☐ | Crops and/or livestock |
| ☐ | ☐ | Ecosystems |
| ☐ | ☐ | Any other significant area |

### Experiments of concern

Does the work involve any of these experiments of concern:

| No | Yes | |
|---|---|---|
| ☐ | ☐ | Demonstrate how to render a vaccine ineffective |
| ☐ | ☐ | Confer resistance to therapeutically useful antibiotics or antiviral agents |
| ☐ | ☐ | Enhance the virulence of a pathogen or render a nonpathogen virulent |
| ☐ | ☐ | Increase transmissibility of a pathogen |
| ☐ | ☐ | Alter the host range of a pathogen |
| ☐ | ☐ | Enable evasion of diagnostic/detection modalities |
| ☐ | ☐ | Enable the weaponization of a biological agent or toxin |
| ☐ | ☐ | Any other potentially harmful combination of experiments and agents |

## ChIP-seq

### Data deposition

☒ Confirm that both raw and final processed data have been deposited in a public database such as GEO.

☒ Confirm that you have deposited or provided access to graph files (e.g. BED files) for the called peaks.

| | |
|---|---|
| Data access links<br>*May remain private before publication.* | https://www.ncbi.nlm.nih.gov/geo/query/acc.cgi?acc=GSE215330 |
| Files in database submission | 10_CDK200_8R_ChIP_2_S17_L001_R1_001.fastq.gz<br>10_CDK200_8R_ChIP_2_S17_L001_R2_001.fastq.gz<br>1_CDK125_8R_ChIP_1_S10_L001_R1_001.fastq.gz<br>1_CDK125_8R_ChIP_1_S10_L001_R2_001.fastq.gz<br>27_YY1_ChIP_1_S28_L001_R1_001.fastq.gz<br>27_YY1_ChIP_1_S28_L001_R2_001.fastq.gz<br>28_YY1_ChIP_2_S29_L001_R1_001.fastq.gz<br>28_YY1_ChIP_2_S29_L001_R2_001.fastq.gz<br>29_CDK125_8R_Input_1_S30_L001_R1_001.fastq.gz<br>29_CDK125_8R_Input_1_S30_L001_R2_001.fastq.gz<br>2_CDK125_8R_ChIP_2_S11_L001_R1_001.fastq.gz<br>2_CDK125_8R_ChIP_2_S11_L001_R2_001.fastq.gz |

| | |
|---|---|
| | 30_CDK125_8Q_Input_2_S31_L001_R1_001.fastq.gz<br>30_CDK125_8Q_Input_2_S31_L001_R2_001.fastq.gz<br>34_CDK200_8R_Input_1_S33_L001_R1_001.fastq.gz<br>34_CDK200_8R_Input_1_S33_L001_R2_001.fastq.gz<br>3_CDK125_8Q_ChIP_1_S12_L001_R1_001.fastq.gz<br>3_CDK125_8Q_ChIP_1_S12_L001_R2_001.fastq.gz<br>42_YY1_Input_2_S39_L001_R1_001.fastq.gz<br>42_YY1_Input_2_S39_L001_R2_001.fastq.gz<br>4_CDK125_8Q_ChIP_2_S13_L001_R1_001.fastq.gz<br>4_CDK125_8Q_ChIP_2_S13_L001_R2_001.fastq.gz<br>9_CDK200_8R_ChIP_1_S16_L001_R1_001.fastq.gz<br>9_CDK200_8R_ChIP_1_S16_L001_R2_001.fastq.gz<br>Dph015_8R_ChIP_1_S13_L002_R1_001.fastq.gz<br>Dph015_8R_ChIP_1_S13_L002_R2_001.fastq.gz<br>Dph015_8R_ChIP_2_S14_L002_R1_001.fastq.gz<br>Dph015_8R_ChIP_2_S14_L002_R2_001.fastq.gz<br>Dph015_8R_Input_1_S15_L002_R1_001.fastq.gz<br>Dph015_8R_Input_1_S15_L002_R2_001.fastq.gz<br>Dph015_8R_Input_2_S16_L002_R1_001.fastq.gz<br>Dph015_8R_Input_2_S16_L002_R2_001.fastq.gz<br>CDK125_8Q.bdg<br>CDK125_8R.bdg<br>CDK200_8R.bdg<br>Dph015_8R.bdg<br>YY1.bdg |
| Genome browser session (e.g. UCSC) | No longer applicable |

## Methodology

| | |
|---|---|
| Replicates | Two biological replicates were performed for each sample. They were sequences a long with the input DNA. The replicates showed good replicability |
| Sequencing depth | The reads were all paired-end. The following are the stats:<br>CDK125_8Q biol rep 1 30548650 reads 22974981 uniquely mapped<br>CDK125_8Q biol rep 2 26467519 reads 22803501 uniquely mapped<br>CDK125_8Q input DNA 25669459 reads 22207945 uniquely mapped<br>CDK125_8R biol rep 1 28551488 reads 22846753 uniquely mapped<br>CDK125_8R biol rep 2 31874070 reads 22565217 uniquely mapped<br>CDK125_8R input DNA 22972757 reads 19848262 uniquely mapped<br>CDK200_8R biol rep 1 26055930 reads 22424150 uniquely mapped<br>CDK200_8R biol rep 2 26774582 reads 23378582uniquely mapped<br>CDK200_8R input DNA 29585695 reads 24973293 uniquely mapped<br>Dph015_8R biol rep 1 316977776 reads 69306930 uniquely mapped<br>Dph015_8R biol rep 2 357694660 reads 76678049 uniquely mapped<br>Dph015_8R input DNA 1 296411680 reads 63209745 uniquely mapped<br>Dph015_8R input DNA 2 282271924 reads 60224901 uniquely mapped<br>YY1 biol rep 1 70070088 reads 7608643 uniquely mapped<br>YY1 biol rep 2 28118223 reads 23012458 uniquely mapped<br>YY1 input DNA 19327590 reads 16515891 uniquely mapped |
| Antibodies | The rabbit polyclonal anti-GFP antibody used for CHiP-seq is available from Abcam: ab290. |
| Peak calling parameters | bowtie2 was used for mapping with the parameters --very-sensitive -p 16 -x hg38, with hg38 and index of the hg38 copy of the human genome. For peak calling, MACS2 was run with the parameters -B -q 0.01 and the sequenced input DNA used as a control |
| Data quality | A positive control with known specificity (YY1) and consistency between replicates was assessed. The followgin are the number of identified peaks above 5 fold enrichment and 5% FDR:<br>CDK125_8R 135439<br>CDK125_8Q 17988<br>CDK200_8R 34059<br>YY1 8540<br>Dph015 8R 9175 |
| Software | ChIP-Seq analysis was done using bowtie2 version 2.2.5, samtools version 1.7, MACS2 version 2.2.7.1 and the MEME suite version 5.50. |

# Flow Cytometry

## Plots

Confirm that:

☒ The axis labels state the marker and fluorochrome used (e.g. CD4-FITC).

☒ The axis scales are clearly visible. Include numbers along axes only for bottom left plot of group (a 'group' is an analysis of identical markers).

☒ All plots are contour plots with outliers or pseudocolor plots.

☒ A numerical value for number of cells or percentage (with statistics) is provided.

## Methodology

| | |
|---|---|
| Sample preparation | Samples were prepared for analysis exactly as described in the methods. The source of the cells is listed above and there were no tissue processing steps used in this study. |
| Instrument | Sony SH800S |
| Software | Sony SH800 onboard software version 2.1.6 |
| Cell population abundance | Cells were not sorted in this study, FACS was used to measure single cell fluorescence. GFP negative cells ranged from ~10% of the total population to >90% of the measured population, depending on the ZFNs tested. |
| Gating strategy | In each experiment, a negative control (the empty plasmid backbone into which all ZFN constructs were cloned) was run in parallel to tested ZFNs. An initial 'negative' gate was defined where ~5% of the population was marked as GFP negative with this control. This same gate was used in every subsequent experiment. There was very little variance of this negative control from experiment to experiment as seen in Figure S10 B, C and D. |

☒ Tick this box to confirm that a figure exemplifying the gating strategy is provided in the Supplementary Information.

# Magnetic resonance imaging

## Experimental design

| | |
|---|---|
| Design type | *Indicate task or resting state; event-related or block design.* |
| Design specifications | *Specify the number of blocks, trials or experimental units per session and/or subject, and specify the length of each trial or block (if trials are blocked) and interval between trials.* |
| Behavioral performance measures | *State number and/or type of variables recorded (e.g. correct button press, response time) and what statistics were used to establish that the subjects were performing the task as expected (e.g. mean, range, and/or standard deviation across subjects).* |

## Acquisition

| | |
|---|---|
| Imaging type(s) | *Specify: functional, structural, diffusion, perfusion.* |
| Field strength | *Specify in Tesla* |
| Sequence & imaging parameters | *Specify the pulse sequence type (gradient echo, spin echo, etc.), imaging type (EPI, spiral, etc.), field of view, matrix size, slice thickness, orientation and TE/TR/flip angle.* |
| Area of acquisition | *State whether a whole brain scan was used OR define the area of acquisition, describing how the region was determined.* |

Diffusion MRI ☐ Used ☐ Not used

## Preprocessing

| | |
|---|---|
| Preprocessing software | *Provide detail on software version and revision number and on specific parameters (model/functions, brain extraction, segmentation, smoothing kernel size, etc.).* |
| Normalization | *If data were normalized/standardized, describe the approach(es): specify linear or non-linear and define image types used for transformation OR indicate that data were not normalized and explain rationale for lack of normalization.* |
| Normalization template | *Describe the template used for normalization/transformation, specifying subject space or group standardized space (e.g. original Talairach, MNI305, ICBM152) OR indicate that the data were not normalized.* |
| Noise and artifact removal | *Describe your procedure(s) for artifact and structured noise removal, specifying motion parameters, tissue signals and* |

Noise and artifact removal | physiological signals (heart rate, respiration).

Volume censoring | *Define your software and/or method and criteria for volume censoring, and state the extent of such censoring.*

## Statistical modeling & inference

Model type and settings | *Specify type (mass univariate, multivariate, RSA, predictive, etc.) and describe essential details of the model at the first and second levels (e.g. fixed, random or mixed effects; drift or auto-correlation).*

Effect(s) tested | *Define precise effect in terms of the task or stimulus conditions instead of psychological concepts and indicate whether ANOVA or factorial designs were used.*

Specify type of analysis: ☐ Whole brain ☐ ROI-based ☐ Both

Statistic type for inference
(See Eklund et al. 2016) | *Specify voxel-wise or cluster-wise and report all relevant parameters for cluster-wise methods.*

Correction | *Describe the type of correction and how it is obtained for multiple comparisons (e.g. FWE, FDR, permutation or Monte Carlo).*

## Models & analysis

| n/a | Involved in the study |
| --- | --- |
| ☐ | ☐ Functional and/or effective connectivity |
| ☐ | ☐ Graph analysis |
| ☐ | ☐ Multivariate modeling or predictive analysis |

Functional and/or effective connectivity | *Report the measures of dependence used and the model details (e.g. Pearson correlation, partial correlation, mutual information).*

Graph analysis | *Report the dependent variable and connectivity measure, specifying weighted graph or binarized graph, subject- or group-level, and the global and/or node summaries used (e.g. clustering coefficient, efficiency, etc.).*

Multivariate modeling and predictive analysis | *Specify independent variables, features extraction and dimension reduction, model, training and evaluation metrics.*
